# Associations between device-measured physical activity and performance-based physical function outcomes in adults: a systematic review and meta-analysis

**DOI:** 10.1136/bmjph-2023-100000

**Published:** 2023-10-30

**Authors:** Joshua Culverhouse, Melvyn Hillsdon, Brad Metcalf, Michael Nunns, Rebecca Lear, Gemma Brailey, Richard Pulsford

**Affiliations:** 1 Public Health and Sport Sciences, University of Exeter, Exeter, UK; 2 Medical School, University of Exeter, Exeter, UK

**Keywords:** epidemiology, public health, preventive medicine

## Abstract

This systematic review and meta-analysis aimed to examine the association between device-measured physical activity (PA) and performance-based measures of physical function (PF). Databases searched included CINAHL, Embase, MEDLINE/PubMed, SPORTDiscus, and Web of Science (last search conducted on November 11, 2022). Observational studies (cross-sectional or prospective) reporting associations between wearable device-measured PA and PF outcomes in non-clinical adults were eligible. Forty-two studies with a pooled sample of 27 276 participants were eligible, with 34 studies reporting a standardised regression coefficient (β) between at least one of four PA measures and one of six PF outcomes. All measures of PA were positively associated with all measures of PF, except for step count with grip strength. Largest associations were seen with lower-body PF tests; gait speed (βs=0.11–0.26), walk tests (βs=0.18–0.41), chair-rise test (βs=0.10–0.26), balance (βs=0.07–0.24) and Timed Up-and-Go (βs=0.10–0.24) all p<0.01. Small or no association was seen with grip strength (βs=0.02–0.07). In observational studies of general adult populations, there were associations between multiple dimensions of PA and a broad range of PF measures. The findings provide provisional support for the use of device measures of movement to remotely monitor people for risk of low PF. Prospective designs are needed to determine the direction of the relationship. Future studies should also explore a broader range of PA metrics beyond simple aggregate measures of time spent at different acceleration values as there is evidence that the temporal distribution of activity is related to health and functional outcomes.

## Introduction

 The increase in healthy life expectancy has not kept pace with the increase in absolute life expectancy, resulting in a greater proportion of years lived in poor health.^1^ The UK’s latest figures suggest between 16 and 19 years of life will be lived in poor health for males and females, respectively.^2^ The societal and economic burden of these additional years lived in poor health is hard to quantify, but health and social care costs are rising, with the increased demand in later life in part due to loss of independence and disability.^3^

Physical function (PF) is a broad concept that relates to the capacity of an individual to perform the physical tasks of everyday life required for independence,^4^ which reflects motor function and control, and components of physical fitness.^5^ Disablement models support the causal pathway from limitations in PF to disability, and loss of independence once these limitations interfere with activities of daily living.^6 7^ Relatively simple performance-based measures of PF such as grip strength, gait speed, chair rise tests, walk tests and balance can be strong predictors of adverse future health outcomes in older adults^8^,^11^ and late mid-life.^12^ Weak grip strength and slow gait speed are also characteristics of Fried’s frailty phenotype.^13^ Chair rise tests and grip strength have been recommended as screening and diagnostic tools for sarcopenia.^14^ However, PF assessments largely take place in clinical settings and only tend to occur when a person is attending a medical setting due to an adverse health event.

Declining PF is a common factor of ageing and, despite impairments typically being considered in older age, they can occur much earlier in mid-life (45–64 years).^15^ Depending on the point of intervention, declines in PF can potentially be prevented, retarded or reversed.^16^ However, identifying opportunities to intervene in mid-life relies on the ability to detect impairments in function prior to the point that reduced function results in presentation in medical settings. Remote health monitoring, through wearable devices, is one possible solution to early detection of presymptomatic and preclinical changes in PF.^17^ Wearable devices for monitoring health outcomes are already being employed by both individuals, to track their own health through activity levels, and by clinicians as a method of early detection.^18^

Wearable devices, such as accelerometers, have become increasingly popular for measuring physical activity (PA) in health research.^19^ There is strong evidence that structured PA, defined as any bodily movement produced by skeletal muscles that results in energy expenditure,^20^ and exercise interventions can improve or delay the loss of PF in older adults.^21 22^ Therefore, it is reasonable to consider that PA measures may be a potential proxy for PF. Prior to this, it is necessary to know what measures of PA are most strongly associated with, or even predictive of, PF. However, there is a paucity of evidence on the association between PA and PF in mid-life, when function is likely to be good but declining.

Systematic review level evidence of the associations between free-living PA and PF is limited, with reviews often focussing on interventions in people with reduced function.^21 22^ A meta-analysis has shown light intensity (LPA) and moderate-to-vigorous intensity PA (MVPA) to be associated with grip strength and chair rise tests^23^; however, the focus of the meta-analysis was on the association between PA and strength rather than PF. In addition, included studies were limited to older adults, preventing insight into important associations of PA and PF in midlife. It also included a mix of studies of healthy populations as well as studies of specific clinical populations (eg, chronic obstructive pulmonary disease, diabetes, osteoarthritis), where the association between PA and PF might be confounded by these long-standing health conditions. No analysis of the differences in associations between studies of healthy and clinical populations was performed. To the authors’ knowledge, there are no systematic reviews of the association between PA and PF indicators such as gait speed, walk tests, balance or the Timed Up-and-Go test (TUG); and no reviews that examine the associations of PA and PF in both mid-life and older adulthood. This systematic review and meta-analysis examines associations between wearable, device-measured PA and a range of performance-based PF outcomes in non-clinical adults. The findings will inform the potential of remote monitoring of early declines in PF, which could inform the development of future screening programmes and interventions.

## Methods

The review was conducted according to the Conducting Systematic Reviews and Meta-Analyses of Observational Studies of Etiology (COSMOS-E)^24^ guidance and the Cochrane handbook^25^; and reported according to the Preferred Reporting Items for Systematic Reviews and Meta-Analyses guidelines.^26^ The protocol was registered in the International Prospective Register of Systematic Reviews—PROSPERO (CRD42021282861).

### Search strategy

Systematic literature searches were conducted in PubMed (including Ovid MEDLINE, HMIC and Embase), EBSCOhost (including CINAHL and SPORTDiscus) and Web of Science for studies published between database inception and 15th June 2021; a top-up search was performed on 11 November 2022. The search strategy included keywords related to PA, device-based measures of PA, PF outcomes and observational study designs (online supplemental file 1). In addition, supplementary searches were performed through bibliography screening of included papers to identify any other potentially relevant publications.

### Study selection

Inclusion was determined by two independent reviewers (JC+GB or RL). Disagreements were resolved by discussion with the third author (GB or RL), if required. Study selection was completed in two phases: title and abstract screening was performed to exclude clearly irrelevant studies, after which full texts were screened. If two or more studies reported similar associations for the same cohort, we included the study with highest quality score or largest sample size, respectively.

### Eligibility criteria

#### Population

Participants were adults (≥18 years of age) recruited from non-clinical, community-dwelling populations. Studies of adults recruited specifically due to the presence of, or expected progression to, a disease or other clinical condition were excluded. These inclusion criteria allow for generalisation to the general population, including those in mid-life; these assertions cannot be made from studies of clinical populations of solely older adults.

#### Exposure

Studies reporting continuous wear data from remote wearable, device-based measures of PA were included. Depending on device, this included studies that advised participants to wear the device for 24 hours continuously, or to only remove the device during sleep and water-based activity. Reported PA metrics were total step count, total volume of PA (TPA), LPA and MVPA, classified using published cut-points or proprietary algorithms. Studies which collapsed continuous PA data were contacted to try to obtain the continuous association. We excluded studies that exclusively reported estimates of sedentary behaviour.

#### Outcome

Studies reporting performance-based PF instruments, adopted by clinicians and researchers, were included. These include; grip strength, gait speed, chair rise tests, walk tests, balance tests or composite assessments of these measures.^14 27 28^

#### Study design

The review included observational studies (both cross-sectional and prospective designs), which reported associations between the exposures and outcomes. Experimental studies and randomised controlled trials were excluded.

No restrictions were placed on country or date. Only full texts, in English, were included.

#### Data extraction

Two authors (JC+RL) independently extracted the following data from included studies: (1) author, study year and country of origin; (2) cohort and study design; (3) sample size and sex distribution; (4) age of study participants; (5) device used for PA measurement and metrics reported; (6) test used for assessing PF and metrics reported; (7) statistical analyses undertaken including and covariates included and (8); key results for the association between PA and PF. Discrepancies in extracted data were resolved by discussion with a third author (GB), if required.

#### Assessment of study and evidence quality

Two authors (JC+RL) independently assessed the quality of included studies using an adapted version of the National Institutes of Health Quality Assessment Tool for Observational Cohort and Cross-Sectional Studies (online supplemental file 2). Scores were given ranging from 0 to 12, with higher scores indicating higher quality. Discrepancies in quality assessment were resolved by discussion with a third author (GB), if required. The continuous quality rating scores were used in sensitivity analyses.

#### Statistical analysis

The required association statistic was the standardised regression coefficient (β) and SE, see detailed explanation of β coefficient below. Using the β coefficient allowed for synthesis across different metrics and units for both PA and PF variables. If only a partial correlation coefficient was obtainable, this was used as an approximation the β coefficient, with sensitivity analysis performed to ensure these coefficients would not bias the pooled effect.^29^

Some PF outcomes have slight different measurement protocols, and these are grouped together in this review as follows; the chair-rise test outcome includes the 30 s and the five-repition variants; gait speed includes any protocol measuring normal/usual or maximal gait speed over a distance ≤10 m; grip strength includes any protocol using a hand dynamometer to obtain maximal grip strength; walk tests included the 6 min walk test and 400 m walk test, or any variant covering a similar time or distance in different units; the TUG test includes both the 8-foot and 3 m variations; and balance includes any continuous measure of tandem, semi-tandem or single-leg stance, with eyes closed or open. Where composite scores of the above measures were reported for an overall PF score, we sought to obtain the associations for the individual components.

The adjusted β coefficients were extracted from included papers, or obtained from converting the unstandardised regression coefficient (b) where possible using the following equations: $\beta =\frac{SD_{x}}{SD_{y}}\;b$ and $SE(\beta )=\frac{SD_{x}}{SD_{y}}SE(b)$

where SD_x_ is the SD of the PA exposure and SD_y_ is the SD of the PF outcome.^30^ If the SD_x_ or SD_y_ was reported in two subgroups and needed to be combined the following equation was used to obtain the full sample SD:

$$SD_{full\;sample}=\sqrt{\frac{(n_{1}-1)SD_{1}^{2}+(n_{2}-1)SD_{2}^{2}+\frac{n_{1}n_{2}}{n_{1}+n_{2}}(M_{1}-M_{2}{)}^{2}}{n_{1}+n_{2}-1}}$$

where *n*_1_ and *n*_2_ are the sample sizes of the two subgroups, SD_1_ and SD_2_ are the subgroup SDs, and M_1_ and M_2_ are the subgroup means.^28^ If SE was not reported, it was calculated from the 95% CIs using the following equation:

SE=(upper limit−lower limit)/3.92

where the upper limit and lower limit refer to the 95% CI of the effect size.^31^ In cases where the partial correlation is used, the following equation was used to calculate the SE of the partial correlation:

$$SE=\frac{1-r^{2}}{\sqrt{n-1}}$$

where *r* is the partial correlation coefficient and *n* is the sample size.^31^ If a study reported associations separately for two subgroups (eg, males and females) these were combined using the following equations to provide a composite effect size:

$$\beta_{p}=(W_{1}\beta_{1}+W_{2}\beta_{2})/(W_{1}+W_{2})$$

$$SE(\beta_{p})=\sqrt{\frac{1}{W_{1}+W_{2}}}$$

where β_1_ and β_2_ are the β coefficients for the two subgroups, and SE(β_1_) and SE(β_2_) are the respective SEs. The weightings for the two subgroups are W_1_=1/SE(β_1_))^2^ and W_2_=1/SE(β_2_))^2^.^31^

Where required, we contacted authors to request the β coefficient adjusted for age+sex, or additional unpublished data to allow us to estimate the β coefficient from the effect size published in the paper. If authors had measured additional PA or PF outcomes but not reported these associations, these were also requested. β coefficients were inversed for PF outcomes where a lower score indicated better function, so that all positive effects in this review indicate better/higher PF.

Meta-analyses were performed to obtain a pooled estimate of individual β coefficients for associations between the reported PA measures and PF outcomes, visualised as forest plots. Ideally, included effect sizes would be adjusted for the same covariates^31 32^; however, due to varying adjustment models across papers, the included estimates were extracted from the following order of models: (1) age+sex and (2) age, sex+additional factors. We used random-effects models to account for both between and within study variance, with inverse variance as the weighting method. Statistical heterogeneity was estimated using the I^2^ analysis. An I^2^ (the variation across studies due to heterogeneity rather than chance) of <40% was considered low heterogeneity and an I^2^ of >75% was considered high heterogeneity.^25^ Heterogeneity, along with the number of studies within each meta-analysis should be considered when interpreting the pooled effects. Where possible (≥10 studies in the meta-analysis^25^) meta-regressions were run to examine the individual effects of sex (percentage female), age, quality assessment and study sample size (n) on the associations.

#### Sensitivity analyses

Leave-one-out sensitivity was performed on each meta-analysis to explore the influence of individual studies on the overall pooled effect. In addition, for meta-analyses with ≥10 studies, a visual and statistical evaluation of publication bias was performed using funnel plots and Egger’s regression tests (p<0.05 indicated publication bias).^33^ For the purpose of quantifying the magnitude of the pooled effect size, the following values were used: 0.10–0.19=small, 0.20–0.29=medium and ≥0.30=large.^31^ So as not to entirely exclude them from the review, studies for which a β coefficient was not obtained were included in a vote count summary and the directions of associations compared with those studies included in the meta-analysis via χ^2^ test. All analyses were performed in Stata V.17 (StataCorp. 2021. Stata Statistical Software: Release 17. College Station, StataCorp).

## Results

### Search and study selection results

**Figure 1:**
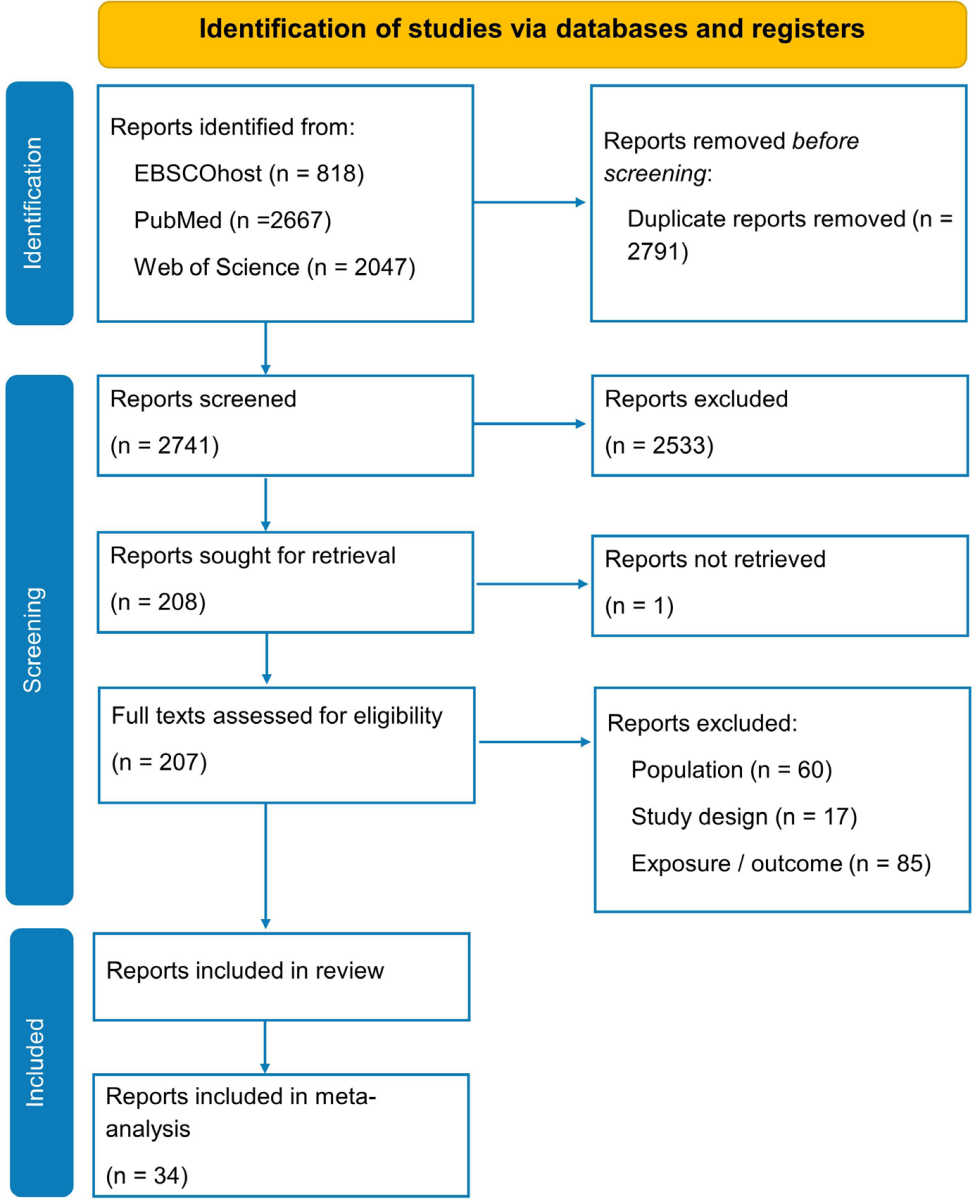
PRISMA flow diagram showing the screening process and the search results. PRISMA, Preferred Reporting Items for Systematic Reviews and Meta-Analyses.

The original and top-up database searches identified 2741 articles after duplicates were removed, of which 2533 were excluded based on title and abstract screening.^26^ Two hundred and seven full-text articles were reviewed, 43 of which fulfilled the inclusion criteria. Two studies, by the same author, used data from the same pool of participants,^34 35^ the study with the larger sample size and greater number of reported associations was chosen for inclusion.^34^ Resulting in a total of 42 included publications (figure 1).

### Study characteristics

The 42 included studies represented N=27 276 participants (range: n=64–4702), with an average mean sample age of 70.3 years (range: 46–90 years) (table 1). Study samples were on average 63.6% female. Three studies were prospective^36^,^38^ and the other 39 were cross-sectional.^3439^,^75^ Most studies used accelerometers to measure PA (k=39), with one study using a pedometer,^68^ and two using the Actiheart combined accelerometer and heart rate sensor.^43 70^ Device locations across studies were as follows; hip/waist (k=27), thigh (k=3), wrist (k=3), other (k=9). Studies reported the following PA dimensions; MVPA (k=31), LPA (k=17), TPA (k=15) and average or total step count (k=14). A range of accelerometer cut-points were used for classifying LPA and MVPA across studies, the most common non-proprietary classifications were Troiano^76^ (k=6) and Freedson^77^ (k=5) (online supplemental file 3, table 2).

**Table 1:** Characteristics of articles assessing the association between device-measured physical activity metrics with performance-based physical function outcomes in adults

Author (year), country	Cohort	Design	Sample(n)	Age	Sex (F%)	PA measures				PF measures							Adjustments		
						LPA	MVPA	Steps	TPA	Bal.	Chair	Gait	HGS	TUG	Walk	Comp.	Age	Sex	Add.
Adachi (2018), JP^39^	N/R	CS	308	79.9 (3.6)	100		✓	✓				✓					✓	n/a	✓
Aggio (2016),* GB^50^	BRHS	CS	1286	78.2 (4.5)	0	✓	✓					✓	✓				✓	n/a	✓
Aoyagi (2009), JP^61^	Nakanojo	CS	170	72.6 (4.6)	55.3			✓	✓	✓		✓	✓				✓	✓	
Cooper (2015), GB^70^	NSHD	CS	1727	63.3 (1.1)	51.5		✓		✓	✓	✓		✓	✓				✓	
Cooper (2020), GB^71^	BCS70	CS	4702	46 (0)	52.4		✓		✓				✓				n/a	✓	✓
Davis (2014), GB^72^	Project OPAL	CS	217	78.1 (5.8)	50.2		✓			✓	✓	✓					✓	✓	✓
Duck (2019), USA^73^	N/A	CS	99	74 (6.5)	78.2	✓	✓			✓				✓			✓	✓	✓
Gobbo (2020), BR^36^	N/A	PR	68	69.4 (6.5)	70.9		✓					✓	✓	✓			✓		✓
Hall (2017), USA^74^	MURDOCK	CS	775	62.1 (N/R)	53.2	✓	✓	✓		✓	✓	✓			✓				
Hsueh (2020), TW^34^	N/A	CS	127	70.8 (5.3)	71.7		✓	✓	✓	✓	✓	✓	✓	✓			✓		✓
Izawa (2017), JP^75^	N/A	CS	290	74.5 (N/R)	37.6		✓			✓		✓		✓			✓		✓
Jantunen (2017), FI^40^	HBCS	CS	695	70.7 (2.7)	54.5	✓	✓				✓				✓	✓	✓	✓	
Johansson (2021), NO^41^	Tromsø	CS	3653	68.5 (5.9)	51	✓	✓				✓		✓				✓		
Kim (2015), JP^42^	N/R	CS	207	83.5 (2.6)	55.6				✓			✓	✓				✓	✓	
Kruger (2016), SA^43^	PURE	CS	247	57.0 (10.2)	100				✓			✓	✓				✓	n/a	✓
Lai (2020), TW^44^	N/A	CS	118	70.0 (5.0)	70.3		✓				✓	✓	✓	✓			✓	✓	✓
Lerma (2018), USA^45^	N/A	CS	91	70.7 (10.2)	60.4	✓	✓				✓	✓			✓	✓	✓	✓	✓
Lohne-Seiler (2016), NO^46^	N/A	CS	161	72.8 (5.1)	52.8			✓		✓			✓				✓	✓	✓
Manas (2019),* ES^47^	TSHA	CS	771	76.8 (4.9)	54.0	✓	✓									✓	✓	✓	✓
Meier (2020), USA^48^	N/A	CS	304	72.8 (5.8)	58.2			✓				✓	✓				✓	✓	✓
Mendham (2021), SA^49^	N/A	CS	111	67(64, 71)	100.0	✓	✓		✓			✓	✓	✓	✓		✓	n/a	
Mizumoto (2015),* JP^37^	PIPAOI	PR	201	79.7 (3.8)	58.7		✓	✓				✓	✓				✓	✓	✓
Nagai (2018),* JP^51^	N/A	CS	886	73.6 (7.0)	70	✓	✓					✓	✓						
Oguma (2017)*, JP^52^	TOOTH	CS	155	90.2 (1.4)	52.6			✓	✓	✓	✓		✓	✓					
Osuka (2015), JP^53^	N/A	CS	802	72.5 (5.9)	76.7	✓	✓			✓	✓			✓			✓	✓	✓
Pina (2021), SA+GB^54^	N/A	CS	288	68.5 (N/R)	79.9	✓	✓		✓			✓	✓				✓	✓	✓
Reid (2016),* AU^55^	AusDiab	CS	602	58.1 (10.0)	58.5	✓	✓	✓						✓			✓	✓	✓
Ribeiro (2020), BR	N/A	CS	230	*66*(*63, 71*)	70.4											✓	✓	✓	✓
Rojer (2018), NL^78^	N/A	CS	236	66.9 (N/R)	64.8			✓	✓			✓	✓				✓	✓	
Sanchez-Sanchez (2019), ES^57^	TSHA	CS	497	78.1 (5.7)	54.3	✓	✓		✓			✓	✓				✓	✓	✓
Santos (2012), PT^58^	N/A	CS	312	74.3 (6.6)	62.5		✓				✓			✓	✓		✓	✓	✓
Savikangas (2020), FI^59^	PASSWORD	CS	293	74.4 (3.8)	58.4	✓	✓					✓			✓	✓	✓	✓	
Schrack (2019), USA^60^	BLSA	CS	680	67.9 (13.2)	49.9				✓			✓			✓	✓	✓	✓	✓
Spartano (2019), USA^62^	FOS	CS	1352	68.6 (7.5)	54		✓	✓			✓	✓	✓				✓	✓	✓
Thiebaud (2020),* JP^63^	N/A	CS	86	67 (7)	100	✓	✓					✓					✓	n/a	✓
van der Velde (2017), NL^64^	Maastricht	CS	1962	59.7 (8.2)	48.6		✓		✓		✓		✓		✓		✓	✓	✓
Ward-Ritacco (2014), USA^65^	N/A	CS	64	58.6 (3.6)	100		✓	✓			✓			✓	✓		✓	n/a	✓
Ward-Ritacco (2020), USA^66^	N/A	CS	80	52.6 (6.1)	100			✓			✓			✓	✓		✓	n/a	✓
Westbury (2018), GB^67^	HSS	CS	131	78.8 (2.4)	75.6		✓		✓			✓	✓					✓	
Yamada (2011),* JP^68^	N/A	CS	515	77.0 (7.2)	67.5			✓		✓	✓	✓		✓					
Yasunaga (2017), JP^69^	N/A	CS	287	74.4 (5.2)	37.3	✓	✓			✓		✓	✓	✓			✓	✓	✓
Yerrakalva (2022), UK^38^	EPIC-Norfolk	PR	1488	69.9 (6.0)	54.4	✓	✓		✓		✓	✓	✓				✓	✓	✓

Age in years is presented as mean (standard deviationSD) or median [(interquartile rangeIQR]). Sex distribution is presented as the percentage of females within the study sample.

* Asterisk denotes not included in meta-analyses.

AddadditionalAUAustraliaBalbalance testBLSABaltimore Longitudinal Study of AgingBRBrazilBRHSBritish Regional Heart StudyChairchair rise testCompcomposite measureCScross-sectionalESSpainFIFinlandFOSFramingham Offspring StudyGaitgait speedGBGreat BritainHBCSHelsinki Birth Cohort StudyHGShandgrip strengthHSSHertfordshire Sarcopenia StudyJPJapanLPAlight intensity physical activityMURDOCKThe Measurement to Understand the Reclassification of Disease Of Cabarrus/KannapolisMVPAmoderate-to-vigorous physical activityN/Anot applicableNLNetherlandsNONorwayN/Rnot reportedNSHDNational Survey of Health and DevelopmentOPALOlder People and Active LivingPAphysical activityPFphysical functionPIPAOIPopulation-Based and Inspiring Potential Activity for Old-Old InhabitantsPRprospectivePTPortugalPUREProspective Urban and Rural Epidemiological studySASouth AfricaSPPBshort physical performance batteryStepsaverage or total step countTOOTHThe Tokyo Oldest-Old Survey of Total HealthTPAtotal physical activityTSHAToledo Study of Healthy AgingTUGTimed Up-and-GoTWTaiwan

**Table 2:** Vote counting across all reported associations of included studies

	↑		↓		↔		Total
	n (%)		n (%)		n (%)		n
All studies (n=42)	155	(65.4)	1	(0.0)	79	(32.9)	237
Subgroup vote count							
Included in MA (n=34)	131	(64.2)	1	(0.1)	70	(33.8)	204
Excluded from MA (n=8)	24	(72.7)	0	(0.0)	9	(27.3)	33

↑ = Significant positive association; ↓ = significant negative association; ↔ = no association.

MAmeta-analysis

Studies also reported the following PF outcomes; gait speed (k=27), handgrip strength (k=24), chair rise tests (k=17), TUG (k=15), balance (k=12), endurance walk tests (k=10) and composite PF tests (k=6) (online supplemental file 3). There was an insufficient number of studies employing composite measures of PF for these to be pooled; only one of the four studies that did report composite measures was excluded from meta-analyses, where the associations of individual measures within the composite score were not reported or obtainable.^47^

Of the 42 studies identified for inclusion in this review, a standardised regression coefficient (β), adjusted for at least age+sex, was obtained for 34 studies and thus were included in pooled analyses. Authors of 14 of these studies provided either additional data to allow the estimation of the β coefficient, or effect sizes for additional associations that were not reported in the original paper. The variations in PA exposures and PF outcomes reported across included studies prevented the computation of a single overall effect size. Instead, multiple pooled analyses (n=24) were performed for each combination of PA and PF measure, as described above. Overall, the 34 studies included in meta-analyses represent 22 774 participants (range: 64–4702), with a mean sample age of 69.3 (range: 46–83.5) and comprising 63.4% females. Two studies reported prospective associations^36 37^ and 32 reported cross-sectional associations.^3439^,^41 43^ The limited number of studies reporting some of the associations meant that only 6 of the meta-analyses contained ≥10 studies, and therefore, meta-regressions and Egger’s test were only performed on these 6. Due to an unbalanced number of studies across the device locations (27 studies adopted waist/hip), we refrained from conducting subgroup analysis on this factor. All extracted data are provided in online supplemental file 3.

### Methodological quality

For all 42 included studies, the mean quality assessment rating was 8.1±1.2 (range: 3–13). For the 34 studies included in meta-analyses, the mean rating was 8.2±1.2 (range: 6–13). Study design (only 4 studies were prospective), sample size justification and participation rate of eligible persons were the most problematic domains of study quality (online supplemental file 4).

## Results of meta-analyses

### Gait speed

**Figure 2:**
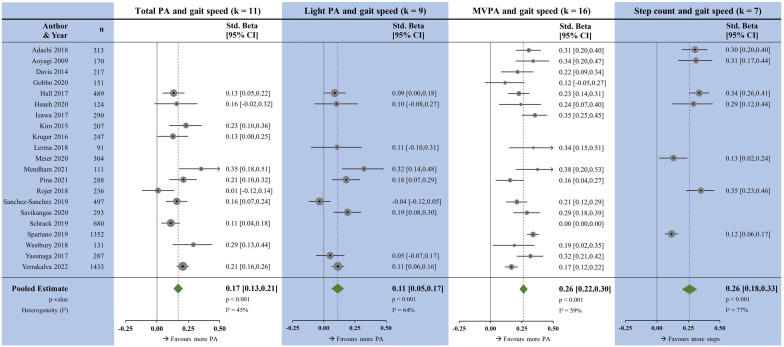
Forest plots showing the associations between physical activity measures and gait speed. k, number of studies per meta-analysis; MVPA, moderate-to-vigorous physical activity; N, sample size; PA, physical activity.

There were positive associations for each of the PA measures with gait speed (figure 2). The magnitudes of association varied between PA measures, with medium strength associations seen in MVPA (β=0.26, p<0.001) and step count (β=0.26, p<0.001), and small associations seen with TPA (β=0.17, p<0.001) and LPA (β=0.11, p<0.001). Statistical heterogeneity was high step count, and moderate for TPA, LPA and MVPA. Meta-regressions for age, sex, sample size and quality assessment score for TPA and MVPA were non-significant (online supplemental file 6). Egger’s test for TPA and MVPA was non-significant (online supplemental file 7).

### Chair rise tests

**Figure 3:**
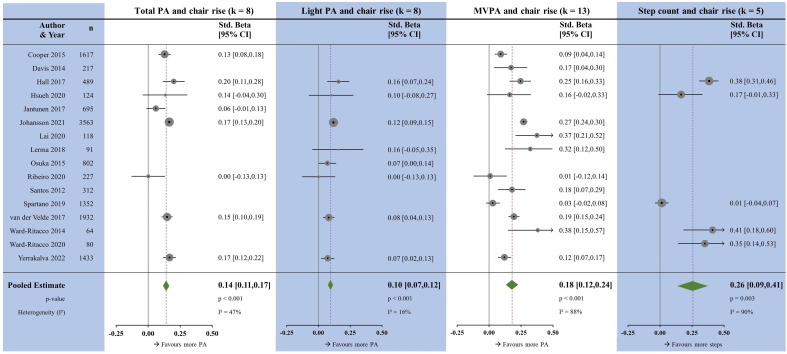
Forest plots showing the associations between physical activity measures and chair rises. k, number of studies per meta-analysis; MVPA, moderate-to-vigorous physical activity; N, sample size; PA, physical activity.

All PA measures were positively associated with chair rise tests (figure 3). The magnitudes of association varied between PA measures; step count was the largest but with wide CIs (β=0.26 (0.09 to 0.41), p=0.003), followed by MVPA (β=0.18, p<0.001), TPA (β=0.14, p<0.001) and LPA (β=0.10, p<0.001). Heterogeneity was high MVPA and step count, moderate for TPA and low for LPA. Meta-regressions for MVPA were non-significant (online supplemental files 67). Egger’s test for MVPA was non-significant.

#### Balance

**Figure 4:**
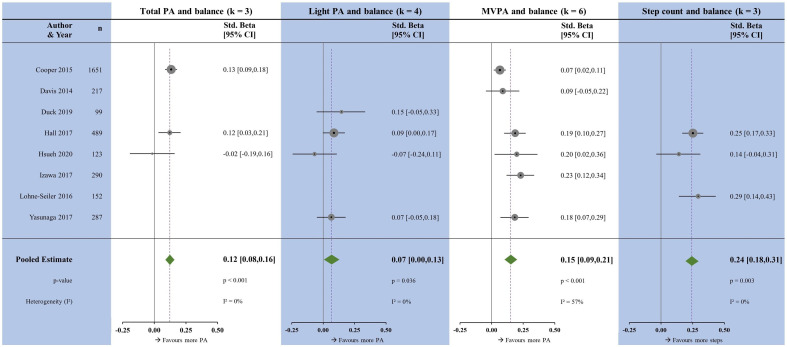
Forest plots showing the associations between physical activity measures and balance. k, number of studies per meta-analysis; MVPA, moderate-to-vigorous physical activity; N, sample size; PA, physical activity.

There were a limited number of studies reporting associations with balance. All measures of PA were positively associated with balance (figure 4). The largest associations were seen with step count (β=0.24, p=0.003), followed by MVPA (β=0.15, p<0.001) and TPA (β=0.12, p<0.001); the smallest association was with LPA (β=0.07, p<0.036). Heterogeneity was moderate for MVPA and low TPA, LPA and step count.

### Walk tests

**Figure 5:**
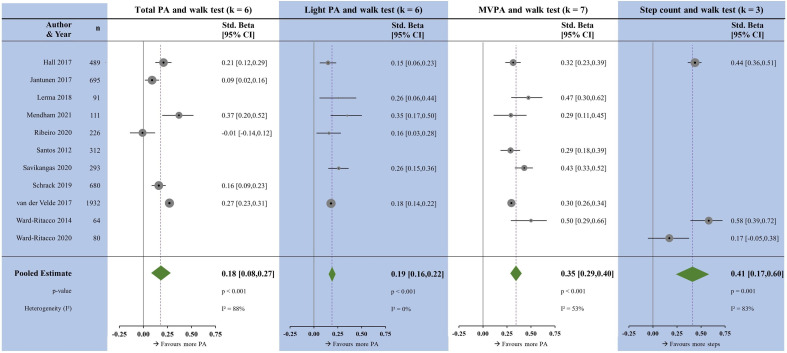
Forest plots showing the associations between physical activity measures and walk tests. k, number of studies per meta-analysis; MVPA, moderate-to-vigorous physical activity; N, sample size; PA, physical activity.

Similar to balance, there were a limited number of studies reporting associations with walk tests. All measures of PA were positively associated with walk tests (figure 5). The magnitudes were largest with step count (β=0.41, p=0.001) and MVPA (β=0.35, p<0.001); followed by LPA (β=0.19, p<0.001) and TPA (β=0.18, p<0.001). Heterogeneity was high for TPA and step count, moderate for MVPA and low for LPA.

### Timed Up-and-Go

**Figure 6:**
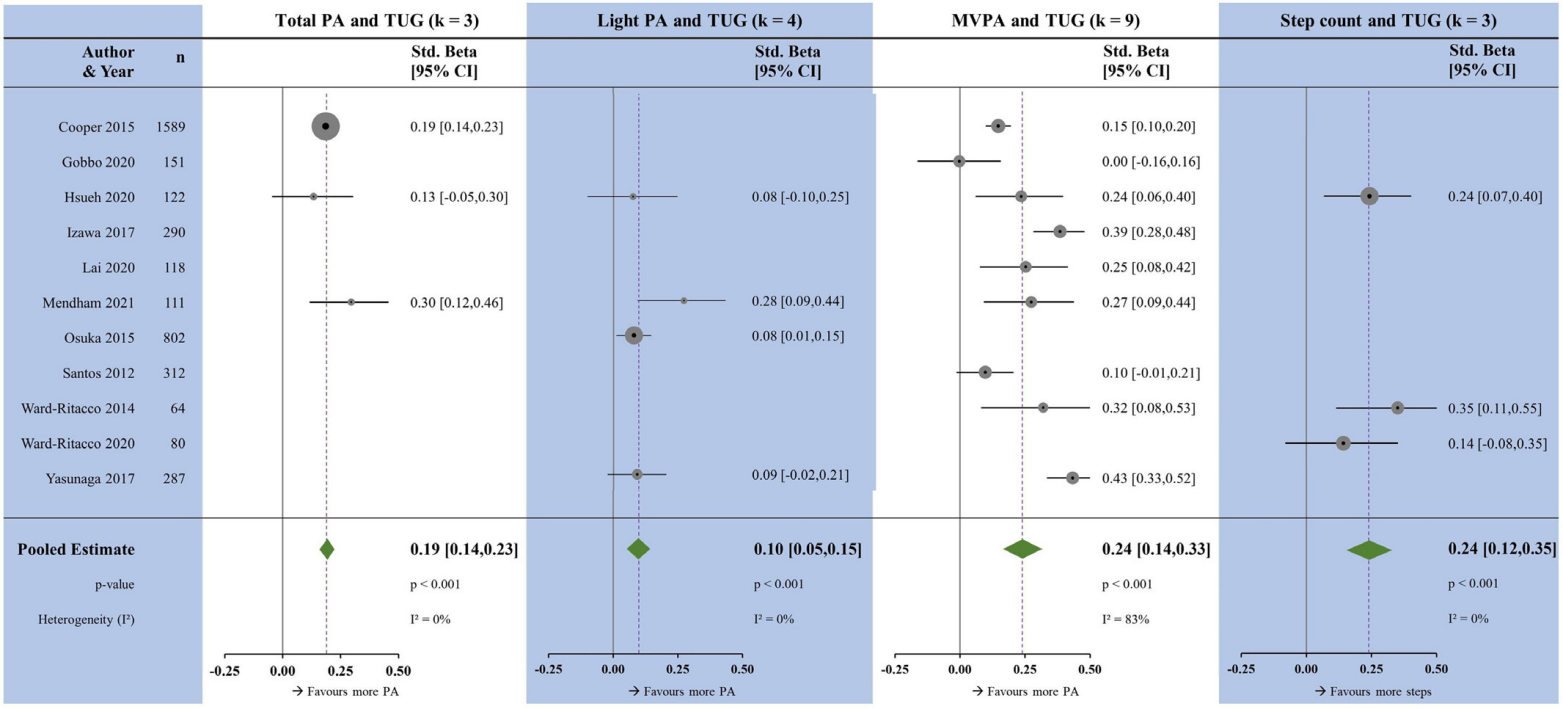
Forest plots showing the associations between physical activity measures and the timed up-and-go test. k, number of studies per meta-analysis; MVPA, moderate-to-vigorous physical activity; N, sample size; PA, physical activity.

All measures of PA were associated with the Timed Up-and-Go test (figure 6). The magnitudes were largest with MVPA (β=0.24, p<0.001) and step count (β=0.24, p<0.001); followed by TPA (β=0.19, p<0.001) and LPA (β=0.10, p<0.001). Heterogeneity was high for MVPA, and low for TPA, LPA and step count.

### Handgrip strength

**Figure 7:**
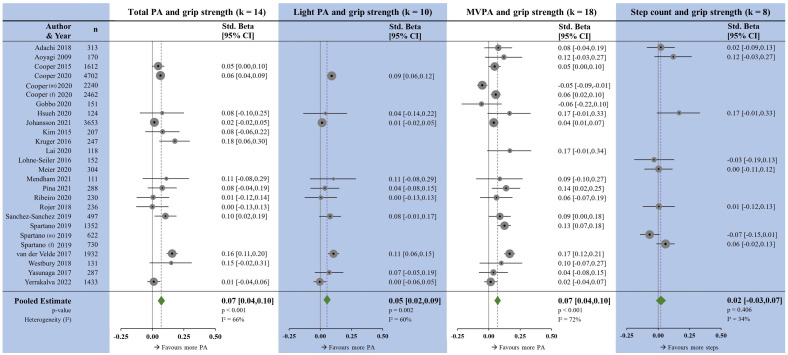
Forest plots showing the associations between physical activity measures and handgrip strength. k, number of studies per meta-analysis; MVPA, moderate-to-vigorous physical activity; N, sample size; PA, physical activity.

Handgrip strength showed small, positive associations with TPA (β=0.07, p<0.001), LPA (β=0.05, p=0.002) and MVPA (β=0.07, p<0.001), but had no association with step count (β=0.02, p<0.406) (figure 7). Heterogeneity was moderate for TPA, LPA and MVPA, and low for step count. Egger’s test for TPA, LPA and MVPA was non-significant (online supplemental file 7). As detailed in the methods, effect sizes from studies reporting subgroups were pooled, except in two instances for grip strength,^42 67^ where the effects were in the opposite direction in each subgroup (figure 7C,D.

### Sensitivity analyses

The results of the ‘leave-one-out’ sensitivity analyses suggests that, in general, our estimates of associations were robust to sensitivity analyses. The β coefficients did not change more than; −0.04 to +0.03 for balance, −0.04 to +0.08 for chair rise tests, −0.02 to +0.04 for gait speed, −0.03 to+0.03 for grip, −0.03 to +0.05 for TUG and −0.07 to+0.12 for walk tests. Importantly, β coefficients from the ‘leave-one-out’ analyses were always within the 95% CIs of the original estimates derived from ‘all studies’ (online supplemental file 5). Even for the three associations that became non-significant, the magnitude of the change in the β coefficient was very small (eg, β coefficients of 0.12, 0.07 and 0.41 fell no more than 0.04). The sample study sizes for these associations were 3, 3 and 4, respectively, and we are impacted when the studies with large sample sizes were removed; therefore, we suggest caution when interpreting the associations with smaller numbers of studies.

All meta-regressions were non-significant. Bubble plots suggested that some meta-regressions might have studies with high leverage. According to Borenstein *et al,*^79^ there are no current methods in which meta-regression deals with ‘high leverage’. Leverage was calculated for each study within each meta-regression, and the formula reported in Borenstein *et al* was used to identify studies with ‘high’ leverage. In the absence of an optimal process to deal with high leverage, analysis was re-run excluding any studies with high leverage. All meta-regressions remained non-significant.

### Vote count summary

Of the 42 studies that met the inclusion criteria, β coefficients were not obtainable for eight studies; and therefore, these were not included in the meta-analysis. To avoid completely omitting these studies from the review and to acknowledge any potential bias, a vote count summary is provided with all studies and subgroup vote count comparing those studies included in the meta-analysis and those excluded (table 2).

Overall, 237 associations across 24 potential associations were reported from the 42 included studies. A higher proportion of positive (higher PF) associations were observed in the studies not included in the meta-analyses (72.7%) compared with those included (64.2%). A χ^2^ test showed direction of association did not differ by included vs excluded associations, χ^2^=1.68, p=0.195

## Discussion

The aim of this systematic review was to examine associations between wearable, device-measured PA and a range of performance-based PF outcomes in community-dwelling adults. Forty-two studies met the inclusion criteria and 34 studies provided suitable data for meta-analyses, across 24 different associations between PA and PF. All measures of PA were positively associated with all measures of PF, except for step count with grip strength. In general, the more physically active people were the better their PF. Associations were generally higher with lower-body PF tests, particularly gait speed, chair rises and walk tests. Within each measure of PF, the associations with either MVPA or step count were generally larger than compared with LPA or TPA. The associations of PA with chair-rise tests and grip strength were similar to those reported in a previous meta-analysis.^23^ Direct comparisons between this review and that of Ramsey *et al*^23^ are not possible due to this review excluding studies that recruited studies based on the presence of a specific clinical condition. Our decision was taken to increase the external validity of the results but also because the expected association between PA and PF would be condition specific and there were too few studies for each specific condition to carry out analysis separately, comparing studies in healthy populations to each clinical condition. Our inclusion of all adults (not just older adults) adds to the previous review in this area. The number of studies within many of the meta-analyses did not allow for meta-regression; though in the six which did, there was no apparent effect of sample age on the observed associations.

The differences observed in the magnitude of associations between PA and specific measures of PF may be explained, at least in part, by the specificity of exercise. For example, grip strength, a general measure of muscular strength, would be expected to improve as a result of resistance type exercises rather than ambulatory activity. Therefore, measuring PA with devices that largely capture ambulatory behaviour, not resistance exercise, would likely underestimate the association between PA and grip strength, especially in participants undertaking a higher level of resistance exercise. Similarly, measures of PF more related to ambulation (eg, gait speed and walk tests) would be expected to produce larger associations with device-based measures of PA that mainly represent ambulatory activity. Although device-based measures of PA overcome recall and social desirability biases associated with self-report measures, they do not adequately capture strength or resistance-based activities.^80 81^

The reliance on single thresholds of acceleration to define activity intensity categories, for all study participants, can lead to the misclassification of time spent in different intensities of activity. The approach assumes that a given value of acceleration represents the same intensity of PA for all individuals regardless of their fitness.^82^ For example, if two people (one low fit and one high fit) were walking at the same speed on a treadmill the accelerometer would record approximately the same level of acceleration assuming both people had similar stride lengths. However, the less fit person would be exercising at a higher relative intensity (% of maximum) than the fitter person. Consequently, in less fit participants the single threshold method would lead to an underestimate of time spent in MVPA—misclassified as LPA, and for fitter participants an overestimate of time in MVPA. Further, the most common thresholds used by included studies were derived in calibration studies of young adults (<30 year old) which is unlikely to generalise to older populations with lower fitness levels.^76 77^ Our findings show that more time at higher acceleration values is associated with better function, but it is difficult to know what level of relative intensity these thresholds represent in the populations being studied, even though in general higher accelerations are correlated with higher V0^2^ levels. In addition, most of the effect sizes were not adjusted for TPA, meaning associations between time spent in MVPA and PF may be confounded by TPA if MVPA and TPA are highly correlated. Although there was some variation in the thresholds used to classify LPA an MVPA between the studies, this would not be expected to affect the pooled estimates reported as regardless of the thresholds used the participants who undertook more time at higher intensity PA would still record more minutes of accelerometer estimated MVPA compared with participants who undertook less time at higher intensity PA.

The reporting of PA volume alone ignores other dimensions of activity and the temporal distribution, including event-based outcomes of free-living behaviour.^83^ This is despite evidence that two people with the same volume of activity, accumulated in different patterns will vary in their risk of mortality,^84^ and that patterns (eg, number and duration of bouts of activity) may also be associated with PF.^85^ Developments in data processing allow for additional PA metrics to be derived from accelerometers, metrics that better reflect the frequency, duration, intensity and volume of PA, as well as how the PA was accumulated within and between days. It is also possible to estimate specific movements, for example, sit-to-stand posture transitions,^86^ which have not been widely reported in this literature, but which might be more relevant to certain measures of PF (eg, chair rise tests and TUG). The ability to detect more specific types of activity, such as postural transitions, particularly the ‘quality’ of these activities (eg, duration, velocity and power),^87^ holds promise for better understanding of links between specific device-measures of PA and PF. This in turns raises the potential for remote monitoring of PF in free-living settings rather than being reliant on clinic-based measures. It is already documented that clinic and laboratory measures of PF do not capture the same broad dynamic of free-living PF.^88 89^

Only two of the studies included in this meta-analysis reported prospective associations, meaning the direction of causation cannot be determined. It is logical that the relationship is somewhat bidirectional, given the likely cyclical relationship between impaired function, disability and reduced PA.^90^ Prospective associations between PA in mid-life and preserved PF at follow-up have been demonstrated, although with self-report measures of the exposure and outcome.^91^ Further examination of these prospective associations should be performed with device-measured PA, to avoid the biases associated with self-report. The association between PA and PF, or even prevalence of impairment, in mid-life is poorly understood, despite the potential for early screening and intervention.^15^ The WHO specifically refers to reduced gait speed and muscle strength as early markers for declines in intrinsic capacity, and emphasises the need for early detection to prevent these declines in capacity.^92^ Prospective studies with measures of both PA and function collected in mid-life are required to better understand whether device-based measures of PA in mid-life are associated with the risk of low function later in life.

### Strengths and limitations

To the authors’ knowledge, this is the first meta-analysis of the associations between device measured free-living PA and PF in observational studies of adults from mid-life to older adulthood. Specifically, this is the first review to examine pooled associations of PA with gait speed, walk tests, balance and TUG. We build on previous analyses of associations with grip strength and chair rise tests by focussing on non-clinical populations where associations are less likely to be confounded by the presence of health conditions. The multiple dimensions of PA and broad range of performance-based PF outcomes provides a comprehensive review of the relative magnitudes of PA associations between PF measures, and the associations of different PA dimensions within those measures. The inclusion of studies employing device-based measures removes the impact of error and bias associated with self-report measures from pooled effects. However, we note that the number of studies within certain analyses was low, contributing to considerable heterogeneity, and an inability to explore potential effect modifiers using meta-regression. As such we interpret the reported pooled effects of these meta-analyses with a degree of caution. Adopting the standardised regression coefficient as the effect size for the pooled analysis allowed for the inclusion of studies employing different statistical inference methods, measurement methods and descriptive statistics.^31^ However, only evidence of an association should be interpreted from a significant meta-analysis, as the strength of associations is not comparable across standardised regression output. The minimum adjustment model for inclusion was age+sex may have meant some important confounding factors were overlooked; however, it allowed inclusion of a greater number of studies than if the criteria had been stricter. We could not include eight studies within meta-analyses, however, the proportion of these studies reporting positive associations between PA and PF was similar to those included in meta-analysis.

## Conclusion

In community-dwelling adults, higher levels of PA regardless of intensity were associated with higher levels of a broad range of PF measures. These findings support the potential of device-based measures of movement being used to remotely monitor people for risk of low PF without the need to attend a clinic or laboratory. The cross-sectional nature of all but one study and the focus on older age populations prevents generalisability of these associations to younger populations. Future research should also investigate a broader range of potentially important PA measures, especially those that capture how PA is accumulated within and between days.

## supplementary material

10.1136/bmjph-2023-100000 online supplemental file 1

10.1136/bmjph-2023-100000 online supplemental file 2

10.1136/bmjph-2023-100000 online supplemental file 3

10.1136/bmjph-2023-100000 online supplemental file 4

10.1136/bmjph-2023-100000 online supplemental file 5

10.1136/bmjph-2023-100000 online supplemental file 6

10.1136/bmjph-2023-100000 online supplemental file 7

## References

[1] Marmot M (2020). Health equity in England: the Marmot review 10 years on. BMJ.

[2] Office for National Statistics (2022). Health state life Expectancies, UK 2018 to 2020. https://www.ons.gov.uk/peoplepopulationandcommunity/healthandsocialcare/healthandlifeexpectancies/bulletins/healthstatelifeexpectanciesuk/2018to2020.

[3] Government Office for Science (2016). Future of an ageing population. The Oxford Institute of Population Ageing.

[4] Painter P, Stewart AL, Carey S (1999). Physical functioning: definitions, measurement, and expectations. Adv Ren Replace Ther.

[5] Garber CE, Blissmer B, Deschenes MR (2011). Quantity and quality of exercise for developing and maintaining cardiorespiratory, musculoskeletal, and Neuromotor fitness in apparently healthy adults: guidance for prescribing exercise. Med Sci Sports Exerc.

[6] Verbrugge LM, Jette AM (1994). The Disablement process. Soc Sci Med.

[7] NAGI SZ (1964). A study in the evaluation of disability and rehabilitation potential: concepts, methods, and procedures. Am J Public Health Nations Health.

[8] Cöster ME, Karlsson M, Ohlsson C (2020). Physical function tests predict incident falls: A prospective study of 2969 men in the Swedish Osteoporotic fractures in men study. Scand J Public Health.

[9] Cooper R, Kuh D, Hardy R (2010). Objectively measured physical capability levels and mortality: systematic review and meta-analysis. BMJ.

[10] Cesari M, Kritchevsky SB, Newman AB (2009). Added value of physical performance measures in predicting adverse health-related events: results from the health, aging and body composition study. J Am Geriatr Soc.

[11] Guralnik JM, Ferrucci L, Simonsick EM (1995). Lower-extremity function in persons over the age of 70 years as a Predictor of subsequent disability. N Engl J Med.

[12] Keevil VL, Cooper AJM, Wijndaele K (2016). Objective sedentary time, moderate-to-vigorous physical activity, and physical capability in a British cohort. Med Sci Sports Exerc.

[13] Fried LP, Tangen CM, Walston J (2001). Frailty in older adults: evidence for a phenotype. J Gerontol A Biol Sci Med Sci.

[14] Cruz-Jentoft AJ, Bahat G, Bauer J (2019). Sarcopenia: revised European consensus on definition and diagnosis. Age Ageing.

[15] Brown RT, Covinsky KE (2020). Moving prevention of functional impairment upstream: is middle age an ideal time for intervention. *Womens Midlife Health*.

[16] Gill TM, Gahbauer EA, Allore HG (2006). Transitions between frailty States among community-living older persons. Arch Intern Med.

[17] Majumder S, Mondal T, Deen MJ (2017). Wearable sensors for remote health monitoring. *Sensors*.

[18] Dias D, Paulo Silva Cunha J (2018). Wearable health devices—vital sign monitoring, systems and Technologies. *Sensors*.

[19] Karas M, Glynn NW, Harris T (2020). Accelerometry data in health research: challenges and opportunities.

[20] Caspersen C, Powell K, Gregory C (1985). Physical activity, exercise, and physical fitness: definitions and distinctions for health-related research. public health Rep. 1985;Mar-Apr100:126-31. US Natl Libr Med.

[21] Dipietro L, Campbell WW, Buchner DM (2019). Physical activity, injurious falls, and physical function in aging: an umbrella review. Med Sci Sports Exerc.

[22] Chase JAD, Phillips LJ, Brown M (2017). Physical activity intervention effects on physical function among community-dwelling older adults: A systematic review and meta-analysis. J Aging Phys Act.

[23] Ramsey KA, Rojer AGM, D’Andrea L (2021). The Association of objectively measured physical activity and sedentary behavior with Skeletal muscle strength and muscle power in older adults: A systematic review and meta-analysis. Ageing Res Rev.

[24] Dekkers OM, Vandenbroucke JP, Cevallos M COSMOS-E: guidance on conducting systematic reviews and meta-analyses of observational studies of etiology. *PLoS Med*.

[25] Higgins J, Green S (2008). Chapter 22: overview of reviews. Cochrane Handbook for systematic reviews of interventions. Cochrane Database Syst Rev.

[26] Page MJ, McKenzie JE, Bossuyt PM (2021). The PRISMA 2020 statement: an updated guideline for reporting systematic reviews. BMJ.

[27] Patrizio E, Calvani R, Marzetti E (2021). Physical functional assessment in older adults. J Frailty Aging.

[28] Beaudart C, Rolland Y, Cruz-Jentoft AJ (2019). Assessment of muscle function and physical performance in daily clinical practice : A position paper endorsed by the european society for clinical and economic aspects of osteoporosis, osteoarthritis and musculoskeletal diseases (esceo). *Calcif Tissue Int*.

[29] Peterson RA, Brown SP (2005). On the use of beta coefficients in meta-analysis. J Appl Psychol.

[30] Nieminen P, Lehtiniemi H, Vähäkangas K (2013). Standardised regression coefficient as an effect size index in Summarising findings in Epidemiological studies. *Ebph*.

[31] Nieminen P (2022). Application of standardized regression coefficient in meta-analysis. *BioMedInformatics*.

[32] Riley RD, Moons KGM, Snell KIE (2019). A guide to systematic review and meta-analysis of Prognostic factor studies. BMJ.

[33] Egger M, Smith GD, Schneider M (1997). Bias in meta-analysis detected by a simple, graphical test. BMJ.

[34] Hsueh M-C, Rutherford R, Chou C-C (2020). Objectively assessed physical activity patterns and physical function in community-dwelling older adults: a cross-sectional study in Taiwan. BMJ Open.

[35] Hsueh M-C, Lin C-Y, Lai T-F (2021). Is achieving 7,000 steps/day cross-Sectionally and prospectively associated with older adults' lower-extremity performance?. BMC Geriatr.

[36] Gobbo LA, Júdice PB, Hetherington-Rauth M (2020). Sedentary patterns are associated with bone mineral density and physical function in older adults: cross-sectional and prospective data. Int J Environ Res Public Health.

[37] Mizumoto A, Ihira H, Makino K (2015). Physical activity during winter in old-old women associated with physical performance after one year: A prospective study. *Curr Gerontol Geriatr Res*.

[38] Yerrakalva D, Hajna S, Wijndaele K (2022). Bidirectional associations of accelerometer-assessed physical activity and sedentary time with physical function among older English adults: the EPIC-Norfolk cohort study. *Eur J Ageing*.

[39] Adachi T, Kono Y, Iwatsu K (2018). Duration of moderate to vigorous daily activity is negatively associated with slow walking speed independently from step counts in elderly women aged 75 years or over: A cross-sectional study. Arch Gerontol Geriatr.

[40] Jantunen H, Wasenius N, Salonen MK (2017). Objectively measured physical activity and physical performance in old age. Age Ageing.

[41] Johansson J, Morseth B, Scott D (2021). Moderate-to-vigorous physical activity modifies the relationship between sedentary time and Sarcopenia: the Tromsø study 2015–2016. J Cachexia Sarcopenia Muscle.

[42] Kim M, Yoshida H, Sasai H (2015). Association between objectively measured sleep quality and physical function among community-dwelling oldest old Japanese: A cross-sectional study. Geriatr Gerontol Int.

[43] Kruger HS, Havemann-Nel L, Ravyse C (2016). Physical activity energy expenditure and Sarcopenia in black South African urban women. J Phys Act Health.

[44] Lai TF, Liao Y, Lin CY (2020). Moderate-to-vigorous physical activity duration is more important than timing for physical function in older adults. *Sci Rep*.

[45] Lerma NL, Cho CC, Swartz AM (2018). Isotemporal substitution of sedentary behavior and physical activity on function. Med Sci Sports Exerc.

[46] Lohne-Seiler H, Kolle E, Anderssen SA (2016). Musculoskeletal fitness and balance in older individuals (65-85 years) and its association with steps per day: a cross sectional study. BMC Geriatr.

[47] Mañas A, Del Pozo-Cruz B, Rodríguez-Gómez I (2019). Dose-response association between physical activity and sedentary time categories on ageing biomarkers. BMC Geriatr.

[48] Meier NF, Lee D (2020). Physical activity and Sarcopenia in older adults. *Aging Clin Exp Res*.

[49] Mendham AE, Goedecke JH, Micklesfield LK (2021). Understanding factors associated with Sarcopenic obesity in older African women from a low-income setting: a cross-sectional analysis. *BMC Geriatr*.

[50] Aggio DA, Sartini C, Papacosta O (2016). Cross-sectional associations of objectively measured physical activity and sedentary time with Sarcopenia and Sarcopenic obesity in older men. *Preventive Medicine*.

[51] Nagai K, Tamaki K, Kusunoki H (2018). Isotemporal substitution of sedentary time with physical activity and its associations with frailty status. Clin Interv Aging.

[52] Oguma Y, Osawa Y, Takayama M (2017). Validation of questionnaire-assessed physical activity in comparison with objective measures using accelerometers and physical performance measures among community-dwelling adults aged ≥85 years in Tokyo, Japan. Journal of Physical Activity and Health.

[53] Osuka Y, Yabushita N, Kim M (2015). Association between habitual light-intensity physical activity and lower-extremity performance: A cross-sectional study of community-dwelling older Japanese adults. Geriatr Gerontol Int.

[54] Pina I, Mendham AE, Tomaz SA Intensity matters for musculoskeletal health: A cross-sectional study on movement behaviors of older adults from high-income Scottish and low-income South African communities. IJERPH.

[55] Reid N, Daly RM, Winkler EAH (2016). Associations of monitor-assessed activity with performance-based physical function. *PLoS ONE*.

[56] Ribeiro A, Verlengia R, de Oliveira MRM (2021). Compliance of the physical activity guidelines accumulated in bouts ≥10 min and Nonbouts and its association with body composition and physical function: A cross-sectional study in Brazilian older adults. J Aging Phys Act.

[57] Sánchez-Sánchez JL, Mañas A, García-García FJ (2019). Sedentary behaviour, physical activity, and Sarcopenia among older adults in the TSHA: Isotemporal substitution model. J Cachexia Sarcopenia Muscle.

[58] Santos DA, Silva AM, Baptista F (2012). Sedentary behavior and physical activity are independently related to functional fitness in older adults. *Experimental Gerontology*.

[59] Savikangas T, Tirkkonen A, Alen M (2020). Associations of physical activity in detailed intensity ranges with body composition and physical function. a cross-sectional study among sedentary older adults. Eur Rev Aging Phys Act.

[60] Schrack JA, Kuo P-L, Wanigatunga AA (2019). Active-to-sedentary behavior transitions, fatigability, and physical functioning in older adults. The Journals of Gerontology.

[61] Aoyagi Y, Park H, Watanabe E (2009). Habitual physical activity and physical fitness in older Japanese adults: the Nakanojo study. Gerontology.

[62] Spartano NL, Lyass A, Larson MG (2019). Objective physical activity and physical performance in middle-aged and older adults. Exp Gerontol.

[63] Thiebaud RS, Abe T, Ogawa M (2020). Accelerometer-determined intensity and duration of habitual physical activity and walking performance in well-functioning middle-aged and older women: A cross-sectional study. J Frailty Aging.

[64] van der Velde JHPM, Savelberg HHCM, van der Berg JD (2017). Sedentary behavior is only marginally associated with physical function in adults aged 40-75 years - the Maastricht study. Front Physiol.

[65] Ward-Ritacco CL, Adrian AL, Johnson MA (2014). Adiposity, physical activity, and muscle quality are independently related to physical function performance in middle-aged postmenopausal women. Menopause.

[66] Ward-Ritacco CL, Meyer A, Walker G (2020). Percent body fat, but not lean mass, is associated with objectively measured physical function in middle-aged women. Maturitas.

[67] Westbury LD, Dodds RM, Syddall HE (2018). Associations between objectively measured physical activity, body composition and Sarcopenia: findings from the Hertfordshire Sarcopenia study (HSS). *Calcif Tissue Int*.

[68] Yamada M, Arai H, Nagai K (2011). Differential determinants of physical daily activities in frail and Nonfrail community-dwelling older adults. J Clin Gerontol Geriatr.

[69] Yasunaga A, Shibata A, Ishii K (2017). Associations of sedentary behavior and physical activity with older adults’ physical function: an Isotemporal substitution approach. BMC Geriatr.

[70] Cooper AJM, Simmons RK, Kuh D (2015). Physical activity, sedentary time and physical capability in early old age: British birth cohort study. *PLoS ONE*.

[71] Cooper R, Stamatakis E, Hamer M (2020). Associations of sitting and physical activity with grip strength and balance in mid-life: 1970 British cohort study. *Scandinavian Med Sci Sports*.

[72] Davis MG, Fox KR, Stathi A (2014). Objectively measured sedentary time and its association with physical function in older adults. J Aging Phys Act.

[73] Duck AA, Stewart MW, Robinson JC (2019). Physical activity and postural balance in rural community dwelling older adults. *Applied Nursing Research*.

[74] Hall KS, Cohen HJ, Pieper CF (2017). Physical performance across the adult life span: correlates with age and physical activity. *GERONA*.

[75] Izawa KP, Shibata A, Ishii K (2017). Associations of low-intensity light physical activity with physical performance in community-dwelling elderly Japanese: A cross-sectional study. *PLoS ONE*.

[76] Troiano RP, Berrigan D, Dodd KW (2008). Physical activity in the United States measured by accelerometer. Med Sci Sports Exerc.

[77] Freedson PS, Melanson E, Sirard J (1998). Calibration of the computer science and applications. *Med Sci Sports Exerc*.

[78] Rojer AGM, Reijnierse EM, Trappenburg MC (2018). Instrumented assessment of physical activity is associated with muscle function but not with muscle mass in a general population. J Aging Health.

[79] Borenstein M, Hedges L, Higgins J (2015). Regression in meta-analysis. C meta-regression man.

[80] Sylvia LG, Bernstein EE, Hubbard JL (2014). Practical guide to measuring physical activity. J Acad Nutr Diet.

[81] Cleland CL, Ferguson S, McCrorie P (2020). Considerations in processing Accelerometry data to explore physical activity and sedentary time in older adults. J Aging Phys Act.

[82] Lee IM, Shiroma EJ (2014). Using accelerometers to measure physical activity in large-scale Epidemiological studies: issues and challenges. Br J Sports Med.

[83] Granat MH (2012). Event-based analysis of free-living behaviour. Physiol Meas.

[84] Shiroma EJ, Lee IM, Schepps MA (2019). Physical activity patterns and mortality: the weekend warrior and activity bouts. Med Sci Sports Exerc.

[85] Wilson JJ, McMullan I, Blackburn NE (2022). The Association of physical activity fragmentation with physical function in older adults: analysis from the SITLESS study. *JAL*.

[86] Dall PM, Skelton DA, Dontje ML (2018). Characteristics of a protocol to collect objective physical activity/sedentary behavior data in a large study: seniors USP (understanding sedentary patterns). *J Meas Phys Behav*.

[87] Atrsaei A, Dadashi F, Hansen C (2020). Postural transitions detection and characterization in healthy and patient populations using a single waist sensor. J Neuroeng Rehabil.

[88] Tomey KM, Sowers MFR (2009). Assessment of physical functioning: A conceptual model encompassing environmental factors and individual compensation strategies. Phys Ther.

[89] Wittink H, Rogers W, Sukiennik A (2003). Physical functioning: self-report and performance measures are related but distinct. Spine.

[90] Fried LP (2016). Interventions for human frailty: physical activity as a model. Cold Spring Harb Perspect Med.

[91] Hillsdon MM, Brunner EJ, Guralnik JM (2005). Prospective study of physical activity and physical function in early old age. Am J Prev Med.

[92] de Carvalho IA, Epping-Jordan J, Beard JR (2019). Integrated care for older people.

