# Supplementary material for: Associations between device-measured physical activity and performance-based physical function outcomes in adults: a systematic review and meta-analysis
Source: BMJ Public Health. 2023 Oct 30;1(1):e100000. doi: 10.1136/bmjph-2023-100000 (PMC11812739; doi:10.1136/bmjph-2023-100000)
Supplement: online supplemental file 1 [file bmjph-1-1-s001.pdf]

Systematic review search strategy:

Associations between physical function and device-based measures of habitual physical activity in mid- and later-life: A systematic review and meta-analysis

General search strategy:

Terms:

1. (physical* adj2 (activ* or inactiv* or behavio* or exercis* or fitness)). ti,ab
2. (sedentary adj2 (activ* or behavio*)). ti,ab
3. (habitual* adj2 (activ* or exercise)). ti,ab
4. (sitting adj2 (time or behavio*)). ti,ab
5. energy expend*. ti,ab
6. exercis*. ti,ab

7. acceleromet*. ti,ab
8. (activity adj2 (monitor* or device*)). ti,ab
9. motion sensor*. ti,ab
10. inclinometer*. ti,ab
11. pedometer*. ti,ab
12. Heart rate. ti,ab

13. (physical* adj2 (function* or capacit* or impair* or abilit* or capabilit*)). ti,ab
14. (function* adj2 (capacit* or limitation* or impair* or status or capabilit*)). ti,ab
15. (speed* adj2 (gait or walk*)). ti,ab
16. (mobility* adj2 (capacit* or limitation* or impair* or status or capabilit*)). ti,ab
17. grip strength. ti,ab
18. balance. ti,ab
19. (transition* adj2 (sit* or stand*)). ti,ab
20. (sit* adj2 stand*). ti,ab
21. timed up and go. ti,ab

22. (observational adj2 (stud* or cohort)). ti,ab
23. (cohort ajd2 (stud* or analy*)). ti,ab
24. (follow up adj2 (stud* or analy*)). ti,ab
25. epidemiolog*. ti,ab
26. prospective. ti,ab
27. cross sectional. ti,ab

28. retrospective. ti,ab<br>29. longitudinal. ti,ab
**<u>Example strategy:</u>**<br>1 or 2 or 3 or 4 or 5 or 6<br>AND<br>7 or 8 or 9 or 10 or 11 or 12<br>AND<br>13 or 14 or 15 or 16 or 17 or 18 or 19 or 20 or 21<br>AND<br>22 or 23 or 24 or 25 or 26 or 27 or 28 or 29

Ovid (PubMed) search:

1.	(physical* adj2 (activ* or inactiv* or behavio* or exercis* or fitness)).ti,ab.
2.	(sedentary adj2 (activ* or behavio*)).ti,ab.
3.	(habitual* adj2 (activ* or exercise)).ti,ab.
4.	(sitting adj2 (time or behavio*)).ti,ab.
5.	energy expend*.ti,ab.
6.	exercis*.ti,ab.
7.	acceleromet*.ti,ab.
8.	(activity adj2 (monitor* or device*)).ti,ab.
9.	motion sensor*.ti,ab.
10.	inclinometer*.ti,ab.
11.	pedometer*.ti,ab.
12.	Heart rate.ti,ab.
13.	(physical* adj2 (function* or capacit* or impair* or abilit* or capabilit*)).ti,ab.
14.	(function* adj2 (capacit* or limitation* or impair* or status or capabilit*)).ti,ab.
15.	(speed* adj2 (gait or walk*)).ti,ab.
16.	(mobility* adj2 (capacit* or limitation* or impair* or status or capabilit*)).ti,ab.
17.	grip strength.ti,ab.
18.	balance.ti,ab.
19.	(transition* adj2 (sit* or stand*)).ti,ab.
20.	(sit* adj2 stand*).ti,ab.
21.	timed up.mp. and go.ti,ab. [mp=ti, bt, ab, ot, nm, hw, fx, kf, ox, px, rx, ui, sy, tn, dm, mf, dv, dq]
22.	(observational adj2 (stud* or cohort)).ti,ab.
23.	(cohort adj2 (stud* or analy*)).ti,ab.
24.	(follow up adj2 (stud* or analy*)).ti,ab.
25.	epidemiolog*.ti,ab.
26.	prospective.ti,ab.

27. cross sectional.ti,ab.
28. retrospective.ti,ab.
29. longitudinal.ti,ab.
30. 1 or 2 or 3 or 4 or 5 or 6
31. 7 or 8 or 9 or 10 or 11 or 12
32. 13 or 14 or 15 or 16 or 17 or 18 or 19 or 20 or 21
33. 22 or 23 or 24 or 25 or 26 or 27 or 28 or 29
34. 30 and 31 and 32 and 33

Web of Science search:

#9 AND #19 AND #26 AND #33

#1 OR #2 OR #3 OR #4 OR #5 OR #6 OR #7 OR #8
TS=longitudinal
TS=retrospective
TS=cross sectional
TS=prospective
TS=epidemiolog*
TS=(follow up NEAR/2 (stud* or analy*))
TS=(cohort NEAR/2 (stud* or analy*))
TS=(observational NEAR/2 (stud* or cohort))

#10 OR #11 OR #12 OR #13 OR #14 OR #15 OR #16 OR #17 OR #18
TS=timed up "and" go
TS=(sit* NEAR/2 stand*)
TS=(transition* NEAR/2 (sit* or stand*))
TS=balance
TS=grip strength
TS=(mobility* NEAR/2 (capacit* or limitation* or impair* or status or capabilit*))
TS=(speed* NEAR/2 (gait or walk*))
TS=(function* NEAR/2 (capacit* or limitation* or impair* or status or capabilit*))
TS=(physical* NEAR/2 (function* or capacit* or impair* or abilit* or capabilit*))

#20 OR #21 OR #22 OR #23 OR #24 OR #25
TS=heart rate
TS=pedometer*
TS=inclinometer*
TS=motion sensor*
TS=(activity NEAR/2 (monitor* or device*))
TS=acceleromet*

#27 OR #28 OR #29 OR #30 OR #31 OR #32
TS=exercis*
TS=energy expend*
TS=(sitting NEAR/2 (time or behavio*))
TS=(habitual* NEAR/2 (activ* or exercise))
TS=(sedentary NEAR/2 (activ* or behavio*))
TS=(physical* NEAR/2 (activ* or inactiv* or behavio* or exercis* or fitness))

EBSCOhost

#	Query	Limiters/Expanders	Last Run Via
S68	S64 AND S65 AND S66 AND S67	Expanders - Apply equivalent subjects Search modes - Boolean/Phrase	Interface - EBSCOhost Research Databases Search Screen - Advanced Search Database - CINAHL Complete;SPORTDiscus
S67	S56 OR S57 OR S58 OR S59 OR S60 OR S61 OR S62 OR S63	Expanders - Apply equivalent subjects Search modes - Boolean/Phrase	Interface - EBSCOhost Research Databases Search Screen - Advanced Search Database - CINAHL Complete
S66	S47 OR S48 OR S49 OR S50 OR S51 OR S52 OR S53 OR S54 OR S55	Expanders - Apply equivalent subjects Search modes - Boolean/Phrase	Interface - EBSCOhost Research Databases Search Screen - Advanced Search Database - CINAHL Complete
S65	S41 OR S42 OR S43 OR S44 OR S45 OR S46	Expanders - Apply equivalent subjects Search modes - Boolean/Phrase	Interface - EBSCOhost Research Databases Search Screen - Advanced Search Database - CINAHL Complete
S64	S35 OR S36 OR S37 OR S38 OR S39 OR S40	Expanders - Apply equivalent subjects Search modes - Boolean/Phrase	Interface - EBSCOhost Research Databases Search Screen - Advanced Search Database - CINAHL Complete
S63	TI longitudinal OR AB longitudinal	Expanders - Apply equivalent subjects Search modes - Boolean/Phrase	Interface - EBSCOhost Research Databases Search Screen - Advanced Search Database - CINAHL Complete
S62	TI retrospective OR AB retrospective	Expanders - Apply equivalent subjects Search modes - Boolean/Phrase	Interface - EBSCOhost Research Databases Search Screen - Advanced Search Database - CINAHL Complete
S61	TI cross sectional OR AB cross sectional	Expanders - Apply equivalent subjects Search modes - Boolean/Phrase	Interface - EBSCOhost Research Databases Search Screen - Advanced Search Database - CINAHL Complete
S60	TI prospective OR AB prospective	Expanders - Apply equivalent subjects Search modes - Boolean/Phrase	Interface - EBSCOhost Research Databases Search Screen - Advanced Search Database - CINAHL Complete
S59	TI epidemiolog* OR AB epidemiolog*	Expanders - Apply equivalent subjects Search modes - Boolean/Phrase	Interface - EBSCOhost Research Databases Search Screen - Advanced Search Database - CINAHL Complete
S58	TI ((follow up N2 (stud* or analy*))) OR AB ((follow up N2 (stud* or analy*)))	Expanders - Apply equivalent subjects Search modes - Boolean/Phrase	Interface - EBSCOhost Research Databases Search Screen - Advanced Search Database - CINAHL Complete
S57	TI ((cohort N2 (stud* or analy*))) OR AB ((cohort N2 (stud* or analy*)))	Expanders - Apply equivalent subjects Search modes - Boolean/Phrase	Interface - EBSCOhost Research Databases Search Screen - Advanced Search Database - CINAHL Complete
S56	TI ((observational N2 (stud* or cohort))) OR AB ((observational N2 (stud* or cohort)))	Expanders - Apply equivalent subjects Search modes - Boolean/Phrase	Interface - EBSCOhost Research Databases Search Screen - Advanced Search Database - CINAHL Complete

S55	TI (timed up and go) OR AB (timed up and go)	Expanders - Apply equivalent subjects Search modes - Boolean/Phrase	Interface - EBSCOhost Research Databases Search Screen - Advanced Search Database - CINAHL Complete
S54	TI (sit* N2 stand*) OR AB (sit* N2 stand*)	Expanders - Apply equivalent subjects Search modes - Boolean/Phrase	Interface - EBSCOhost Research Databases Search Screen - Advanced Search Database - CINAHL Complete
S53	TI ((transition* N2 (sit* or stand*))) OR AB ((transition* N2 (sit* or stand*)))	Expanders - Apply equivalent subjects Search modes - Boolean/Phrase	Interface - EBSCOhost Research Databases Search Screen - Advanced Search Database - CINAHL Complete
S52	TI balance OR AB balance	Expanders - Apply equivalent subjects Search modes - Boolean/Phrase	Interface - EBSCOhost Research Databases Search Screen - Advanced Search Database - CINAHL Complete
S51	TI grip strength OR AB grip strength	Expanders - Apply equivalent subjects Search modes - Boolean/Phrase	Interface - EBSCOhost Research Databases Search Screen - Advanced Search Database - CINAHL Complete
S50	TI ((mobility* N2 (capacit* or limitation* or impair* or status or capabilit*))) OR AB ((mobility* N2 (capacit* or limitation* or impair* or status or capabilit*)))	Expanders - Apply equivalent subjects Search modes - Boolean/Phrase	Interface - EBSCOhost Research Databases Search Screen - Advanced Search Database - CINAHL Complete
S49	TI ((speed* N2 (gait or walk*))) OR AB ((speed* N2 (gait or walk*)))	Expanders - Apply equivalent subjects Search modes - Boolean/Phrase	Interface - EBSCOhost Research Databases Search Screen - Advanced Search Database - CINAHL Complete
S48	TI ((function* N2 (capacit* or limitation* or impair* or status or capabilit*))) OR AB ((function* N2 (capacit* or limitation* or impair* or status or capabilit*)))	Expanders - Apply equivalent subjects Search modes - Boolean/Phrase	Interface - EBSCOhost Research Databases Search Screen - Advanced Search Database - CINAHL Complete
S47	TI ((physical* N2 (function* or capacit* or impair* or abilit* or capabilit*))) OR AB ((physical* N2 (function* or capacit* or impair* or abilit* or capabilit*)))	Expanders - Apply equivalent subjects Search modes - Boolean/Phrase	Interface - EBSCOhost Research Databases Search Screen - Advanced Search Database - CINAHL Complete
S46	TI heart rate OR AB heart rate	Expanders - Apply equivalent subjects Search modes - Boolean/Phrase	Interface - EBSCOhost Research Databases Search Screen - Advanced Search Database - CINAHL Complete
S45	TI pedometer* OR AB pedometer*	Expanders - Apply equivalent subjects Search modes - Boolean/Phrase	Interface - EBSCOhost Research Databases Search Screen - Advanced Search Database - CINAHL Complete
S44	TI inclinometer* OR AB inclinometer*	Expanders - Apply equivalent subjects Search modes - Boolean/Phrase	Interface - EBSCOhost Research Databases Search Screen - Advanced Search Database - CINAHL Complete
S43	TI motion sensor* OR AB motion sensor*	Expanders - Apply equivalent subjects Search modes - Boolean/Phrase	Interface - EBSCOhost Research Databases Search Screen - Advanced Search Database - CINAHL Complete

S42	TI ((activity N2 (monitor* or device*))) OR AB ((activity N2 (monitor* or device*)))	Expanders - Apply equivalent subjects<br>Search modes - Boolean/Phrase	Interface - EBSCOhost Research Databases<br>Search Screen - Advanced Search<br>Database - CINAHL Complete
S41	TI acceleromet* OR AB acceleromet*	Expanders - Apply equivalent subjects<br>Search modes - Boolean/Phrase	Interface - EBSCOhost Research Databases<br>Search Screen - Advanced Search<br>Database - CINAHL Complete
S40	TI exercis* OR AB exercis*	Expanders - Apply equivalent subjects<br>Search modes - Boolean/Phrase	Interface - EBSCOhost Research Databases<br>Search Screen - Advanced Search<br>Database - CINAHL Complete
S39	TI energy expend* OR AB energy expend*	Expanders - Apply equivalent subjects<br>Search modes - Boolean/Phrase	Interface - EBSCOhost Research Databases<br>Search Screen - Advanced Search<br>Database - CINAHL Complete
S38	TI ((sitting N2 (time or behavio*))) OR AB ((sitting N2 (time or behavio*)))	Expanders - Apply equivalent subjects<br>Search modes - Boolean/Phrase	Interface - EBSCOhost Research Databases<br>Search Screen - Advanced Search<br>Database - CINAHL Complete
S37	TI ((habitual* N2 (activ* or exercise))) OR AB ((habitual* N2 (activ* or exercise)))	Expanders - Apply equivalent subjects<br>Search modes - Boolean/Phrase	Interface - EBSCOhost Research Databases<br>Search Screen - Advanced Search<br>Database - CINAHL Complete
S36	TI ((sedentary N2 (activ* or behavio*))) OR AB ((sedentary N2 (activ* or behavio*)))	Expanders - Apply equivalent subjects<br>Search modes - Boolean/Phrase	Interface - EBSCOhost Research Databases<br>Search Screen - Advanced Search<br>Database - CINAHL Complete
S35	TI ((physical* N2 (activ* or inactiv* or behavio* or exercis* or fitness))) OR AB ((physical* N2 (activ* or inactiv* or behavio* or exercis* or fitness)))	Expanders - Apply equivalent subjects<br>Search modes - Boolean/Phrase	Interface - EBSCOhost Research Databases<br>Search Screen - Advanced Search<br>Database - CINAHL Complete
