# Supplementary material for: Associations between device-measured physical activity and performance-based physical function outcomes in adults: a systematic review and meta-analysis
Source: BMJ Public Health. 2023 Oct 30;1(1):e100000. doi: 10.1136/bmjph-2023-100000 (PMC11812739; doi:10.1136/bmjph-2023-100000)
Supplement: online supplemental file 2 [file bmjph-1-1-s002.pdf]

Adapted version of The National Institutes of Health (NIH) quality assessment tool for observational cohort and cross-sectional studies

Website: https://www.nhlbi.nih.gov/health-topics/study-quality-assessment-tools

**Major Components**	**Response options**		
1. Was the research question or objective in this paper clearly stated?	Yes	No	Not Applicable/ Not Reported
2. Was the study population clearly specified and defined?	Yes	No	Not Applicable/ Not Reported
3. Was the participation rate of eligible persons at least 50%?	Yes	No	Not Applicable/ Not Reported
4. Were all the subjects selected or recruited from the same or similar populations (including the same time period)? Were inclusion and exclusion criteria for being in the study prespecified and applied uniformly to all participants?	Yes	No	Not Applicable/ Not Reported
5. Was a sample size justification, power description, or variance and effect estimates provided?	Yes	No	Not Applicable/ Not Reported
6. For the analyses in this paper, were the exposure(s) of interest measured prior to the outcome(s) being measured?	Yes	No	Not Applicable/ Not Reported
7. Was the timeframe sufficient so that one could reasonably expect to see an association between exposure and outcome if it existed?	Yes	No	Not Applicable/ Not Reported
8. For exposures that can vary in amount or level, did the study examine different levels of the exposure as related to the outcome (e.g., categories of exposure, or exposure measured as continuous variable)?	Yes	No	Not Applicable/ Not Reported
9. Were the exposure measures (independent variables) clearly defined, valid, reliable, and implemented consistently across all study participants?	Yes	No	Not Applicable/ Not Reported
10. Was the accelerometer protocol reported up to the standards of the Montoye et al. (2018) guidelines? Reporting of; brand, epoch, placement, days, valid hours/days, non-wear criteria, accelerometer outcomes and interpretation (e.g. MVPA and cut points used)	Yes	No	Not Applicable/ Not Reported
11. Was the exposure(s) assessed more than once over time?	Yes	No	Not Applicable/ Not Reported
12. Were the outcome measures (dependent variables) clearly defined, valid, reliable, and implemented consistently across all study participants?	Yes	No	Not Applicable/ Not Reported
13. Were the outcome assessors blinded to the exposure status of participants?	Yes	No	Not Applicable/ Not Reported
14. Was loss to follow-up after baseline 20% or less?	Yes	No	Not Applicable/ Not Reported
15. Were key potential confounding variables measured and adjusted statistically for their impact on the relationship between exposure(s) and outcome(s) – with age and sex the minimum?	Yes	No	Not Applicable/ Not Reported
