# Supplementary material for: Associations between device-measured physical activity and performance-based physical function outcomes in adults: a systematic review and meta-analysis
Source: BMJ Public Health. 2023 Oct 30;1(1):e100000. doi: 10.1136/bmjph-2023-100000 (PMC11812739; doi:10.1136/bmjph-2023-100000)
Supplement: online supplemental file 3 [file bmjph-1-1-s003.pdf]

Table 2. Ascertainment and measurement characteristics of device-measured physical activity.

Author, Year	Device	Name/Brand	Placement	Wear days (N)	Valid day definition (h/day)	Valid days required	Wear time mean (SD) or *median [IQR]* (h/day)	PA exposure measure/s	Units	Cut-off values/definition	Mean (SD) or *median [IQR]*
Adachi 2018	A/P	Kenz Lifecorder	Waist	7	N/R	N/R	N/R	Step count	Steps/day	Device detected	6523 (2990)
								MVPA	Min/day	Device detected	17.1 (16.6)
Aggio 2016*	A	Actigraph GT3X	Hip	7	≥10	3	*Non-sarcopenia: 854.8 [850.8, 858.8], Sarcopenia: 848.4 [838.3, 858.5], Severe sarcopenia: 839.5 [821.1, 857.9]*	LPA	Min/day	100-1040 CPM	*Non-sarcopenia: 201.9 [198.1, 205.6], Sarcopenia: 196.4 [187.1, 205.7], Severe sarcopenia: 169.2 [152.5, 185.9]*
								MVPA	Min/day	>1040 CPM	*Non-sarcopenia: 42.1 [40.1, 44.0], Sarcopenia: 37.9 [32.8, 43.1], Severe sarcopenia: 19.8 [14.4, 25.1]*
Aoyagi 2009	A/P	Kenz Lifecorder	Waist	1-year	N/R	N/R	N/R	Step count	Steps/day	Device detected	6574 (2715)
								TPA (PA)	Min/day	≥3 METs	17.3 (11.9)
Cooper 2015	HR+A	CamNtech Actiheart	Chest	5	N/R	≥2	N/R	TPA (PAEE)	Min/day	Device detected	*M:* 38.1 (15.7), *F:* 34.2 (13.3)
								MVPA	kJ/kg/day	≥3 METs	*M:* 90.5 (64.9), *F:* 79.9 (54.9)
Cooper 2020	A	activPAL3 micro	Thigh	7	≥10	1	*M:* 16 (1.3), *F:* 15.7 (1.3)	TPA	Hour/day	Device detected	*M:* 2.0 (0.7), *F:* 2.0 (0.7)
								MVPA	Hour/day	≥100 step cadence threshold	*M:* 0.8 (0.4), *F:* 0.8 (0.4)
Davis 2014	A	ActiGraph GT1M	Waist	7	≥10	5	14.4 (1.4)	MVPA	Min/registered hour	>1951 CPM	0.9 (1.3)
Duck 2019	A	ActiGraph GT3X	Waist/Hip	7	≥10	4	N/R	LPA	Min/day	100-1951 CPM	114.17 (55.91)
								MPA	Min/day	1952-5724 CPM	10.88 (11.91)
								VPA	Min/day	N/R	0.52 (2.80)
								MVPA	Min/day	≥1952 CPM	11.40 (13.11)
Gobbo 2020	A	ActiGraph GT3X	Waist	5	≥10	3 (inc. 1 w/e day)	N/R	MVPA	Min/day	≥2020 CPM	21.1 (22.5)
Hall 2017	A	ActiGraph GT3X and GT3X+	Waist	7	≥10	4	14 (N/R)	Step count	Sum of steps	Device detected	N/R
								LPA	% of total wear time in LPA	Device detected	N/R
								MVPA	%of total wear time in MVPA	Device detected	N/R
Hsueh 2020	A	ActiGraph GT3X	Waist	7	≥10	4 (inc. 1 w/e day)	*M*: 905.7 (109.5), *F:* 925.3 (73.2) min/day	Step count	Steps/day	Device detected	*M:* 8408 (4051.7), *F:* 7079.0 (3034.2)

Author, Year	Device	Name/Brand	Placement	Wear days (N)	Valid day definition (h/day)	Valid days required	Wear time mean (SD) or *median [IQR]* (h/day)	PA exposure measure/s	Units	Cut-off values/definition	Mean (SD) or *median [IQR]*
								TPA	Min/day	≥100 CPM	*M:* 292.0 (90.3), *F:* 326.4 (79.6)
								MVPA	Min/day	≥2020 CPM	*M:* 36.7 (27.8), *F:* 19.5 (19.1)
Izawa 2017	A	Omron HJA-750C	N/R	7	≥10	4 (inc. 1 w/e day)	N/R	MVPA (MPA)	Min/day	≥3 METs	*M:* 50.9 (37.6), *F:* 48.1 (27.3)
Jantunen 2017		Sense-Wear Pro 3 Armband	Upper Arm	10	N/R	5 (inc. 1 w/e day)	1436.8 (6.0) min/day	TPA	MET min/day	>1.5 METs	1779.6 (298.5)
								LPA	MET min/day	>1.5 to <3.0 METs	496.7 (181.6)
								MVPA	MET min/day	>3.0 METs	295.6 (230.0)
Johansson 2021	A	ActiGraph wGT3X-BT	Hip	8	≥10	4	*M:* 116.9 (17.1), *F:* 116.6 (15.6) total hours	LPA	Min/day	150-2698 CPM	*M:* 380.8(87.9), *F:* 415.6(87.3)
								MVPA	Min/day	≥2690 CPM	*M:* 41.1(32.5), *F:* 34.8(26.6)
Kim 2015	A	ActiGraph GT3X+	Wrist	8	N/R	5	N/R	TPA (PA)	counts/min/day	Mean count per minute of vector magnitude (daily total counts divided by valid wear-time)	1771.8 (520.6)
Kruger 2016	HR+A	CamNtech ActiHeart	N/R	7	N/R	4	6.97 day/week	TPA (PAEE)	kJ	Device detected	4893 (3763)
Lai 2020	A	ActiGraph GT3X+	Waist	7	≥10	4	15.4 (1.4)	MVPA	Min/day	≥2020 CPM	25.0 (26.2)
Lerma 2018	A	ActiGraph GT3X+	Hip	7	N/R	N/R	13.99 (0.13)	LPA	Min/day	100-1951 CPM	283.1 (73.3)
								MVPA	Min/day	≥1952 CPM	25.0 (20.9)
Lohne-Seiler 2016	A	ActiGraph GT1M	Hip	7	≥10	1	14.0 (1.2) h/day	Step count	Steps/day	N/R	*M:* 7356, *F:* 7551
Manas 2019*	A	ActiGraph GT3X and ActiTrainer	Hip	7	≥8	4	786.0 (82.6) min/day	LPA	Min/day	100-1951 CPM	226.8 (86.2)
								MVPA	Min/day	≥1952 CPM	19.4 (23.8)
Meier 2020	P	Omron HJ-321	Waist	7	N/R	N/R	94.4% had complete data	Step count	Steps/day	Device detected	4943(2632)
Mendham 2021	A	Actigraph GTX3+ and ActivPAL	Waist + Thigh	7	≥10	4	N/R	TPA	Min/day	≥100 CPM	N/R
								LPA	Min/day	100-2019 CPM	326.2 (91.0)
								MVPA	Min/day	≥2020 CPM	*9.1 [2.3, 15.9]*
Mizumoto 2015*	A/P	Kenz Lifecorder GS	Buttock	1-week	N/R	N/R	N/R	Step count	Steps/day	N/R	*Baseline:* 4244.0 (2683.3), *Follow up:* 4809.8 (3116.3)

Author, Year	Device	Name/Brand	Placement	Wear days (N)	Valid day definition (h/day)	Valid days required	Wear time mean (SD) or *median [IQR]* (h/day)	PA exposure measure/s	Units	Cut-off values/definition	Mean (SD) or *median [IQR]*
								MVPA	Mins/day	N/R	*Baseline:* 8.7 (12.1), *Follow up:* 8.2 (9.6)
Nagai 2018*	A	TDK ActiBand	Wrist	14	≥10	4	1015 (74) min/day	LPA	Min/day	≥1.5 to <3 METs	463 (150)
								MVPA	Min/day	≥3 METs	42 (34)
Oguma 2017*	A	Kenz Lifecorder EX	Waist	7	≥10	4	N/R	Step count	Steps/day	Device detected	*2691 [1607-4423]*
								TPA (PA Index)	METh/week	Equation reported in paper	*2.6 [0.6-69]*
Osuka 2015	A	Kenz Lifecorder	Hip	7	≥10	5	875.3 (92.4) min/day	LPA	Min/day	Device detected 1.8-2.9 METs	57.1 (22.7)
								MVPA	Min/day	Device detected ≥3.6 METs	17.6 (15.3)
Pina 2021	A	Actigraph GT3X+	Hip	7	≥10	4	*Scot:* 913 (46), *SA:* 878 (80) min/day	TPA	Min/day	≥100 CPM	*Scot:* 324 (64), *SA:* 334 (96)
								LPA	Min/day	100-2019 CPM	*Scot: 287* (55), *SA:* 318 (92)
								MVPA	Min/day	≥2020 CPM	*Scot: 27 [15-44], SA: 11 [3-21]*
Reid 2016*	A	ActivPAL3	Thigh	7	≥10 + ≥80% of waking hours	N/R	15.7 (1.1)	Step count (all stepping)	Hour/day	Device detected	2.0 (0.6)
								LPA (stepping)	Hour/day	Device detected	1.0 (0.4)
								MVPA (stepping)	Hour/day	Device detected	1.0 (0.4)
								Sit-to-stand transitions	Transitions/day	Device detected	53.3 (14.8)
Ribeiro 2020	A	ActiGraph GT3X+	Hip	7	≥10	7	*1058.6 [1000.1-1125.7]* min/day	MVPA	Min/day min/day	Freedson	*16.1 [6.7-25.1]*
Rojer 2018	A	DynaPort MoveMonitor	Lower back	7	≥18	4	6.9 days	TPA	Min/day	N/R	271.6 (64.5)
								Step count	Steps/day	Device detected	8608.1 (2961.8)
Sanchez-Sanchez 2019	A	ActiGraph ActiTrainer	Hip	7	≥8	4	84.39 (16.03) total hours	TPA	Counts/day	≥1.5 METs	409365.6 (180677.0)
								LPA	Hour/day	1.5-2.99 METs	5.01 (1.5)
								MVPA	Hour/day	≥3 METs	1.02 (0.78)
Santos 2012	A	Actigraph GT1M	Hip	4	≥10	3 (inc 1 w/e day)	819.6 (87.5) min/day	TPA	Min/day	≥100 CPM	239.7 (100.5)
								LPA	Min/day	100-2019 CPM	213.8 (88.7)
								MVPA	Min/day	≥2020 CPM	26.0 (24.1)
Savikangas 2020	A	UKK RM42	Waist	7	≥10	3	14.1 (1.3)	LPA	Min/day	≥0.0167 to <0.091g	210.3 (66.3)
								MVPA	Min/day	≥0.091g	32.5 (20.1)
Schrack 2019	A	CamNtech Actiheart	Chest	7	95% of data	3	N/R	TPA	Total log activity counts	Device detected	*Low ASTP:* 53009.04 (25578.54), *Mid ASTP:*

Author, Year	Device	Name/Brand	Placement	Wear days (N)	Valid day definition (h/day)	Valid days required	Wear time mean (SD) or *median [IQR]* (h/day)	PA exposure measure/s	Units	Cut-off values/definition	Mean (SD) or *median [IQR]*
											35114.73 (13698.46), *High ASTP:* 21675.93 (11309.85)
Spartano 2019	A	Actical (model no. 198-0200-00)	Hip	8	≥10	4	749 (71) mins/d	Step count	Steps/day	Device detected	6927 (3678)
								MVPA	Min/day	>1486 CPM	19 (22)
Thiebaud 2020*	A	Lifecorder EX	Hip	30	>12	N/R	N/R	LPA	Min/day	<3 METs	60.1 (18.9)
								MPA	Min/day	3-6 METs	21.2 (14.0)
								VPA	Min/day	>6 METs	1.9 (2.0)
van der Velde 2017	A	ActivPAL3	Thigh	8	≥10	1	15.7 (0.9)	TPA	Hour/day	Device detected	2.0 (0.7)
								High intensity PA	Min/day	Device detected (≥110 step/min)	*19.2 [9.6-32.0]*
Ward-Ritacco 2014	A	New Lifestyles-1000	Hip	7 to 10	≥10	4	N/R	Step count	Steps/day	Device detected	9076.2 (3822)
								MVPA	Min/day	≥3.6 METs	30.0 (20.8)
Ward-Ritacco 2020	A	ActiGraph GT9X	Hip	7 to 10	≥10	4	N/R	Step count	Steps/day	Device detected	7711 (2838)
Westbury 2018	A	GENEActiv	Wrist	7	N/R	7	N/R	TPA	Min/day	≥40mg	*M: 137.8 [81.7, 217.2], F: 186.0 [122.1, 240.4]*
								MVPA	Min/day	≥100mg	*M: 14.3 [1.8, 30.2], F: 9.5 [2.1, 18.6]*
Yamada 2011*	P	Yamax PowerWalker EX-510	Pocket	14	N/R	N/R	N/R	Step count	Steps/day	TPA ≥40	4414.4 (2726.3)
Yasunaga 2017	A	Omron HJA-350IT	Waist	7	≥10	4 (inc. 1 w/e day)	901.1 (87.5) min/day	LPA	Min/day	>1.5 to <3.0METs	328.7 (101.4)
								MVPA	Min/day	≥3METs	50.2 (33.5)
Yerrakalva 2022	A	*Baseline:* ActiGraph GT1M *Follow-up:* GT3X	Hip	7	≥10	4	N/R	TPA	Min/day	≥100cpm	251 (117)
								LPA	Min/day	100-808cpm	224.9 (56.5)
								MVPA	Min/day	≥809cpm	77.4 (46.3)

*Asterisk denotes not included in meta-analyses, N/A = not applicable, N/R = not reported, A = accelerometer, P = pedometer, HR= heart rate, PA = physical activity LPA = light intensity physical activity, MVPA = moderate-to-vigorous physical activity, Steps = average or total step count, TPA = total physical activity, MET = metabolic equivalent of task, PAEE = physical activity energy expenditure, kJ = kilojoule, CPM = counts per minute

Table 3. Ascertainment and measurement characteristics of performance-based physical function outcomes.

Author, Year	Measure	Device	Definition and protocol	Units	Mean (SD)
Adachi 2018	Gait	N/A	Usual gait over 10-m; faster of 2 attempts. Slow Gait <1.0m/s	n	*N*=41 (13.3%)
Aggio 2016*	Gait	N/A	Gait over 3-m	Meters/s	*Non-sarcopenia:* 0.95 (0.2), *Sarcopenia:* 0.82 (0.2), *Severe sarcopenia:* 0.62 (0.1)
	HGS	Jamar hydraulic dynamometer	3 attempts with each hand, max used	kg	*Non-sarcopenia:*32.3 (9.9), *Sarcopenia:*28.7 (10.1), *Severe sarcopenia:* 22.2 (6.1)
Aoyagi 2009	Gait	GaitScan8000 Pressure sensors	Usual gait over 5-m	Meters/s	1.43 (0.22)
	HGS	Smedley dynamometer	2 attempts with dominant hand, max used	Newtons	262 (83)
	Balance	Force platform (G-5500)	Stand eyes-open 30s, then closed 30s. Total movement of CoG in horizontal axis was measured over 30s (body sway)	Meters	*Eyes open:* .45 (.17), *Eyes closed:* .94 (.39)
Cooper 2015	HGS	Nottingham electronic dynamometer	3 attempts with each hand, max used	kg	*M:* 46.4 (11.5), *F:* 27.0 (7.5)
	Chair rise	N/A	Time to complete 10 chair rises.	Stands/min	*M:* 26.2 (7.3), *F:* 24.9 (7.3)
	TUG	N/A	Time taken to rise from a chair, walk 3-m, return, and sit back down	Meters/s	*M:* 0.7 (0.2), *F:* 0.7 (0.1)
	Balance	N/A	Time (up to max of 30s) participant could maintain one-legged stand eyes closed	ln/s	*M:* 1.6 (0.6), *F:* 1.6 (0.5)
Cooper 2020	HGS	Smedley dynamometer	Up to 3 attempts with each hand, max used	kg	*M:* 48.2 (8.8), *F:* 29.9 (5.6)
Davis 2014	Gait	N/A	Usual gait over 3 or 4 -m	Score (0-4)	3.5 (0.8)
	Chair rise	N/A	Time to complete 5 chair rises	Score (0-4)	2.7 (1.3)
	Balance	N/A	Ability to maintain tandem, semi, and side-by-side stance for 10s	Score (0-4)	3.6 (0.8)
Duck 2019	TUG	N/A	Time taken to rise from a chair, walk 10-m, return, and sit back down	Score	9.11 (2.93)
	Balance	Berg Balance Scale	14-item instrument, with each item rated 0 (poor balance) to 4 (better balance)	Seconds	50.35 (6.05)
Gobbo 2020	Gait	N/A	2 attempts at gait over 4-m, max used	Meters/s	1.0 (0.2)
	HGS	Camry digital dynamometer model EH101	2 attempts with dominant hand, max used	kg	26.2 (8.2)
	TUG	N/A	Time taken to rise from a chair, walk 3-m, return, and sit back down	Seconds	9.6 (2.4)
Hall 2017	Gait	N/A	2 attempts at gait over 4-m, max used	Meters/s	†
	Chair rise	N/A	No. of chair rises completed in 30-s	n	†
	Walk	N/A	*6MWT:* Distance covered in 6-min walking	Yards	†
	Balance	N/A	Duration of single-leg stance, eyes-open (up to 60s)	Seconds	†
Hsueh 2020	Gait	N/A	Gait over 11-m (central 5-m used)	Seconds	*M:* 2.89 (1.08) *F:* 3.11 (0.71)
	HGS	Jamar Plus+ digital dynamometer	2 attempts with both hands, max used	kg	*M:* 33.3 (6.5) *F:* 21.4 (3.5)

Author, Year	Measure	Device	Definition and protocol	Units	Mean (SD)
	Chair rise	N/A	Time taken to complete 5 chair rises	Seconds	*M:* 7.54 (2.16) *F:* 7.45 (2.70)
	TUG	N/A	Time taken to rise from a chair, walk 3-m, return, and sit back down	Seconds	*M:* 7.13 (2.90) *F:* 7.20 (1.82)
	Balance	N/A	Duration of single leg stance (up to 60s), eyes open, 2 attempts	Seconds	*M:* 39.6 (23.8) *F:* 34.8 (23.0)
Izawa 2017	Gait	N/A	2 attempts at gait over 5-m, max used	Meters/s	*M:*1.8 (0.3), *F:* 1.7 (0.3)
	TUG	N/A	Time taken to rise from a chair, walk 3-m, return, and sit back down	Seconds	*M:* 6.1 (1.2), *F:* 6.5 (1.4)
	Balance	N/A	Duration of single leg stance (up to 60s), eyes open, 2 attempts	Seconds	*M:* 41.8 (21.6), *F:* 44.2 (22.1)
Jantunen 2017	Chair rise	N/A	No. of chair rises completed in 30-s	n	11.5 (2.3)
	Walk	N/A	*6MWT:* Distance covered in 6-min walking	Meters	584.8 (103.6)
	SFT	N/A	Senior Fitness test battery, composite score of 5 tests	Score	46.4 (17.5)
Johansson 2021	HGS	Jamar Plus+ Digital hand Dynamometer	3 attempts with each hand, max used	kg	N/R
	Chair rise	N/A	Time taken to complete 5 chair rises, two attempts, max used	Seconds	N/R
Kim 2015	Gait	N/A	Gait over 11-m (central 5-m used)	Meters/s	1.20 (0.25)
	HGS	Smedley dynamometer	3 attempts, max used	kg	23.4 (7.5)
Kruger 2016	Gait	N/A	Gait over 6-m	Meters/s	1.36 (0.33)
	HGS	Jamar dynamometer	3 attempts with dominant hand, max used	kg	20.4 (6.7)
Lai 2020	Gait	N/A	Gait over 11-m (central 5-m used)	Seconds	N/R
	HGS	Jamar Plus+ dynamometer	3 attempts with both hands, max used	kg	N/R
	Chair rise	N/A	Time taken to complete 5 chair rises, two attempts, max used, two attempts, fastest used	Seconds	N/R
	TUG	N/A	Time taken to rise from a chair, walk 3-m, return, and sit back down, 2 attempts, max used	Seconds	N/R
Lerma 2018	Gait	N/A	2 attempts at gait, faster used	Meters/s	1.1 (0.3)
	Chair rise	N/A	Time taken to complete 5 chair rises, two attempts, max used, two attempts, fastest used	Seconds	15.2 (4.8)
	Walk	N/A	*400mWT:* Time taken to walk 400-m	Meters/s	1.4 (0.3)
	SPPB	N/A	SPPB	Score	9.8 (1.6)
Lohne-Seiler 2016	HGS	Chattanooga dynamometer	3 attempts with dominant hand, max used	kg	*Mean {95%CIs}:* 33.5 {32.3, 34.8}
	Balance	N/A	Duration of single leg stance, eyes open	Seconds	*Mean {95%CIs}:* 19.5 {16.7, 22.2}
Manas 2019*	SPPB	N/A	SPPB	Score	8.4 (3.2)
Meier 2020	Gait	N/A	Gait over 4-m	Meters/s	1.1(0.2)
	HGS	Jamar Plus+ dynamometer	3 attempts with each hand, max used	kg	29.9(10.3)
Mendham 2021	Gait	N/A	Gait over 12-m (central 10-m used)	Meters/s	1.5 (0.3)
	HGS	T.K.K. 5401, Grip-D, Takei	3 attempts with non-dominant hand, max used	kg	19.6 (4.5)

Author, Year	Measure	Device	Definition and protocol	Units	Mean (SD)
	Walk	N/A	*6MWT:* Distance covered in 6-min walking	Meters	*450 [395, 490]*
	TUG	N/A	Time taken to rise from a chair, walk 3-m, return, and sit back down	Seconds	*6.9 [6.2, 8.1]*
Mizumoto 2015*	Gait	Walk Way MW-1000 pressure sensor	Gait over 2.4-m (central 2-m used) on a pressure sensor, mean of 5 attempts	Dichotomous	N/R
	HGS	Smedley-type dynamometer	2 attempts with dominant hand, max used	Dichotomous	N/R
Nagai 2018*	Gait	N/A	Gait over 12-m (first 10-m used)	Meters/s	1.4 (0.3)
	HGS	Smedley dynamometer	N/R	kg	26.7 (7.6)
Oguma 2017*	HGS	Tanita 6103 dynamometer	2 attempts with dominant hand, max used	kg	19.0 (4.9)
	Chair rise	N/A	N/R	Stands/30s	*11 [9-13]*
	TUG	N/A	N/R	Seconds	*12.1 [9.9-15.9]*
	Balance	N/A	One leg standing test, eyes open	Seconds	*4.0 [2.5-6.5]*
Osuka 2015	Chair rise	N/A	Average of 2 attempts, time taken to complete 5 chair rises	Seconds	*6.8*
	TUG	N/A	Average of 2 attempts, time taken to rise from a chair, walk 3-m, return, and sit back down	Seconds	*6.3*
	Balance	N/A	Average of 2 attempts, single leg stance, eyes open, up to 60s	Seconds	*38.9*
Pina 2021	Gait	N/A	Gait over 10-m (central 6-m used)	Meters/s	*Scot: 1.5 [1.4, 1.7], SA: 1.6 [1.4, 1.7]*
	HGS	*Scot:* T.K.K.5001, Grip-A, Takei. *SA:* T.K.K. 5401, Grip-D	3 attempts with non-dominant hand, max used	kg	*Scot: 23.0 [19.5, 27.5], SA: 20.1 [17.0, 23.8]*
Reid 2016*	TUG	N/A	Time taken to rise from a chair, walk 8-ft, return, and sit back down	Seconds	*5.6 [4.9, 6.5]*
Ribeiro 2020	PF composite score	TKK dynamometer	Composite score between 0-16 was derived from the following tests: 5x Chair rise, HGS, 6MWT, sit-and-reach	Score	*10.0 [8.0-12.0]*
Rojer 2018	Gait	N/A	Gait over 4-m, faster of two attempts used	Meters/s	1.43 (0.21)
	HGS	Jamar dynamometer	3 attempts with each hand, max used	kg	35.1 (11.0)
Sanchez-Sanchez 2019	Gait	N/A	2 attempts, gait over 3-m, fastest used	Meters/s	0.73 (0.26)
	HGS	Jamar dynamometer	3 attempts with dominant hand, max used	kg	22.26 (8.21)
Santos 2012	Chair rise	N/A	No. of chair rises completed in 30-s	n	13.7 (4.7)
	Walk	N/A	*6MWT:* Distance covered in 6-min walking	Meters	450.2 (148.4)
	TUG	N/A	Time taken to rise from a chair, walk 8-ft, return, and sit back down	Seconds	8.5 (5.7)
Savikangas 2020	Gait	N/A	Gait over 10-m	Meters/s	1.98 (0.38)
	Walk	N/A	*6MWT:* Distance covered in 6-min walking	Meters	477.55 (82.56)
	SPPB	N/A	SPPB	Score	10.19 (1.54)
Schrack 2019	Gait	N/A	Gait over 6-m	Meters/s	*Mid ASTP:* 1.21 (0.22)
	Walk	N/A	*400mWT:* Time taken to walk 400-m at a fast pace	Seconds	*Mid ASTP:* 264.84 (51.51
	ExSPPB	N/A	ExSPPB	Score	*Mid ASTP:* 2.99 (0.53)
Spartano 2019	Gait	N/A	Gait: faster of two trials over 4m course	Meters/s	1.17 (0.19)
	HGS	Jamar dynamometer	3 attempts with each hand, max used	kg	*M:* 39.1 (8.7), *F:* 23.3 (5.7)
	Chair rise	N/A	Time taken to complete 5 chair rises	Seconds	9.9 (2.6)
Thiebaud 2020*	Gait	N/A	Gait over 24-m (central 20-m used)	Seconds	1.56 (0.18)
van der Velde 2017	HGS	Jamar dynamometer	3 attempts with each hand, max used	kg	35.7 (10.6)
	Chair rise	N/A	Time taken to complete 10 chair rises	Seconds	23.8 (5.5)
	Walk	N/A	*6MWT:* Distance covered in 6-min walking	Meters	585.1 (80.5)
Ward-Ritacco 2014	Chair rise	N/A	No. of chair rises completed in 30-s	n	21.8 (6.9)

Author, Year	Measure	Device	Definition and protocol	Units	Mean (SD)
	Walk	N/A	*6MWT:* Distance covered in 6-min walking	Meters	651.5 (104.2)
	TUG	N/A	Time taken to rise from a chair, walk 8-ft, return, and sit back down	Seconds	4.5 (0.8)
Ward-Ritacco 2020	Chair rise	N/A	No. of chair rises completed in 30-s	n	20.00 (5.00)
	Walk	N/A	*6MWT:* Distance covered in 6-min walking	Meters	565.8 (68.5)
	TUG	N/A	Time taken to rise from a chair, walk 8-ft, return, and sit back down	Seconds	5.35 (0.86)
Westbury 2018	Gait	N/A	Gait over 3-m	Meter/s	1.0 (0.2)
	HGS	Jamar dynamometer	3 attempts with each hand, max used	kg	24.1 (8.4)
Yamada 2011*	Gait	N/A	Gait over 10-m	Seconds	9.9 (2.2)
	Chair rise	N/A	Time taken to complete 5 chair rises	Seconds	8.9 (3.6)
	TUG	N/A	N/R	Seconds	8.8 (2.1)
	Balance	N/A	Time participant could maintain one-legged stand (hands on waist)	Seconds	13.3 (12.1)
Yasunaga 2017	Gait	N/A	Gait over 11-m (central 5-m used), fastest of 2 attempts	Meters/s	1.3 (0.2)
	HGS	Smedley-type dynamometer	1 attempt with dominant hand	kg	27.4 (8.3)
	TUG	N/A	Time taken to rise from a chair, walk 3-m, return, and sit back down, fastest of 2 attempts	Seconds	6.2 (1.2)
	Balance	N/A	Time (up to max of 60s) participant could maintain one-legged stand eyes open, best of 2 attempts	Seconds	42.9 (21.7)
Yerrakalva 2022	Gait	N/A	Gait over 5-m (first 4-m used)	cm/s	111.4 (25.0)
	HGS	Smedley dynamometer	2 attempts with both hands, max used	kg	28.9 (10.3)
	Chair rise	N/A	Time take to complete 5 chair rises	Stands/min	27.7 (7.7)

*Asterisk denotes not included in meta-analyses, N/A = not applicable, N/R = not reported, HGS = handgrip strength, Gait = gait speed, TUG = timed up-and-go test, 6MWT = 6-minute walk test, 400mWT = 400-meter walk test, kg = kilograms, SPPB = short physical performance battery, M = male, F = female, PF = physical function.
† = reported across six age bands

Table 4. Associations between device-measured physical activity metrics with performance-based physical function outcomes.

Author, Year	PA exposure measure/s	PF outcome measure/s	Adjustment	Effect size (95%CI) or [SE]	p-value
Adachi et al 2018	Step count	Gait	Age + additional	OR = 0.94 (0.73,1.21)	0.695
	MVPA	Gait	Age + additional	OR = 0.94 (0.73,0.99)	**0.031**
Aggio 2016*	LPA	Gait	Age + additional	B = 0.02 (0.02, 0.03)	**<0.001**
	LPA	HGS	Age + additional	B = 0.21 (-0.06, 0.48)	0.125
	MVPA	Gait	Age + additional	B = 0.03 (0.02, 0.03)	**<0.001**
	MVPA	HGS	Age + additional	B = 0.58 (0.34, 0.82)	**<0.001**
Aoyagi 2009	Step count	Gait	Age and/or sex	R = 0.31	**<0.05**
	Step count,	HGS	Age and/or sex	R = 0.12	>0.05
	Step count,	Balance	Age and/or sex	R = -0.14, -0.15	>0.05
	TPA (PA >3METs)	Gait	Age and/or sex	R = 0.34	**<0.05**
	TPA (PA >3METs)	HGS	Age and/or sex	R = 0.12	>0.05
	TPA (PA >3METs)	Balance	Age and/or sex	R = -0.13, -0.13	>0.05
Cooper 2015	TPA (PAEE)	HGS	Sex	β‡ = 0.632 (0.158, 1.105)	**<0.05**
	TPA (PAEE)	Chair rise	Sex	β‡ = 0.943 (0.594, 1.292)	**<0.05**
	TPA (PAEE)	TUG	Sex	β‡ = 0.029 (0.021, 0.036)	**<0.05**
	TPA (PAEE)	Balance	Sex	β‡ = 0.073 (0.047, 0.099)	**<0.05**
	MVPA	HGS	Sex	β‡ = 0.638 (0.166, 1.110)	**<0.05**
	MVPA	Chair rise	Sex	β‡ = 0.670 (0.321, 1.018)	**<0.05**
	MVPA	TUG	Sex	β‡ = 0.023 (0.016, 0.031)	**<0.05**
	MVPA	Balance	Sex	β‡ = 0.036 (0.010, 0.062)	**<0.05**
Cooper 2020	TPA	HGS	Sex + additional	B = 0.60 (0.30, 0.90)	N/R
	MVPA	HGS	Additional	*M:* B = −1.17 (−2.01, −0.33), *F:* B = 0.73 (0.19, 1.27)	N/R, N/R
Davis 2014	MVPA	Gait	Age, sex + additional	B = 0.659 (0.398, 0.920)	**<0.001**
	MVPA	Chair rise	Age, sex + additional	B = 0.851 (0.429, 1.272)	**<0.001**
	MVPA	Balance	Age, sex + additional	B = 0.269 (0.005, 0.532)	**0.046**
Duck 2019	LPA	TUG	Unadjusted	R = −0.404	**<0.01**
	LPA	Balance	Age, sex + additional	B = 0.013 (0.011), β = 0.146	Non-sig.
	MPA	TUG	Unadjusted	R = -0.363	**<0.01**
	MPA	Balance	Age, sex + additional	B = -0.006 (0.049), β = -0.013	Non-sig.
	VPA	TUG	Unadjusted	R = −0.105	**<0.01**
	VPA	Balance	Unadjusted	R = 0.091	Non-sig.
	MVPA	TUG	Unadjusted	R = -0.337	N/R
	MVPA	Balance	Unadjusted	R = 0.270	N/R
Gobbo 2020	MVPA	Gait	Age + additional	*M:* B = 0.01 (-0.00, 0.02), *F:* B = 0.00 (-0.00, 0.00)	>0.05, >0.05
		HGS	Age + additional	*M:* B = -0.08 (-0.21, 0.04), *F:* B = -0.05 (-0.14, 0.03)	>0.05, >0.05
		TUG	Age + additional	*M:* B = -0.02 (-0.14, 0.09), *F:* B = 0.02 (-0.09, 0.14)	>0.05, >0.05
Hall 2017	Step count	Gait	Unadjusted	R†	N/A
	Step count	Chair rise	Unadjusted	R†	N/A
	Step count	Walk	Unadjusted	R†	N/A
	Step count	Balance	Unadjusted	R†	N/A
	LPA	Gait	Unadjusted	R†	N/A
	LPA	Chair rise	Unadjusted	R†	N/A
	LPA	Walk	Unadjusted	R†	N/A
	LPA	Balance	Unadjusted	R†	N/A

Author, Year	PA exposure measure/s	PF outcome measure/s	Adjustment	Effect size (95%CI) or [SE]	p-value
	MVPA	Gait	Unadjusted	R†	N/A
	MVPA	Chair rise	Unadjusted	R†	N/A
	MVPA	Walk	Unadjusted	R†	N/A
	MVPA	Balance	Unadjusted	R†	N/A
Hsueh 2020	Step count	Gait	Age + additional	*M:* β = −0.19 (−0.60, 0.22), *F:* β = −0.31 (−0.57, −0.001)	0.35, **0.049**
	Step count	HGS	Age + additional	*M:* β = 0.04 (−0.44, 0.51), *F:* β = 0.46 (0.12, 0.78)	0.87, **0.009**
	Step count	Chair rise	Age + additional	*M:* β = 0.30 (−0.36, 0.96), *F:* β = −0.35 (−0.70, 0.01)	0.36, 0.05
	Step count	TUG	Age + additional	*M:* β = −0.09 (−0.50, 0.32), *F:* β = −0.20 (−0.50, 0.12)	0.66, 0.22
	Step count	Balance	Age + additional	*M:* β = 0.16 (−0.32, 0.63), *F:* β = 0.26 (−0.04, 0.55)	0.50, 0.09
	TPA	Gait	Age + additional	*M:* β = −0.24 (−0.64, 0.17), *F:* β = −0.11 (−0.36, 0.16)	0.23, 0.44
	TPA	HGS	Age + additional	*M:* β = 0.07 (−0.39, 0.53), *F:* β = 0.21 (−0.10, 0.52)	0.75, 0.19
	TPA	Chair rise	Age + additional	*M:* β = −0.13 (−0.78, 0.52), *F:* β = −0.23(−0.54, 0.10)	0.69, 0.17
	TPA	TUG	Age + additional	*M:* β = −0.30 (−0.69, 0.10), *F:* β = −0.15 (−0.42, 0.13)	0.14, 0.31
	TPA	Balance	Age + additional	*M:* β = 0.23 (−0.23, 0.68), *F:* β = 0.06 (−0.21, 0.33)	0.31, 0.67
	MVPA	Gait	Age + additional	*M:* β = −0.24 (−0.57, 0.08), *F:* β = −0.12 (−0.36, 0.11)	0.13, 0.29
	MVPA	HGS	Age + additional	*M:* β = 0.07 (−0.31, 0.45), *F:* β = 0.39 (0.12, 0.64)	0.70, **0.004**
	MVPA	Chair rise	Age + additional	*M:* β = 0.05 (−0.50, 0.60), *F:* β = −0.22 (−0.49, 0.05)	0.85, 0.11
	MVPA	TUG	Age + additional	*M:* β = −0.19 (−0.51, 0.14), *F:* β = −0.13 (−0.37, 0.12)	0.24, 0.32
	MVPA	Balance	Age + additional	*M:* β = 0.23 (−0.15, 0.59), *F:* β = 0.25 (0.02, 0.49)	0.23, **0.036**
Izawa 2017	MVPA (MPA)	Gait	Age + additional	*M:* β = 0.310 (0.001, 0.004), *F:* β = 0.396, (0.002, 0.006)	**0.001**, **0.001**
	MVPA (MPA)	TUG	Age + additional	*M:* β = -0.321 (-0.015, -0.006), *F:* β = -0.473 (-0.031, -0.014)	**0.001, 0.001**
	MVPA (MPA)	Balance	Age + additional	*M:* β = 0.217 (0.042, 0.208), *F:* β = 0.252 (0.048, 0.355)	**0.003**, **0.011**
Jantunen 2017	TPA	Chair rise	Age, sex	β = 0.06 (0.05, 0.07)	**<0.001**
	TPA	Walk	Age, sex	β = 0.09 (0.08, 0.10)	**<0.001**
	TPA	SFT	Age, sex	β = 0.08 (0.07, 0.10)	**<0.001**
	LPA	SFT	Age, sex	β = 0.09 (0.07, 0.12)	**<0.001**
	MVPA	SFT	Age, sex	β = 0.10 (0.08, 0.11)	**<0.001**
Johansson 2021	LPA	HGS	Age	N/R	N/R
	LPA	Chair rise	Age	N/R	N/R
	MVPA	HGS	Age	*M:* β = -0.09, *F:* β = 0.08	**<0.001, 0.001**
	MVPA	Chair rise	Age	*M:* β = 0.31, *F:* β = -0.26	**<0.001, <0.001**
Kim 2015	TPA (PA)	Gait	Age, sex	R_s = 0.231	**0.001**
	TPA (PA)	HGS	Age, sex	R_s = 0.081	0.251
Kruger 2016	TPA (PAEE)	Gait	Age + additional	β = 0.15	**0.04**
	TPA (PAEE)	HGS	Age + additional	β = 0.07	0.45
Lai 2020	MVPA	Gait	Age, sex + additional	B = −0.061 (−0.091, −0.031)	**<0.001**
	MVPA	HGS	Age, sex + additional	B = 0.045 (0.017, 0.072)	**0.002**
	MVPA	Chair rise	Age, sex + additional	B = −0.037 (−0.081, 0.006)	0.094
	MVPA	TUG	Age, sex + additional	B = −0.045 (−0.079, −0.011)	**0.009**
Lerma 2018	LPA	Gait	Age, sex + additional	B = 0.026 (-0.014, 0.066)	>0.05
	LPA	Chair rise	Age, sex + additional	B = −0.622 (-1.349, 0.104)	>0.05
	LPA	Walk	Age, sex + additional	B = 0.064 (0.013, 0.116)	**<0.05**
	LPA	SPPB	Age, sex + additional	B = 0.430 (-0.015, 0.876)	>0.05
	MVPA	Gait	Age, sex + additional	B = 0.295 (0.146, 0.444)	**<0.05**
	MVPA	Chair rise	Age, sex + additional	B = -4.433 (−7.217, −1.650)	**<0.05**
	MVPA	Walk	Age, sex + additional	B = 0.407 (0.219, 0.595)	**<0.05**
	MVPA	SPPB	Age, sex + additional	B = 3.233 (1.045, 5.422)	**<0.05**
Lohne-Seiler 2016	Step count	HGS	Age, sex + additional	B = -0.133^^^ (-0.61, 0.34)	>0.05

Author, Year	PA exposure measure/s	PF outcome measure/s	Adjustment	Effect size (95%CI) or [SE]	p-value
	Step count	Balance	Age, sex + additional	B = 1.88 (0.85, 2.90)	**<0.05**
Manas 2019*	LPA (SB ratio)	SPPB	Age, sex + additional	B = 0.96 (0.09, 1.82)	**0.03**
	MVPA (SB ratio)	SPPB	Age, sex + additional	B = 0.03 (0.02, 0.04)	**< 0.001**
Meier 2020	Step count	Gait	Age, sex + additional	β = 0.01 [0.004]	0.05
	Step count	HGS	Age, sex + additional	β = 0.01 [0.16]	0.53
Mendham 2021	TPA	Gait	Age	N/R	N/R
	TPA	HGS	Age	N/R	N/R
	TPA	Walk	Age	N/R	N/R
	TPA	TUG	Age	N/R	N/R
	LPA	Gait	Age	N/R	N/R
	LPA	HGS	Age	N/R	N/R
	LPA	Walk	Age	N/R	N/R
	LPA	TUG	Age	N/R	N/R
	MVPA	Gait	Age	N/R	N/R
	MVPA	HGS	Age	N/R	N/R
	MVPA	Walk	Age	N/R	N/R
	MVPA	TUG	Age	N/R	N/R
Mizumoto 2015*	Step count	Gait	Age, sex + additional	OR = 1.72 (0.77, 3.86)	>0.05
	Step count	HGS	Age, sex + additional	OR = 2.89 (1.10, 7.58)	**<0.05**
	MVPA	Gait	Age, sex + additional	OR = 0.74 (0.33, 1.64)	>0.05
	MVPA	HGS	Age, sex + additional	OR = 1.86 (0.71, 4.89)	>0.05
Nagai 2018*	LPA	Gait	Unadjusted	R_{pb} = -0.30	**<0.01**
	LPA	HGS	Unadjusted	R_{pb} = -0.16	**<0.01**
	MVPA	Gait	Unadjusted	R_{pb} = -0.17	**<0.01**
	MVPA	HGS	Unadjusted	R_{pb} = -0.12	**<0.01**
Oguma 2017*	Step count	HGS	Unadjusted	R_s = 0.24	**0.003**
	Step count	Chair rise	Unadjusted	R_s = 0.35	**<0.001**
	Step count	TUG	Unadjusted	R_s = -0.51	**<0.001**
	Step count	Balance	Unadjusted	R_s = 0.32	**<0.001**
	TPA (PA Index)	HGS	Unadjusted	R_s = 0.28	**<0.001**
	TPA (PA Index)	Chair rise	Unadjusted	R_s = 0.39	**<0.001**
	TPA (PA Index)	TUG	Unadjusted	R_s = -0.56	**<0.001**
	TPA (PA Index)	Balance	Unadjusted	R_s = 0.34	**<0.001**
Osuka 2015	LPA	Chair rise	Age, sex + additional	β = -0.07	**0.047**
	LPA	TUG	Age, sex + additional	β = -0.08	**0.013**
	LPA	Balance	Unadjusted	R_s = 0.23	**<0.001**
Pina 2021	LPA	Gait	Age, sex + additional	γ = −0.012	0.876
	LPA	HGS	Age, sex + additional	γ = −0.045	0.644
	MVPA	Gait	Age, sex + additional	γ = 0.007	0.773
	MVPA	HGS	Age, sex + additional	γ = 0.097	**0.001**
Reid 2016*	Step count (All stepping)	TUG	Age, sex + additional	RR = 0.98 (0.95, 1.02)	0.341
	LPA (Light stepping)	TUG	Age, sex + additional	RR = 0.98 (0.93, 1.03)	0.378
	MVPA (MVPA stepping)	TUG	Age, sex + additional	RR = 0.97 (0.92, 1.03)	0.383
	Sit-to-stand transitions	TUG	Age, sex + additional	RR = 1.00 (1.00, 1.00)	0.961
Ribeiro 2020	Active/Inactive	PF composite score	Age, sex + additional	OR = 1.81 (0.95, 3.46)	0.074
Rojer 2018	TPA	HGS	Age, sex	*Y:* B = 0.001 [0.001] *O:* B = 0.002 [0.001]	>0.05, >0.05
	TPA	Gait	Age, sex	*Y:* B = 0.001 [0.001] *O:* B = 0.005 [0.002]	>0.05, **<0.05**
	Step count	HGS	Age, sex	*Y:* B = 0.051 [0.024] *O:* B = 0.052 [0.038]	**<0.05**, >0.05
	Step count	Gait	Age, sex	*Y:* B = 0.026 [0.027] *O:* B = 0.182 [0.041]	>0.05, **<0.05**

Author, Year	PA exposure measure/s	PF outcome measure/s	Adjustment	Effect size (95%CI) or [SE]	p-value
Sanchez-Sanchez 2019	TPA	Gait	Age, sex + additional	B = 0.041 (0.019, 0.063)	**<0.001**
	TPA	HGS	Age, sex + additional	B = 0.857 (0.312, 1.402)	**<0.01**
	LPA	Gait	Age, sex + additional	B = -0.006 (-0.021, 0.009)	>0.05
	LPA	HGS	Age, sex + additional	B = 0.428 (0.051, 0.805)	**<0.05**
	MVPA	Gait	Age, sex + additional	B = 0.070 (0.043, 0.097)	**<0.001**
	MVPA	HGS	Age, sex + additional	B = 0.933 (0.246, 1.620)	**<0.01**
Santos 2012	MVPA	Chair rise	Age, sex + additional	B = 0.035 (0.014, 0.055)	N/R
		Walk	Age, sex + additional	B = 1.770 (1.178, 2.632)	N/R
		TUG	Age, sex + additional	B = -0.023 (-0.049, 0.003)	N/R
Savikangas 2020	LPA	Gait	Age, sex	R = 0.203	**<0.01**
	LPA	Walk	Age, sex	R = 0.279	**<0.001**
	LPA	SPPB	Age, sex	R = 0.145	**<0.01**
	MVPA	Gait	Age, sex	R = 0.315	**<0.001**
	MVPA	Walk	Age, sex	R = 0.465	**<0.001**
	MVPA	SPPB	Age, sex	R = 0.220	**<0.001**
Schrack 2019	TPA (Log activity counts)	Gait	Age, sex + additional	0.11 [0.04]	**0.004**
		Walk	Age, sex + additional	-0.16 [0.03]	**<0.001**
		ExSPPB	Age, sex + additional	0.13 [0.04]	**<0.001**
Spartano 2019	Step count	Gait	Age, sex + additional	B = 0.006 [0.001]	**0.0001**
	Step count	HGS	Age, sex + additional	*M:* B = -0.16 [0.09], *F:* B = 0.09 [0.06]	0.077, 0.125
	Step count	Chair rise	Age, sex + additional	B = -0.010 [0.002]	**<0.0001**
	MVPA	Gait	Age, sex + additional	B = 0.048 [0.005]	**<0.0001**
	MVPA	HGS	Age, sex + additional	*M:* B = 0.58 [0.34], *F:* B = 0.64 [0.19]	0.090, **0.0008**
	MVPA	Chair rise	Age, sex + additional	B = -0.057 [0.006]	**<0.0001**
Thiebaud 2020*	LPA	Gait	Age + additional	β = -0.250	**0.016**
	MPA	Gait	Age + additional	β = -0.112	0.337
	VPA	Gait	Age + additional	β = 0.357	**0.003**
van der Velde 2017	TPA, High intensity PA	HGS	Age, sex + additional	B = 0.02 (0.01; 0.03)	**<0.05**
	TPA	Chair rise	Age, sex + additional	B = −0.88 (−1.24; −0.52)	**<0.05**
	TPA	Walk	Age, sex + additional	B = 24.45 (19.74, 29.15)	**<0.05**
	High intensity PA	HGS	Age, sex + additional	B = 0.04 (0.03; 0.06)	**<0.05**
	High intensity PA	Chair rise	Age, sex + additional	B = −2.82 (−3.62; −2.03)	**<0.05**
	High intensity PA	Walk	Age, sex + additional	B = 61.25 (50.73, 71.77)	**<0.05**
Ward-Ritacco 2014	Step count	Chair rise	Age + additional	β = 0.23 (0.000, 0.001)	>0.05
	Step count	Walk	Age + additional	β = 0.31 (0.002, 0.01)	**<0.01**
	Step count	TUG	Age + additional	β = -0.16 (0.000, 0.000)	>0.05
	MVPA	Chair rise	Age + additional	R = 0.38	**<0.01**
	MVPA	Walk	Age + additional	R = 0.50	**<0.01**
	MVPA	TUG	Age + additional	R = -0.32	**<0.05**
Ward-Ritacco 2020	Step count	Chair rise	Age + additional	B = 0.67 (0.28, 1.05)	**0.001**
		Walk	Age + additional	B = 4.09 (-0.85, 9.03)	0.103
		TUG	Age + additional	B = −0.04 (-0.11, 0.02)	0.200
Westbury 2018	TPA	Gait	Sex	B = 0.29 (0.12, 0.47)	**<0.001**
	TPA	HGS	Sex	B = 0.15 (-.02, 0.33)	0.08
	MVPA	Gait	Sex	B = 0.19 (0.01, 0.37)	**0.04**
	MVPA	HGS	Sex	B = 0.10 (-0.08, 0.27)	0.29
Yamada 2011*	Step count	Gait	Unadjusted	R = -0.475	**<0.01**
		Chair rise	Unadjusted	R = -0.297	**<0.01**
		TUG	Unadjusted	R = -0.412	**<0.01**

Author, Year	PA exposure measure/s	PF outcome measure/s	Adjustment	Effect size (95%CI) or [SE]	p-value
		Balance	Unadjusted	R = 0.440	**<0.01**
Yasunaga 2017	LPA	Gait	Age, sex + additional	B = 0.001 (-0.001, 0.004)	>0.05
	LPA	HGS	Age, sex + additional	B = 0.058 (-0.024, 0.141)	>0.05
	LPA	TUG	Age, sex + additional	B = -0.011 (-0.025, 0.004)	>0.05
	LPA	Balance	Age, sex + additional	B = 0.139 (-0.131, 0.409)	>0.05
	MVPA	Gait	Age, sex + additional	B = 0.019 (0.011, 0.026)	**<0.001**
	MVPA	HGS	Age, sex + additional	B = 0.092 (-0.135, 0.318)	>0.05
	MVPA	TUG	Age, sex + additional	B = -0.155 (-0.153, -0.077)	**<0.001**
	MVPA	Balance	Age, sex + additional	B = 1.187 (0.462, 1.913)	**<0.01**
Yerrakalva 2022	TPA	HGS	Age, sex + additional	B = 0.1 (-0.2, 0.4)	>0.05
	TPA	Gait	Age, sex + additional	B = 4.4 (2.0, 6.7)	**<0.05**
	TPA	Chair rise	Age, sex + additional	B = 1.1 (0.7, 1.4)	**<0.05**
	LPA	HGS	Age, sex + additional	B = -0.04 (-0.5, 0.4)	>0.05
	LPA	Gait	Age, sex + additional	B = 3.0 (1.8, 4.2)	**<0.05**
	LPA	Chair rise	Age, sex + additional	B = 0.6 (0.4, 0.8)	**<0.05**
	MVPA	HGS	Age, sex + additional	B = 0.2 (-0.2, 0.6)	>0.05
	MVPA	Gait	Age, sex + additional	B = 5.4 (4.2, 6.0)	**<0.05**
	MVPA	Chair rise	Age, sex + additional	B = 1.2 (0.6, 1.8)	**<0.05**

*Asterisk denotes not included in meta-analyses, N/A = not applicable, N/R = not reported, B = unstandardised regression coefficient, β = standardised regression coefficient, R = correlation coefficient, R_{pb} = point biserial correlation, γ = compositional linear regression coefficient, † = reported across six age bands, ‡ = standardised by physical activity exposure only, LPA = light intensity physical activity, MVPA = moderate-to-vigorous physical activity, Steps = average or total step count, TPA = total physical activity, HGS = handgrip strength, Gait = gait speed, TUG = timed up-and-go test.
