# Supplementary material for: Associations between device-measured physical activity and performance-based physical function outcomes in adults: a systematic review and meta-analysis
Source: BMJ Public Health. 2023 Oct 30;1(1):e100000. doi: 10.1136/bmjph-2023-100000 (PMC11812739; doi:10.1136/bmjph-2023-100000)
Supplement: online supplemental file 4 [file bmjph-1-1-s004.pdf]

Table 1. Quality assessment of the methodological quality of included studies

Author	Q1	Q2	Q3	Q4	Q5	Q6	Q7	Q8	Q9	Q10	Q11	Q12	Q13	Q14	Q15	Total
Adachi (2018)	y	y	nr	y	n	na	na	y	y	n	n	y	nr	y	y	8
Aggio (2016)	y	y	y	y	n	na	na	y	y	y	n	y	nr	y	y	10
Aoyagi (2009)	y	y	nr	y	n	na	na	n	y	n	n	y	nr	nr	y	6
Cooper (2015)	y	y	y	y	n	na	na	y	y	y	n	y	nr	n	y	9
Cooper (2020)	y	y	y	y	n	na	na	y	y	y	n	y	nr	n	y	9
Davis (2014)	y	y	nr	y	n	na	na	n	y	y	n	y	nr	y	y	8
Duck (2019)	y	y	nr	y	n	na	na	n	y	y	n	y	nr	y	y	8
Gobbo (2020)	y	y	n	y	y	y	y	n	y	y	n	y	nr	n	y	10
Hall (2017)	y	y	nr	y	n	na	na	y	y	y	n	y	nr	n	n	7
Hsueh (2020)	y	y	nr	y	n	na	na	y	y	y	n	y	nr	n	y	8
Izawa (2017)	y	y	n	y	n	na	na	y	y	n	n	y	nr	y	y	8
Jantunen (2017)	y	y	y	y	n	na	na	y	y	n	n	y	nr	n	y	8
Johansson (2021)	y	y	y	y	n	na	na	y	y	y	n	y	nr	n	y	9
Kim (2015)	y	y	y	y	n	na	na	n	y	n	n	y	nr	n	y	7
Kruger (2016)	y	y	nr	y	y	na	na	n	y	n	n	y	nr	nr	y	7
Lai (2020)	y	y	nr	y	n	na	na	y	y	y	n	y	nr	y	y	9
Lerma (2018)	y	y	nr	y	n	na	na	y	y	n	n	y	nr	y	y	8
Lohne-Seiler (2016)	y	y	n	y	n	na	na	n	y	n	n	y	nr	y	y	7
Manas (2019)	y	y	nr	y	n	na	na	y	y	y	n	y	nr	y	y	9
Meier (2020)	y	y	nr	y	n	na	na	n	y	n	n	y	nr	nr	y	6
Mendham (2021)	y	y	nr	y	y	na	na	y	y	n	n	y	nr	nr	y	8
Mizumoto (2015)	y	y	n	y	n	n	n	y	y	n	y	y	nr	n	y	8
Nagai (2018)	y	y	nr	y	n	na	na	y	y	y	n	y	nr	y	n	8
Oguma (2017)	y	y	y	y	n	na	na	n	y	y	n	y	nr	y	n	8
Osuka (2015)	y	y	y	y	n	na	na	y	y	y	n	y	nr	n	y	9
Pina (2021)	y	y	nr	n	n	na	na	y	y	y	n	y	nr	y	y	8
Reid (2016)*	y	y	n	y	y	na	na	y	y	y	n	y	nr	nr	y	9
Ribeiro (2020)	y	y	y	y	y	na	na	n	y	y	n	y	nr	n	y	9
Rojer (2018)	Y	Y	nr	Y	n	na	na	y	y	y	n	y	nr	y	y	9
Sanchez-Sanchez (2019)	y	y	nr	y	n	na	na	y	y	y	n	y	nr	y	y	9
Santos (2012)	y	y	nr	y	n	na	na	n	y	y	n	n	nr	nr	y	6
Savikangas (2020)	y	y	n	y	n	na	na	y	y	y	n	y	nr	n	y	8
Schrack (2019)	y	y	nr	y	n	na	na	n	y	y	n	y	nr	nr	y	7

Author	Q1	Q2	Q3	Q4	Q5	Q6	Q7	Q8	Q9	Q10	Q11	Q12	Q13	Q14	Q15	Total
Spartano (2019)	y	y	y	y	n	na	na	y	y	y	n	y	nr	nr	y	9
Thiebaud (2020)*	y	n	nr	n	n	na	na	y	y	y	n	y	nr	nr	y	6
van der Velde (2017)	y	y	y	y	n	na	na	y	y	y	n	y	nr	y	y	10
Ward-Ritacco (2014)	y	y	nr	y	n	na	na	y	y	y	n	y	nr	nr	y	8
Ward-Ritacco (2020)	y	y	nr	y	y	na	na	n	y	y	n	y	nr	nr	y	8
Westbury (2018)	y	y	n	y	n	na	na	y	y	n	n	y	nr	y	n	7
Yamada (2011)	y	y	nr	n	n	na	na	n	y	n	n	n	nr	nr	n	3
Yasunaga (2017)	y	y	n	y	n	na	na	y	y	y	n	y	nr	y	y	9
Yerrakalva (2022)	y	y	y	y	n	y	y	y	y	y	y	y	nr	y	y	13

y; yes. n; no. na; not applicable. nr; not reported.

Q1 - Was the research question or objective in this paper clearly stated? Q2 - Was the study population clearly specified and defined? Q3 - Was the participation rate of eligible participants >50%? Q4 - Were all the subjects selected or recruited from the same or similar populations (including the same time period)? Were inclusion and exclusion criteria for being in the study prespecified and applied uniformly to all participants? Q5 - Was a sample size justification, power description, or variance and effect estimates provided? Q6 - For the analyses in this paper, were the exposure(s) of interest measured prior to the outcome(s) being measured? Q7 - Was the timeframe sufficient so that one could reasonably expect to see an association between exposure and outcome if it existed? Q8 - For exposures that can vary in amount or level, did the study examine different levels of the exposure as related to the outcome (e.g., categories of exposure, or exposure measured as continuous variable)? Q9 - Were the exposure measures (independent variables) clearly defined, valid, reliable, and implemented consistently across all study participants? Q10 - Was the accelerometer protocol reported up to the standards of the Montoye et al. (2018) guidelines? Reporting of; brand, epoch, placement, days, valid hours/days, non-wear criteria, accelerometer outcomes and interpretation (e.g. MVPA and cut points used) Q11 - Was the exposure(s) assessed more than once over time? Q12 - Were the outcome measures (dependent variables) clearly defined, valid, reliable, and implemented consistently across all study participants? Q13 - Were the outcome assessors blinded to the exposure status of participants? Q14 - Was loss to follow-up after baseline 20% or less? Q15 - Were key potential confounding variables measured and adjusted statistically for their impact on the relationship between exposure(s) and outcome(s) – with age + sex the minimum?

	QA score Mean (SD)	QA score Range
**All reports (n = 42)**	8.1 (1.5)	3, 13
**Reports included in meta-analyses (k = 34)**	8.2 (1.3)	6, 13
