# Supplementary material for: Associations between device-measured physical activity and performance-based physical function outcomes in adults: a systematic review and meta-analysis
Source: BMJ Public Health. 2023 Oct 30;1(1):e100000. doi: 10.1136/bmjph-2023-100000 (PMC11812739; doi:10.1136/bmjph-2023-100000)
Supplement: online supplemental file 5 [file bmjph-1-1-s005.pdf]

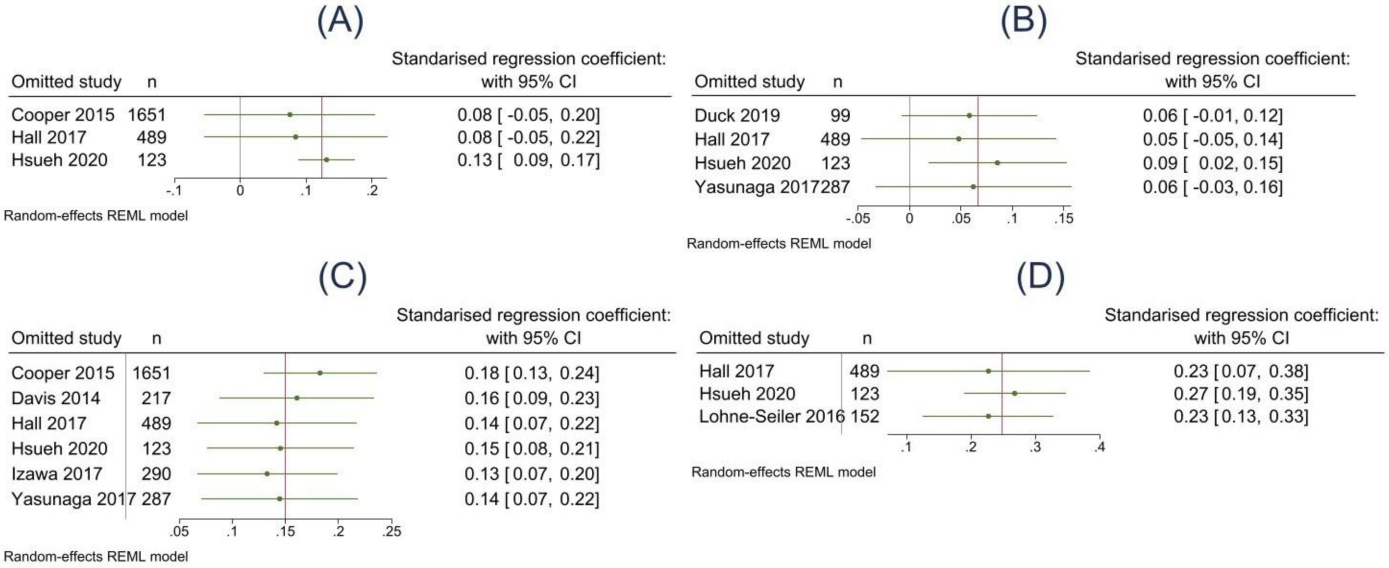

Figure 1. Balance leave-one-out sensitivity analysis for the association with (A) total physical activity (B) light physical activity (C) moderate-to-vigorous physical activity (D) step count

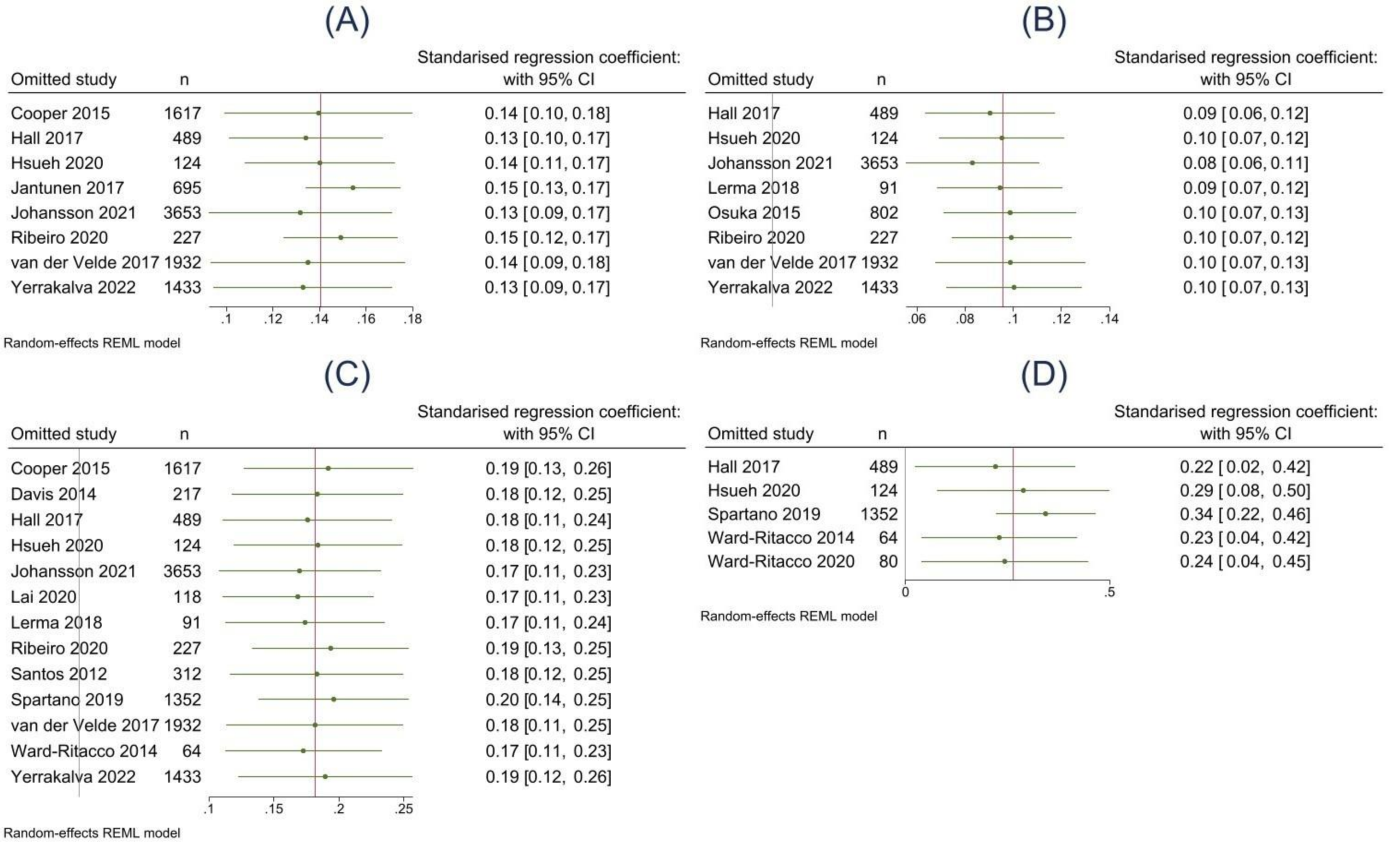

Figure 2. Chair rise test leave-one-out sensitivity analysis for the association with (A) total physical activity (B) light physical activity (C) moderate-to-vigorous physical activity (D) step count

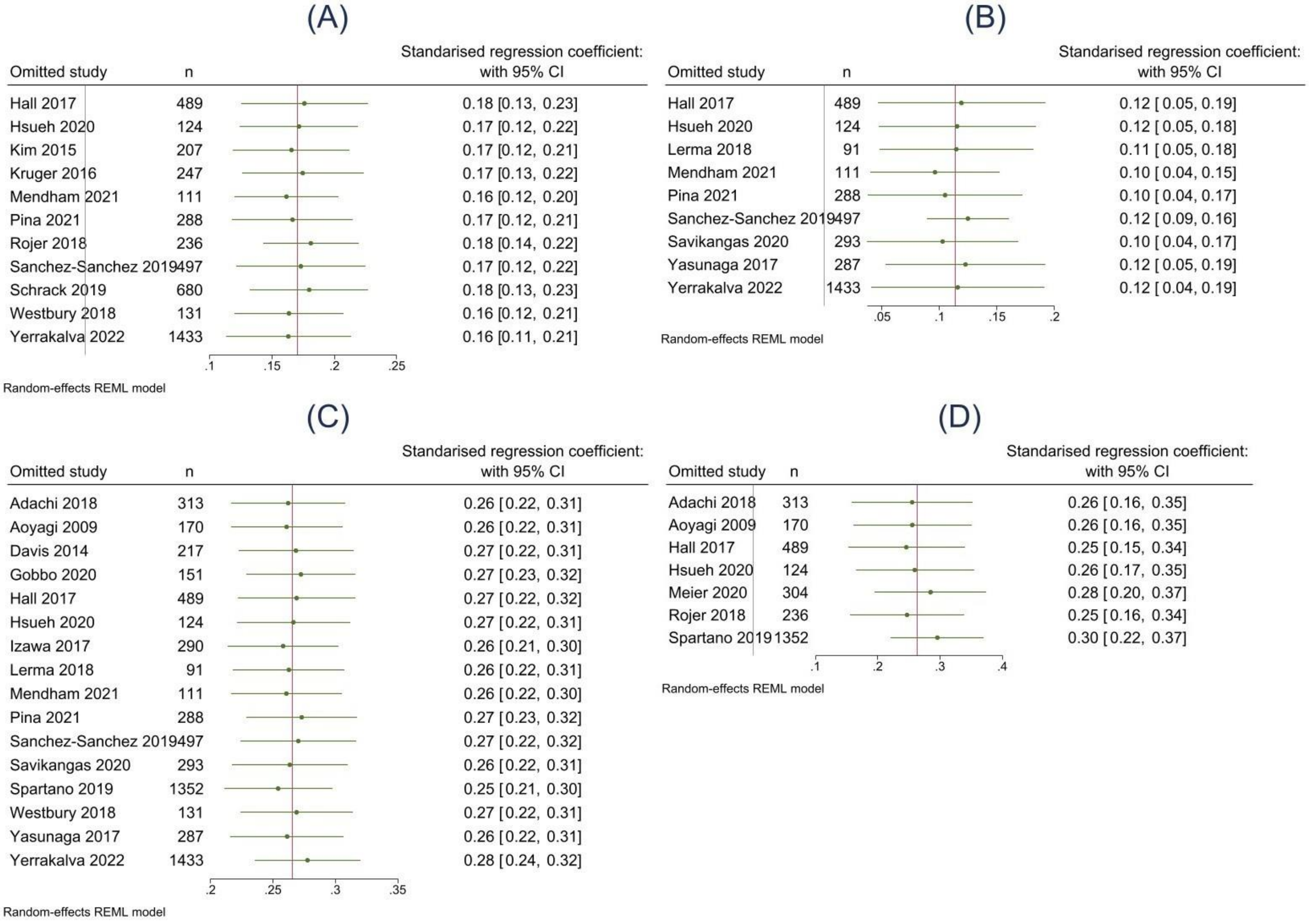

Figure 3. Gait speed leave-one-out sensitivity analysis for the association with (A) total physical activity (B) light physical activity (C) moderate-to-vigorous physical activity (D) step count

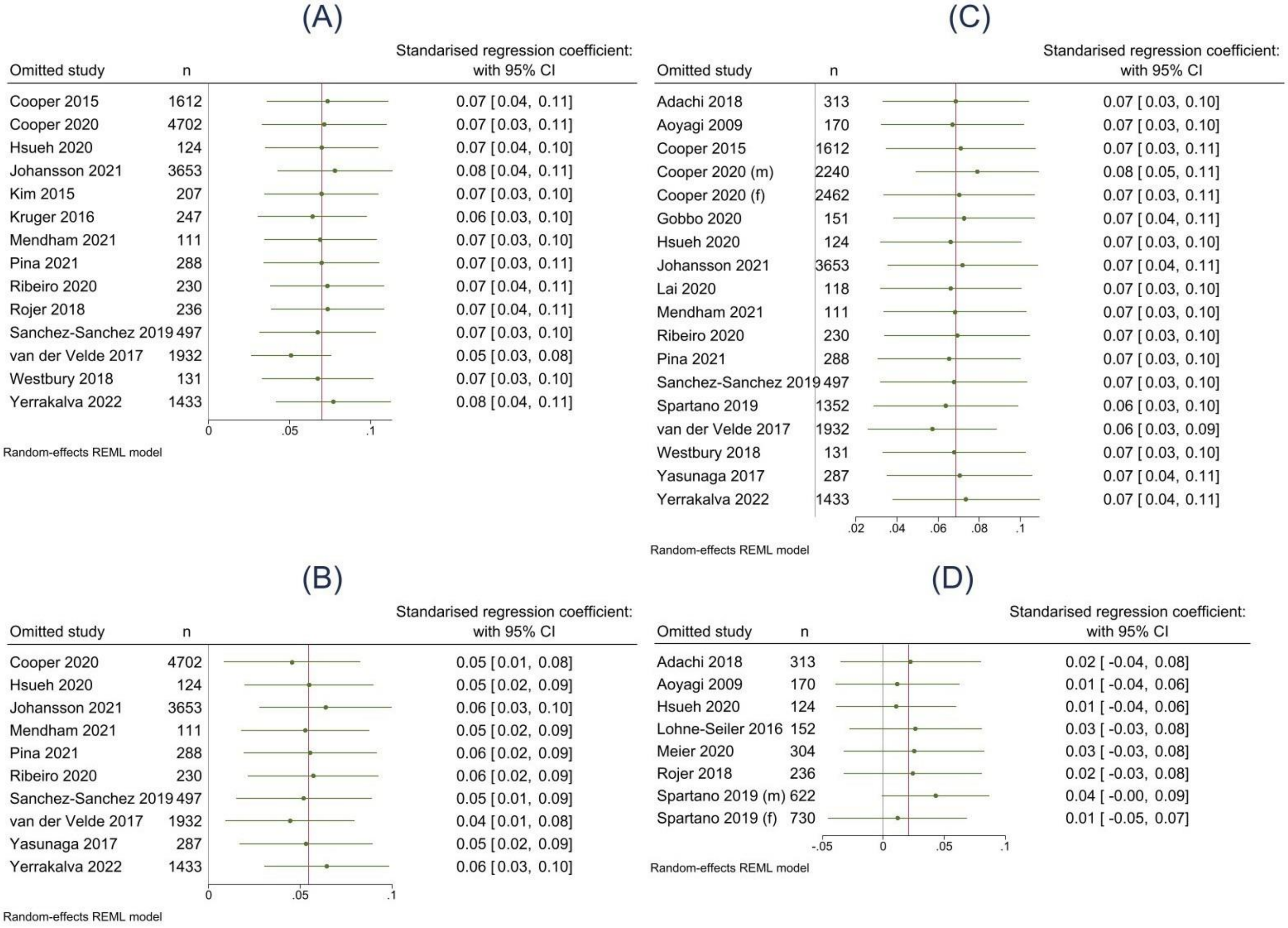

Figure 4. Handgrip strength leave-one-out sensitivity analysis for the association with (A) total physical activity (B) light physical activity (C) moderate-to-vigorous physical activity (D) step count

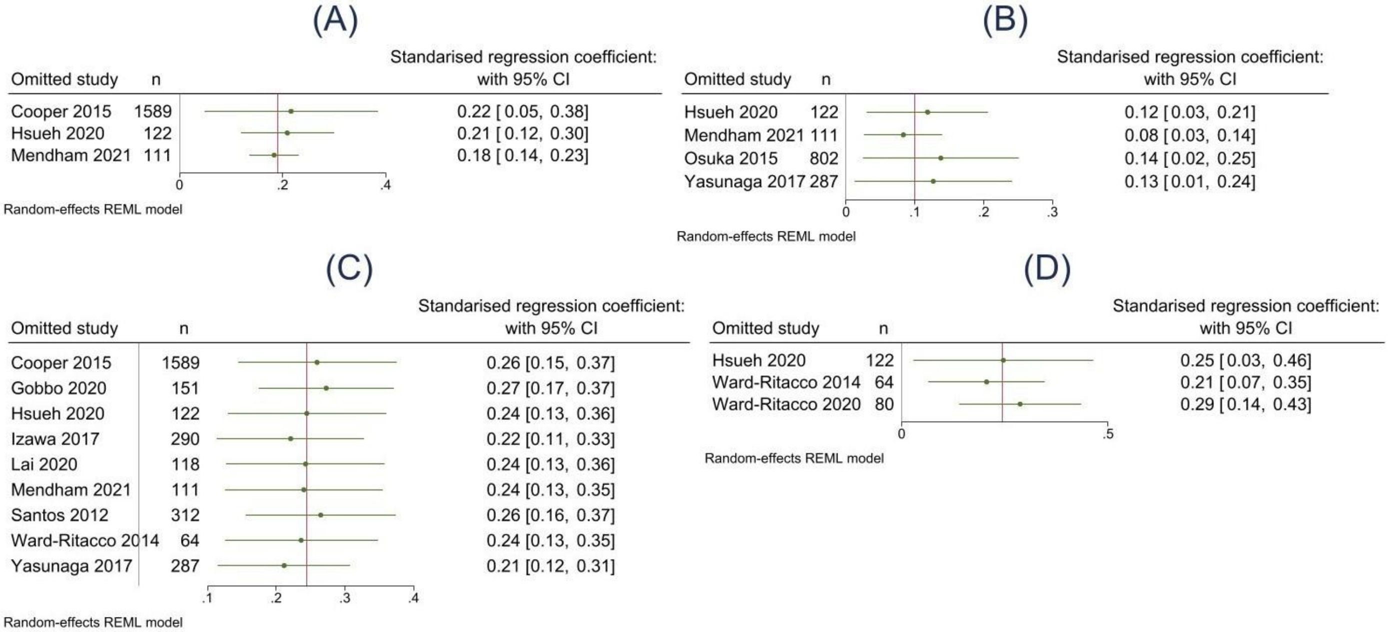

Figure 5. Timed up-and-go leave-one-out sensitivity analysis for the association with (A) total physical activity (B) light physical activity (C) moderate-to-vigorous physical activity (D) step count

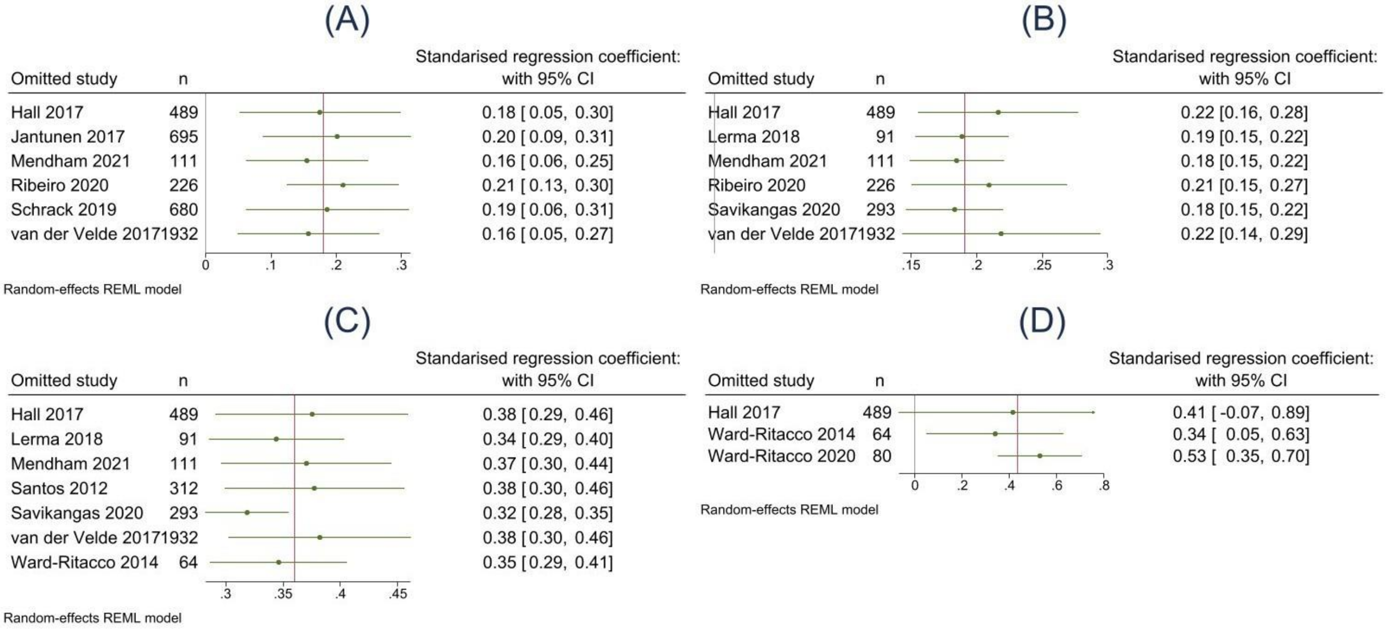

Figure 6. Walk test leave-one-out sensitivity analysis for the association with (A) total physical activity (B) light physical activity (C) moderate-to-vigorous physical activity (D) step count
