# Supplementary material for: Associations between device-measured physical activity and performance-based physical function outcomes in adults: a systematic review and meta-analysis
Source: BMJ Public Health. 2023 Oct 30;1(1):e100000. doi: 10.1136/bmjph-2023-100000 (PMC11812739; doi:10.1136/bmjph-2023-100000)
Supplement: online supplemental file 6 [file bmjph-1-1-s006.pdf]

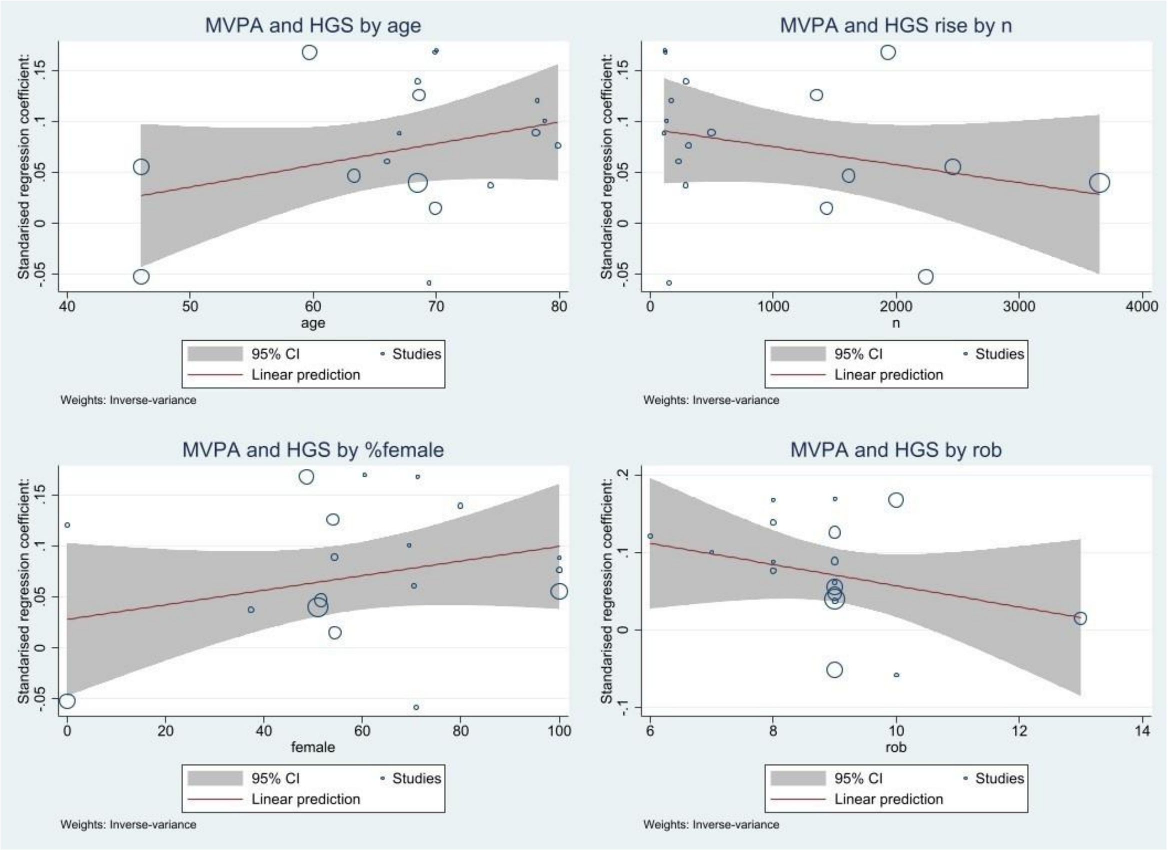

Figure 1. Bubble plots of meta-regression for moderate-to-vigorous physical activity and handgrip strength for; age, sample size (n), percentage of females per study, and risk of bias (quality assessment score)

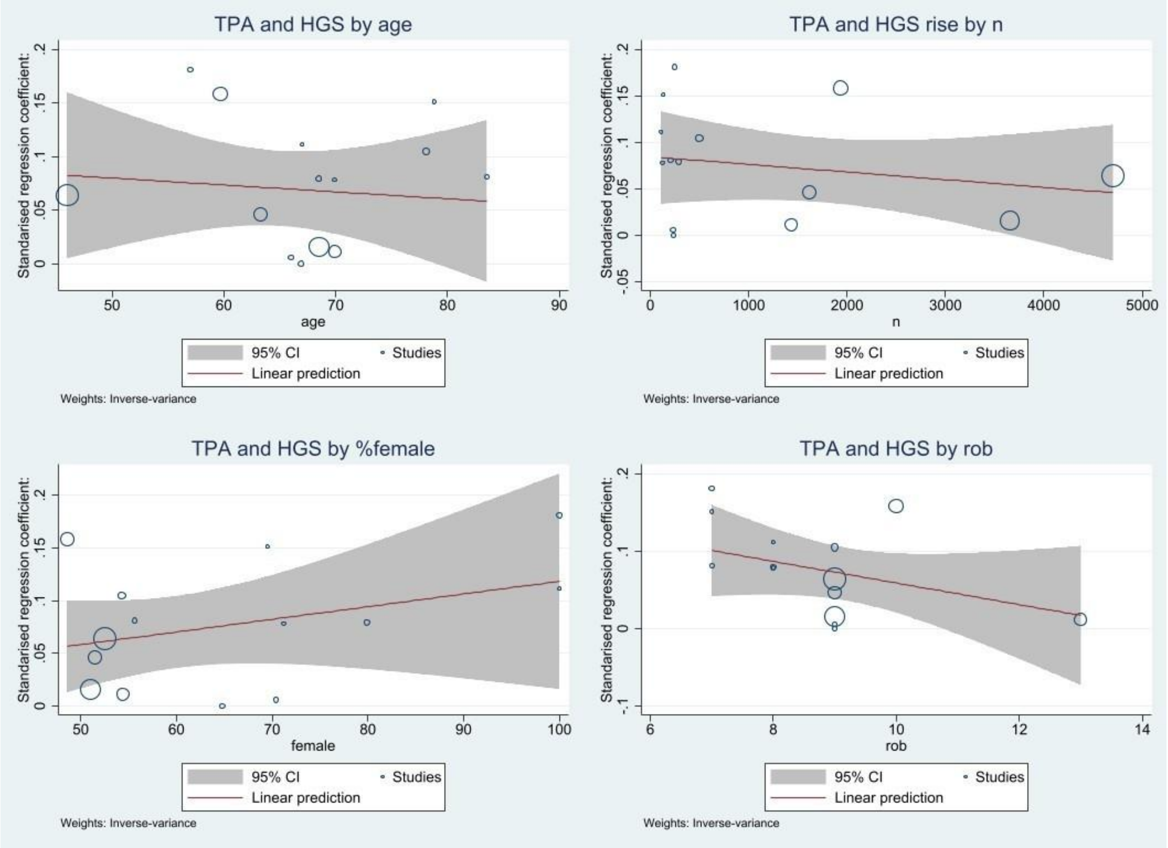

Figure 2. Bubble plots of meta-regression for total physical activity with handgrip strength for; age, sample size (n), percentage of females per study, and risk of bias (quality assessment score)

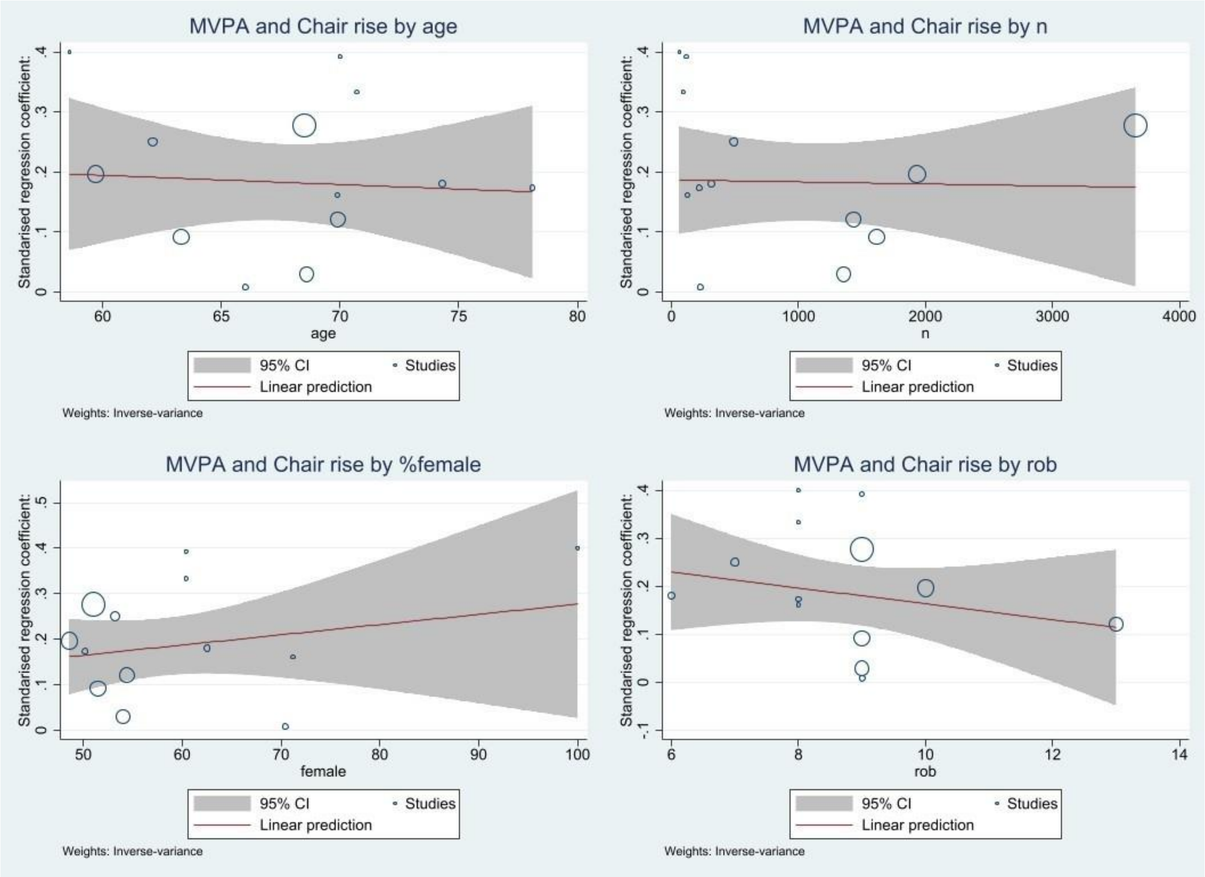

Figure 3. Bubble plots of meta-regression for moderate-to-vigorous physical activity with chair rise for; age, sample size (n), percentage of females per study, and risk of bias (quality assessment score)

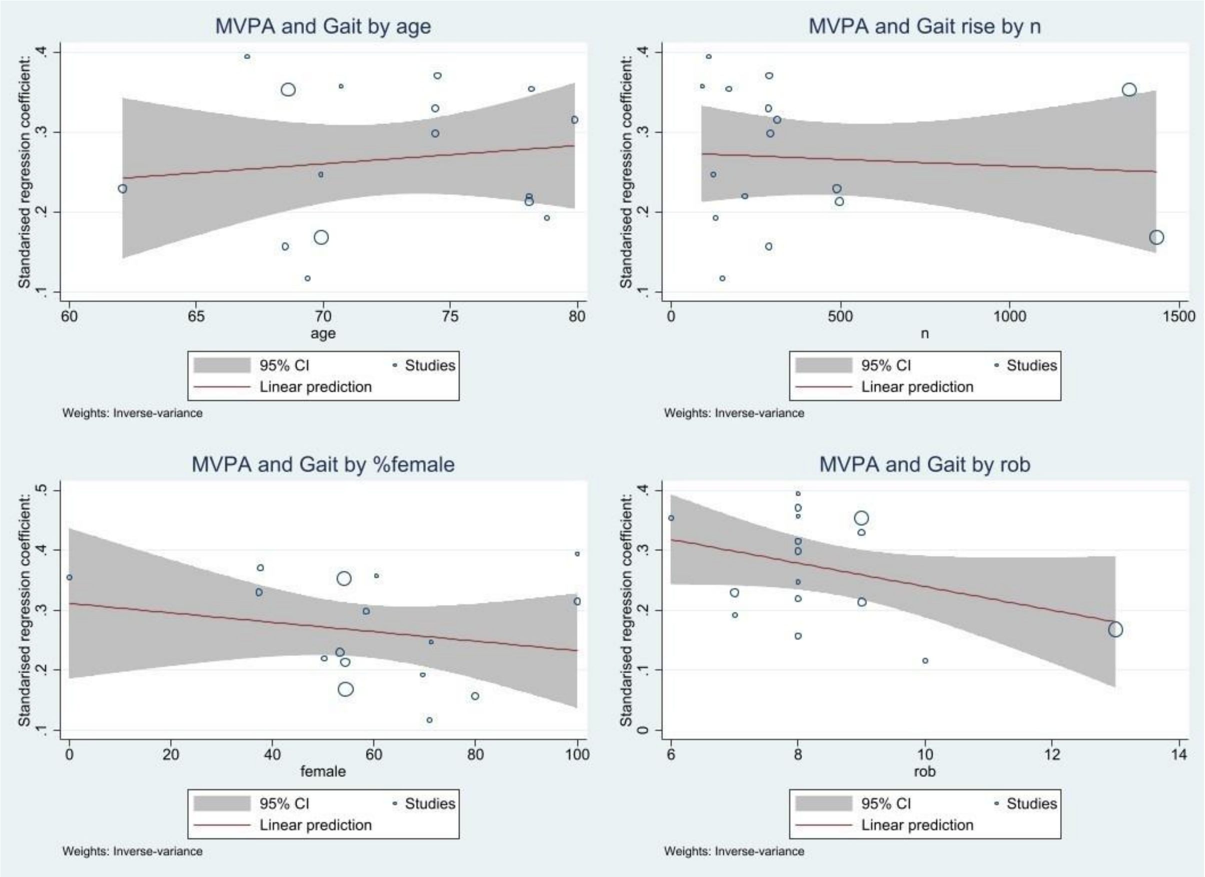

Figure 4. Bubble plots of meta-regression for moderate-to-vigorous physical activity with gait speed for; age, sample size (n), percentage of females per study, and risk of bias (quality assessment score)

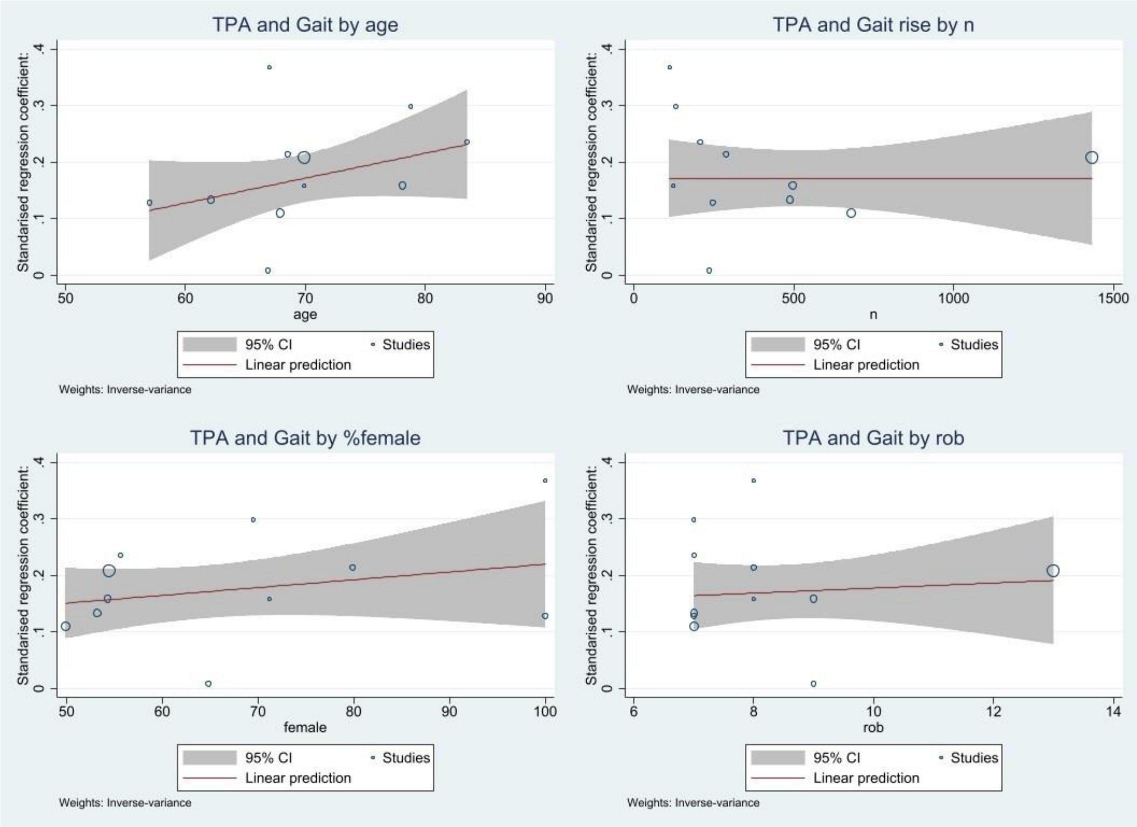

Figure 5. Bubble plots of meta-regression for total physical activity with gait speed for; age, sample size (n), percentage of females per study, and risk of bias (quality assessment score)

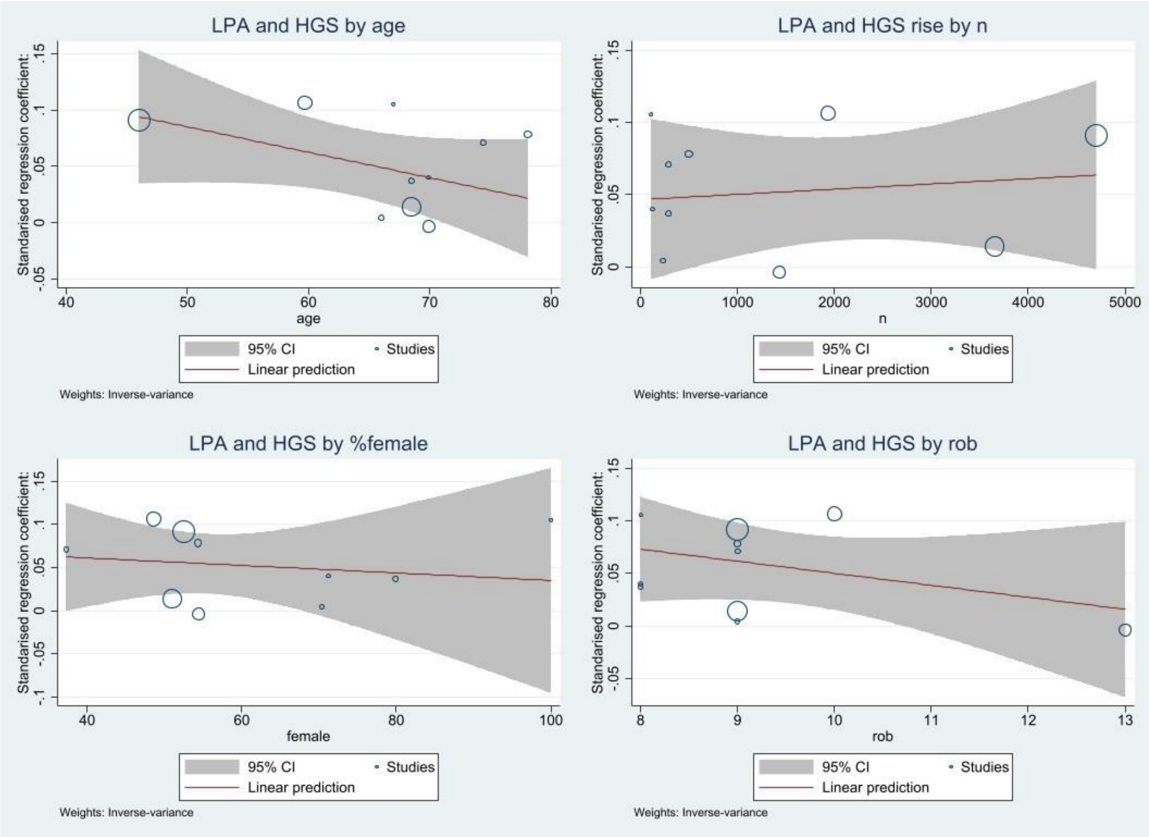

Figure 6. Bubble plots of meta-regression for light physical activity with gait speed for; age, sample size (n), percentage of females per study, and risk of bias (quality assessment score)

(A)

```
  Effect-size label: Standarised regression coefficient:
        Effect size: fisherz
          Std. err.: SE

Random-effects meta-regression                      Number of obs  =        13
Method: REML                                        Residual heterogeneity:
                                                                tau2 =  .01019
                                                              I2 (%) =   88.20
                                                                  H2 =    8.48
                                                     R-squared (%) =    0.00
                                                    Wald chi2(1)   =    0.06
                                                    Prob > chi2    =  0.8063
```

_meta_es	Coefficient	Std. err.	z	P>|z|	[95% conf.	interval]
age	-.0015296	.0062391	-0.25	0.806	-.0137581	.0106988
_cons	.2862545	.4227316	0.68	0.498	-.5422842	1.114793

Test of residual homogeneity: Q_res = chi2(11) = 102.16 Prob > Q_res = 0.0000

(B)

```
  Effect-size label: Standarised regression coefficient:
        Effect size: fisherz
          Std. err.: SE

Random-effects meta-regression                      Number of obs  =        13
Method: REML                                        Residual heterogeneity:
                                                                tau2 =  .01046
                                                              I2 (%) =   87.25
                                                                  H2 =    7.85
                                                     R-squared (%) =    0.00
                                                    Wald chi2(1)   =    0.01
                                                    Prob > chi2    =  0.9125
```

_meta_es	Coefficient	Std. err.	z	P>|z|	[95% conf.	interval]
n	-3.35e-06	.0000305	-0.11	0.912	-.0000631	.0000564
_cons	.1868621	.0469646	3.98	0.000	.0948131	.2789111

Test of residual homogeneity: Q_res = chi2(11) = 67.76   Prob > Q_res = 0.0000

(C)

```
  Effect-size label: Standarised regression coefficient:
        Effect size: fisherz
          Std. err.: SE

Random-effects meta-regression                      Number of obs  =        13
Method: REML                                        Residual heterogeneity:
                                                                tau2 = .009824
                                                              I2 (%) =   88.99
                                                                  H2 =    9.08
                                                     R-squared (%) =    0.00
                                                    Wald chi2(1)   =    0.59
                                                    Prob > chi2    =  0.4439
```

_meta_es	Coefficient	Std. err.	z	P>|z|	[95% conf.	interval]
female	.0022466	.0029346	0.77	0.444	-.003505	.0079983
_cons	.0525061	.1728945	0.30	0.761	-.2863609	.3913731

Test of residual homogeneity: Q_res = chi2(11) = 100.63 Prob > Q_res = 0.0000

(D)

```
  Effect-size label: Standarised regression coefficient:
        Effect size: fisherz
          Std. err.: SE

Random-effects meta-regression                      Number of obs  =        13
Method: REML                                        Residual heterogeneity:
                                                                tau2 = .009458
                                                              I2 (%) =   87.73
                                                                  H2 =    8.15
                                                     R-squared (%) =    0.00
                                                    Wald chi2(1)   =    0.80
                                                    Prob > chi2    =  0.3724
```

_meta_es	Coefficient	Std. err.	z	P>|z|	[95% conf.	interval]
rob	-.0164824	.0184773	-0.89	0.372	-.0526972	.0197323
_cons	.3284359	.1669153	1.97	0.049	.0012878	.6555839

Test of residual homogeneity: Q_res = chi2(11) = 96.46   Prob > Q_res = 0.0000

Figure 7. Meta-regression output for moderate-to-vigorous physical activity with chair rise for; (A) age, (B) sample size, (C) percentage of females per study, and (D) risk of bias (quality assessment score)

(A)

```
  Effect-size label: Standarised regression coefficient:
        Effect size: fisherz
          Std. err.: SE

Random-effects meta-regression                      Number of obs  =        11
Method: REML                                        Residual heterogeneity:
                                                                tau2 = .001855
                                                              I2 (%) =   40.77
                                                                  H2 =    1.69
                                                     R-squared (%) =   16.79
                                                    Wald chi2(1)   =    1.94
                                                    Prob > chi2    =  0.1641
```

_meta_es	Coefficient	Std. err.	z	P>|z|	[95% conf.	interval]
age	.0044013	.0031632	1.39	0.164	-.0017984	.010601
_cons	-.1362307	.221096	-0.62	0.538	-.5695709	.2971095

Test of residual homogeneity: Q_res = chi2(9) = 16.68   Prob > Q_res = 0.0539

(B)

```
  Effect-size label: Standarised regression coefficient:
        Effect size: fisherz
          Std. err.: SE

Random-effects meta-regression                      Number of obs  =        11
Method: REML                                        Residual heterogeneity:
                                                                tau2 = .003481
                                                              I2 (%) =   51.14
                                                                  H2 =    2.05
                                                     R-squared (%) =    0.00
                                                    Wald chi2(1)   =    0.00
                                                    Prob > chi2    =  0.9970
```

_meta_es	Coefficient	Std. err.	z	P>|z|	[95% conf.	interval]
n	-2.22e-07	.0000593	-0.00	0.997	-.0001165	.000116
_cons	.1717266	.0397358	4.32	0.000	.0938458	.2496073

Test of residual homogeneity: Q_res = chi2(9) = 18.41   Prob > Q_res = 0.0307

(C)

```
  Effect-size label: Standarised regression coefficient:
        Effect size: fisherz
          Std. err.: SE

Random-effects meta-regression                      Number of obs  =        11
Method: REML                                        Residual heterogeneity:
                                                                tau2 = .002757
                                                              I2 (%) =   50.51
                                                                  H2 =    2.02
                                                     R-squared (%) =    0.00
                                                    Wald chi2(1)   =    0.89
                                                    Prob > chi2    =  0.3446
```

_meta_es	Coefficient	Std. err.	z	P>|z|	[95% conf.	interval]
female	.0013765	.0014564	0.95	0.345	-.0014779	.0042309
_cons	.0823167	.096629	0.85	0.394	-.1070726	.271706

Test of residual homogeneity: Q_res = chi2(9) = 18.70   Prob > Q_res = 0.0279

(D)

```
  Effect-size label: Standarised regression coefficient:
        Effect size: fisherz
          Std. err.: SE

Random-effects meta-regression                      Number of obs  =        11
Method: REML                                        Residual heterogeneity:
                                                                tau2 = .003025
                                                              I2 (%) =   46.71
                                                                  H2 =    1.88
                                                     R-squared (%) =    0.00
                                                    Wald chi2(1)   =    0.15
                                                    Prob > chi2    =  0.7000
```

_meta_es	Coefficient	Std. err.	z	P>|z|	[95% conf.	interval]
rob	.004495	.011664	0.39	0.700	-.018366	.027356
_cons	.132817	.1023774	1.30	0.195	-.067839	.3334729

Test of residual homogeneity: Q_res = chi2(9) = 17.18   Prob > Q_res = 0.0460

Figure 8. Meta-regression output for total physical activity with gait speed for; (A) age, (B) sample size, (C) percentage of females per study, and (D) risk of bias (quality assessment score)

(A)

```
  Effect-size label: Standarised regression coefficient:
        Effect size: fisherz
          Std. err.: SE

Random-effects meta-regression                     Number of obs  =         10
Method: REML                                       Residual heterogeneity:
                                                              tau2 = .000882
                                                            I2 (%) =   42.12
                                                                H2 =    1.73
                                                    R-squared (%) =   35.33
                                                   Wald chi2(1)   =     2.28
                                                   Prob > chi2    =   0.1308

------------------------------------------------------------------------------
    _meta_es | Coefficient  Std. err.      z    P>|z|     [95% conf. interval]
-------------+----------------------------------------------------------------
         age |  -.0022516   .0014901    -1.51   0.131    -.0051721    .0006688
       _cons |    .197563   .0957764     2.06   0.039     .0098447    .3852814
------------------------------------------------------------------------------
Test of residual homogeneity: Q_res = chi2(8) = 11.76     Prob > Q_res = 0.1624
```

(B)

```
  Effect-size label: Standarised regression coefficient:
        Effect size: fisherz
          Std. err.: SE

Random-effects meta-regression                     Number of obs  =         10
Method: REML                                       Residual heterogeneity:
                                                              tau2 = .001589
                                                            I2 (%) =   60.78
                                                                H2 =    2.55
                                                    R-squared (%) =    0.00
                                                   Wald chi2(1)   =     0.11
                                                   Prob > chi2    =   0.7372

------------------------------------------------------------------------------
    _meta_es | Coefficient  Std. err.      z    P>|z|     [95% conf. interval]
-------------+----------------------------------------------------------------
           n |   3.62e-06   .0000108     0.34   0.737    -.0000175    .0000248
       _cons |   .0465493   .0291841     1.60   0.111    -.0106505    .1037492
------------------------------------------------------------------------------
Test of residual homogeneity: Q_res = chi2(8) = 22.18     Prob > Q_res = 0.0046
```

(C)

```
  Effect-size label: Standarised regression coefficient:
        Effect size: fisherz
          Std. err.: SE

Random-effects meta-regression                     Number of obs  =         10
Method: REML                                       Residual heterogeneity:
                                                              tau2 = .001434
                                                            I2 (%) =   63.50
                                                                H2 =    2.74
                                                    R-squared (%) =    0.00
                                                   Wald chi2(1)   =     0.09
                                                   Prob > chi2    =   0.7636

------------------------------------------------------------------------------
    _meta_es | Coefficient  Std. err.      z    P>|z|     [95% conf. interval]
-------------+----------------------------------------------------------------
      female |  -.0004349   .0014457    -0.30   0.764    -.0032685    .0023987
       _cons |   .0785198    .082466     0.95   0.341    -.0831107    .2401502
------------------------------------------------------------------------------
Test of residual homogeneity: Q_res = chi2(8) = 23.15     Prob > Q_res = 0.0032
```

(D)

```
  Effect-size label: Standarised regression coefficient:
        Effect size: fisherz
          Std. err.: SE

Random-effects meta-regression                     Number of obs  =         10
Method: REML                                       Residual heterogeneity:
                                                              tau2 = .001326
                                                            I2 (%) =   58.22
                                                                H2 =    2.39
                                                    R-squared (%) =    2.76
                                                   Wald chi2(1)   =     0.98
                                                   Prob > chi2    =   0.3215

------------------------------------------------------------------------------
    _meta_es | Coefficient  Std. err.      z    P>|z|     [95% conf. interval]
-------------+----------------------------------------------------------------
         rob |  -.0114224   .0115213    -0.99   0.321    -.0340036    .0111589
       _cons |   .1642439   .1121764     1.46   0.143    -.0556178    .3841056
------------------------------------------------------------------------------
Test of residual homogeneity: Q_res = chi2(8) = 20.48     Prob > Q_res = 0.0087
```

Figure 9. Meta-regression output for light physical activity with handgrip strength for; (A) age, (B) sample size, (C) percentage of females per study, and (D) risk of bias (quality assessment score)

(A)

```
  Effect-size label: Standarised regression coefficient:
        Effect size: fisherz
          Std. err.: SE

Random-effects meta-regression                     Number of obs  =         18
Method: REML                                       Residual heterogeneity:
                                                              tau2 = .002789
                                                            I2 (%) =   69.45
                                                                H2 =    3.27
                                                    R-squared (%) =    6.45
                                                   Wald chi2(1)   =     1.70
                                                   Prob > chi2    =   0.1919

------------------------------------------------------------------------------
    _meta_es | Coefficient  Std. err.      z    P>|z|     [95% conf. interval]
-------------+----------------------------------------------------------------
         age |   .0021139     .00162     1.30   0.192    -.0010613     .005289
       _cons |  -.0698531   .1072536    -0.65   0.515    -.2800662      .14036
------------------------------------------------------------------------------
Test of residual homogeneity: Q_res = chi2(16) = 60.56    Prob > Q_res = 0.0000
```

(B)

```
  Effect-size label: Standarised regression coefficient:
        Effect size: fisherz
          Std. err.: SE

Random-effects meta-regression                     Number of obs  =         18
Method: REML                                       Residual heterogeneity:
                                                              tau2 = .002925
                                                            I2 (%) =   70.27
                                                                H2 =    3.36
                                                    R-squared (%) =    1.91
                                                   Wald chi2(1)   =     1.25
                                                   Prob > chi2    =   0.2629

------------------------------------------------------------------------------
    _meta_es | Coefficient  Std. err.      z    P>|z|     [95% conf. interval]
-------------+----------------------------------------------------------------
           n |  -.0000176   .0000157    -1.12   0.263    -.0000483    .0000132
       _cons |   .0926447   .0275085     3.37   0.001      .038729    .1465603
------------------------------------------------------------------------------
Test of residual homogeneity: Q_res = chi2(16) = 63.56    Prob > Q_res = 0.0000
```

(C)

```
  Effect-size label: Standarised regression coefficient:
        Effect size: fisherz
          Std. err.: SE

Random-effects meta-regression                     Number of obs  =         18
Method: REML                                       Residual heterogeneity:
                                                              tau2 = .002721
                                                            I2 (%) =   68.74
                                                                H2 =    3.20
                                                    R-squared (%) =    8.75
                                                   Wald chi2(1)   =     1.39
                                                   Prob > chi2    =   0.2379

------------------------------------------------------------------------------
    _meta_es | Coefficient  Std. err.      z    P>|z|     [95% conf. interval]
-------------+----------------------------------------------------------------
      female |   .0007143   .0006053     1.18   0.238     -.000472    .0019007
       _cons |   .0278897   .0380723     0.73   0.464    -.0467307    .1025102
------------------------------------------------------------------------------
Test of residual homogeneity: Q_res = chi2(16) = 56.91    Prob > Q_res = 0.0000
```

(D)

```
  Effect-size label: Standarised regression coefficient:
        Effect size: fisherz
          Std. err.: SE

Random-effects meta-regression                     Number of obs  =         18
Method: REML                                       Residual heterogeneity:
                                                              tau2 = .003101
                                                            I2 (%) =   72.62
                                                                H2 =    3.65
                                                    R-squared (%) =    0.00
                                                   Wald chi2(1)   =     1.19
                                                   Prob > chi2    =   0.2746

------------------------------------------------------------------------------
    _meta_es | Coefficient  Std. err.      z    P>|z|     [95% conf. interval]
-------------+----------------------------------------------------------------
         rob |  -.0136958    .012535    -1.09   0.275     -.038264    .0108724
       _cons |    .193879   .1158183     1.67   0.094    -.0331208    .4208788
------------------------------------------------------------------------------
Test of residual homogeneity: Q_res = chi2(16) = 68.20    Prob > Q_res = 0.0000
```

Figure 10. Meta-regression output for moderate-to-vigorous physical activity with handgrip strength for; (A) age, (B) sample size, (C) percentage of females per study, and (D) risk of bias (quality assessment score)

(A)

```
  Effect-size label: Standarised regression coefficient:
        Effect size: fisherz
          Std. err.: SE

Random-effects meta-regression                      Number of obs  =        14
Method: REML                                        Residual heterogeneity:
                                                                tau2 = .002236
                                                              I2 (%) =   62.91
                                                                  H2 =    2.70
                                                      R-squared (%) =    0.00
                                                    Wald chi2(1)    =    0.12
                                                    Prob > chi2     =  0.7267

    _meta_es | Coefficient  Std. err.      z    P>|z|     [95% conf. interval]
-------------+----------------------------------------------------------------
         age |   -.000644   .0018429    -0.35   0.727    -.004256    .0029679
       _cons |   .1121226   .1212493     0.92   0.355   -.1255216    .3497668

Test of residual homogeneity: Q_res = chi2(12) = 35.09   Prob > Q_res = 0.0005
```

(B)

```
  Effect-size label: Standarised regression coefficient:
        Effect size: fisherz
          Std. err.: SE

Random-effects meta-regression                      Number of obs  =        14
Method: REML                                        Residual heterogeneity:
                                                                tau2 = .002162
                                                              I2 (%) =   64.32
                                                                  H2 =    2.80
                                                      R-squared (%) =    0.00
                                                    Wald chi2(1)    =    0.55
                                                    Prob > chi2     =  0.4572

    _meta_es | Coefficient  Std. err.      z    P>|z|     [95% conf. interval]
-------------+----------------------------------------------------------------
           n |  -8.25e-06   .0000111    -0.74   0.457     -.00003    .0000135
       _cons |   .0845119   .0261942     3.23   0.001    .0331722    .1358516

Test of residual homogeneity: Q_res = chi2(12) = 35.15   Prob > Q_res = 0.0004
```

(C)

```
  Effect-size label: Standarised regression coefficient:
        Effect size: fisherz
          Std. err.: SE

Random-effects meta-regression                      Number of obs  =        14
Method: REML                                        Residual heterogeneity:
                                                                tau2 = .002084
                                                              I2 (%) =   67.59
                                                                  H2 =    3.09
                                                      R-squared (%) =    0.00
                                                    Wald chi2(1)    =    0.97
                                                    Prob > chi2     =  0.3254

    _meta_es | Coefficient  Std. err.      z    P>|z|     [95% conf. interval]
-------------+----------------------------------------------------------------
      female |   .0012018   .0012222     0.98   0.325   -.0011936    .0035973
       _cons |  -.0018811   .0751264    -0.03   0.980   -.1491262    .1453639

Test of residual homogeneity: Q_res = chi2(12) = 36.19   Prob > Q_res = 0.0003
```

(D)

```
  Effect-size label: Standarised regression coefficient:
        Effect size: fisherz
          Std. err.: SE

Random-effects meta-regression                      Number of obs  =        14
Method: REML                                        Residual heterogeneity:
                                                                tau2 = .002052
                                                              I2 (%) =   65.50
                                                                  H2 =    2.90
                                                      R-squared (%) =    0.00
                                                    Wald chi2(1)    =    1.57
                                                    Prob > chi2     =  0.2097

    _meta_es | Coefficient  Std. err.      z    P>|z|     [95% conf. interval]
-------------+----------------------------------------------------------------
         rob |  -.0139659   .0111327    -1.25   0.210   -.0357857    .0078538
       _cons |   .1984021   .1038656     1.91   0.056   -.0051707    .4019749

Test of residual homogeneity: Q_res = chi2(12) = 35.65   Prob > Q_res = 0.0004
```

Figure 11. Meta-regression output for total physical activity with handgrip strength for; (A) age, (B) sample size, (C) percentage of females per study, and (D) risk of bias (quality assessment score)

(A)

```
  Effect-size label: Standarised regression coefficient:
        Effect size: fisherz
          Std. err.: SE

Random-effects meta-regression                      Number of obs  =        16
Method: REML                                        Residual heterogeneity:
                                                                tau2 = .004312
                                                              I2 (%) =   60.29
                                                                  H2 =    2.52
                                                      R-squared (%) =    0.00
                                                    Wald chi2(1)    =    0.26
                                                    Prob > chi2     =  0.6098

    _meta_es | Coefficient  Std. err.      z    P>|z|     [95% conf. interval]
-------------+----------------------------------------------------------------
         age |   .0022648   .0044372     0.51   0.610    -.006432    .0109617
       _cons |   .1017223   .3221858     0.32   0.752   -.5297502    .7331949

Test of residual homogeneity: Q_res = chi2(14) = 42.46   Prob > Q_res = 0.0001
```

(B)

```
  Effect-size label: Standarised regression coefficient:
        Effect size: fisherz
          Std. err.: SE

Random-effects meta-regression                      Number of obs  =        16
Method: REML                                        Residual heterogeneity:
                                                                tau2 = .004534
                                                              I2 (%) =   59.50
                                                                  H2 =    2.47
                                                      R-squared (%) =    0.00
                                                    Wald chi2(1)    =    0.11
                                                    Prob > chi2     =  0.7366

    _meta_es | Coefficient  Std. err.      z    P>|z|     [95% conf. interval]
-------------+----------------------------------------------------------------
           n |  -.0000167   .0000498    -0.34   0.737   -.0001143    .0000808
       _cons |   .2742415   .0338929     8.09   0.000    .2078127    .3406704

Test of residual homogeneity: Q_res = chi2(14) = 42.36   Prob > Q_res = 0.0001
```

(C)

```
  Effect-size label: Standarised regression coefficient:
        Effect size: fisherz
          Std. err.: SE

Random-effects meta-regression                      Number of obs  =        16
Method: REML                                        Residual heterogeneity:
                                                                tau2 = .004089
                                                              I2 (%) =   60.08
                                                                  H2 =    2.51
                                                      R-squared (%) =    0.00
                                                    Wald chi2(1)    =    0.58
                                                    Prob > chi2     =  0.4452

    _meta_es | Coefficient  Std. err.      z    P>|z|     [95% conf. interval]
-------------+----------------------------------------------------------------
      female |  -.0007869   .0010307    -0.76   0.445   -.0028069    .0012332
       _cons |   .3113835   .0638756     4.87   0.000    .1861897    .4365774

Test of residual homogeneity: Q_res = chi2(14) = 41.60   Prob > Q_res = 0.0001
```

(D)

```
  Effect-size label: Standarised regression coefficient:
        Effect size: fisherz
          Std. err.: SE

Random-effects meta-regression                      Number of obs  =        16
Method: REML                                        Residual heterogeneity:
                                                                 tau2 = .00322
                                                              I2 (%) =   49.90
                                                                  H2 =    2.00
                                                      R-squared (%) =   19.09
                                                    Wald chi2(1)    =    2.70
                                                    Prob > chi2     =  0.1001

    _meta_es | Coefficient  Std. err.      z    P>|z|     [95% conf. interval]
-------------+----------------------------------------------------------------
         rob |  -.0196365   .0119405    -1.64   0.100   -.0430395    .0037666
       _cons |   .4357582   .1057488     4.12   0.000    .2284944     .643022

Test of residual homogeneity: Q_res = chi2(14) = 30.14   Prob > Q_res = 0.0073
```

Figure 12. Meta-regression output for moderate-to-vigorous physical activity with gait speed for; (A) age, (B) sample size, (C) percentage of females per study, and (D) risk of bias (quality assessment score)
