# Supplementary material for: Associations between device-measured physical activity and performance-based physical function outcomes in adults: a systematic review and meta-analysis
Source: BMJ Public Health. 2023 Oct 30;1(1):e100000. doi: 10.1136/bmjph-2023-100000 (PMC11812739; doi:10.1136/bmjph-2023-100000)
Supplement: online supplemental file 7 [file bmjph-1-1-s007.pdf]

```
. *Chair_mvpa
. meta bias if pa==2 & pf==1, egger

  Effect-size label: Standarised regression coefficient:
        Effect size: fisherz
          Std. err.: SE

Regression-based Egger test for small-study effects
Random-effects model
Method: REML

H0: beta1 = 0; no small-study effects
            beta1 =      1.61
      SE of beta1 =     0.974
                z =      1.65
       Prob > |z| =    0.0993
```

(A)

```
. meta bias if pa==0 & pf==2, egger

  Effect-size label: Standarised regression coefficient:
        Effect size: fisherz
          Std. err.: SE

Regression-based Egger test for small-study effects
Random-effects model
Method: REML

H0: beta1 = 0; no small-study effects
            beta1 =      1.26
      SE of beta1 =     1.192
                z =      1.06
       Prob > |z| =    0.2899
```

(B)

```
. meta bias if pa==2 & pf==2, egger

  Effect-size label: Standarised regression coefficient:
        Effect size: fisherz
          Std. err.: SE

Regression-based Egger test for small-study effects
Random-effects model
Method: REML

H0: beta1 = 0; no small-study effects
            beta1 =      0.48
      SE of beta1 =     1.023
                z =      0.47
       Prob > |z| =    0.6378
```

(C)

```
. meta bias if pa==0 & pf==0, egger

  Effect-size label: Standarised regression coefficient:
        Effect size: fisherz
          Std. err.: SE

Regression-based Egger test for small-study effects
Random-effects model
Method: REML

H0: beta1 = 0; no small-study effects
            beta1 =      0.53
      SE of beta1 =     0.699
                z =      0.76
       Prob > |z| =    0.4448
```

(D)

```
. meta bias if pa==1 & pf==0, egger

  Effect-size label: Standarised regression coefficient:
        Effect size: fisherz
          Std. err.: SE

Regression-based Egger test for small-study effects
Random-effects model
Method: REML

H0: beta1 = 0; no small-study effects
            beta1 =     -0.06
      SE of beta1 =     0.784
                z =     -0.08
       Prob > |z| =    0.9373
```

(E)

```
. meta bias if pa==2 & pf==0, egger

  Effect-size label: Standarised regression coefficient:
        Effect size: fisherz
          Std. err.: SE

Regression-based Egger test for small-study effects
Random-effects model
Method: REML

H0: beta1 = 0; no small-study effects
            beta1 =      0.69
      SE of beta1 =     0.677
                z =      1.01
       Prob > |z| =    0.3106
```

(F)

Figure 1. Egger's test output for the associations between; (A) moderate-to-vigorous physical activity and chair rise (B) total physical activity and gait speed (C) moderate-to-vigorous physical activity and gait speed (D) total physical activity and handgrip strength (E) light physical activity and handgrip strength (F) moderate-to-vigorous physical activity and handgrip strength

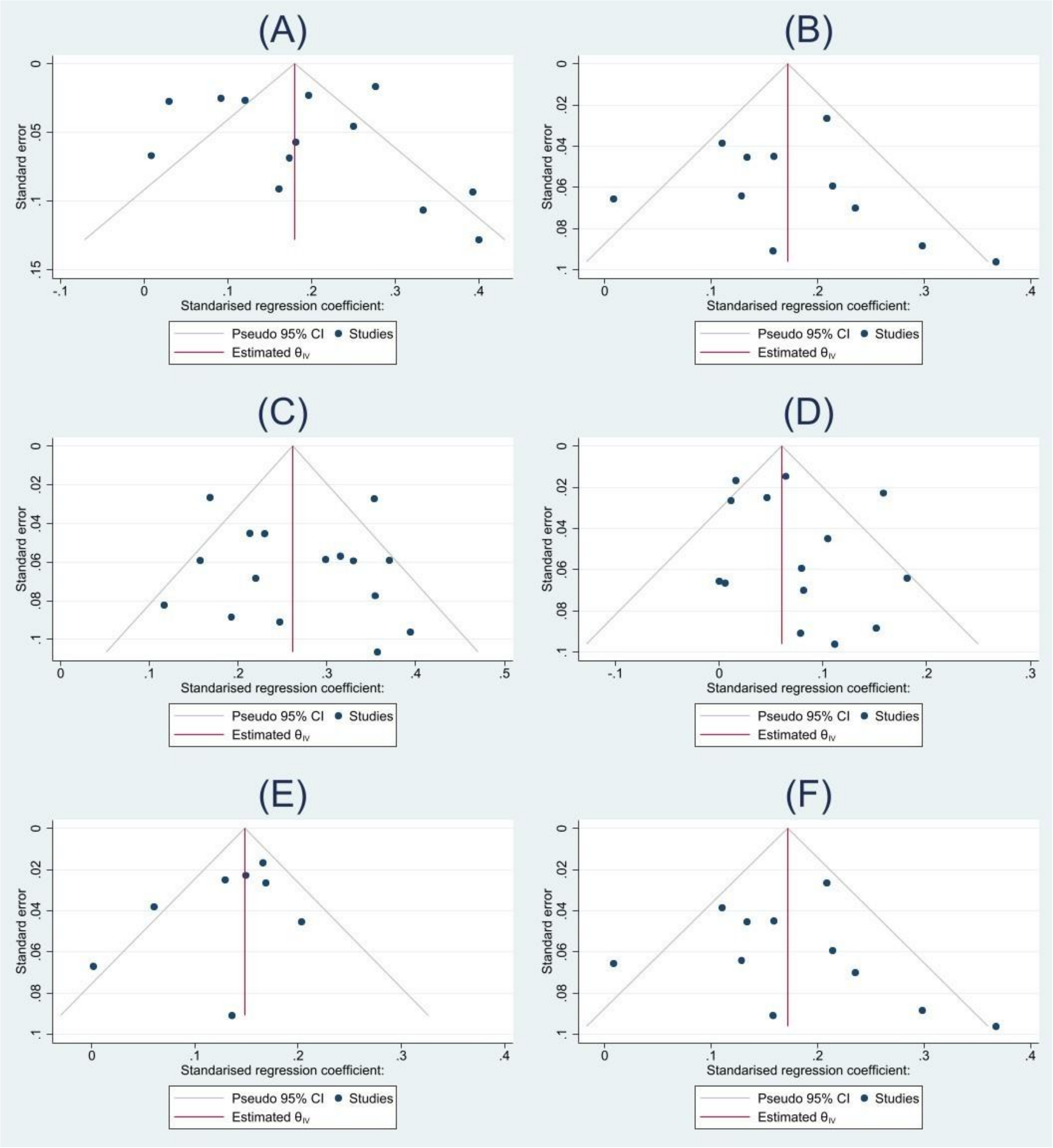

Figure 2. Bubble plots, visual output of Egger's tests for the associations between; (A) moderate-to-vigorous physical activity and chair rise (B) total physical activity and gait speed (C) moderate-to-vigorous physical activity and gait speed (D) total physical activity and handgrip strength (E) light physical activity and handgrip strength (F) moderate-to-vigorous physical activity and handgrip strength
